# Theories for interventions to reduce physical and verbal abuse: A mixed methods review of the health and social care literature to inform future maternity care

**DOI:** 10.1371/journal.pgph.0001594

**Published:** 2023-04-24

**Authors:** Soo Downe, Rebecca Nowland, Andrew Clegg, Naseerah Akooji, Cath Harris, Alan Farrier, Lisa Tanyaradzwa Gondo, Kenny Finlayson, Gill Thomson, Carol Kingdon, Hedieh Mehrtash, Rebekah McCrimmon, Özge Tunçalp

**Affiliations:** 1 Research in Childbirth and Health Group, THRIVE Centre, University of Central Lancashire, Preston, United Kingdom; 2 Maternal and Infant Nurture and Nutrition Group, THRIVE Centre, University of Central Lancashire, Preston, United Kingdom; 3 Synthesis, Economic Evaluations and Decision Science (SEEDS) Group, University of Central Lancashire, Preston, United Kingdom; 4 Lancashire Clinical Trials Unit, University of Central Lancashire, Preston, United Kingdom; 5 Healthy and Sustainable Settings Unit, University of Central Lancashire, Preston, United Kingdom; 6 School of Medicine, University of Central Lancashire, Preston, United Kingdom; 7 UNDP/UNFPA/UNICEF/WHO/World Bank Special Programme of Research, Development and Research Training in Human Reproduction, Department of Sexual and Reproductive Health and Research, World Health Organization, Geneva, Switzerland; 8 School of Community Health and Midwifery, University of Central Lancashire, Preston, United Kingdom; Jhpiego, UNITED STATES

## Abstract

Despite global attention, physical and verbal abuse remains prevalent in maternity and newborn healthcare. We aimed to establish theoretical principles for interventions to reduce such abuse. We undertook a mixed methods systematic review of health and social care literature (MEDLINE, SocINDEX, Global Index Medicus, CINAHL, Cochrane Library, Sept 29th 2020 and March 22^nd^ 2022: no date or language restrictions). Papers that included theory were analysed narratively. Those with suitable outcome measures were meta-analysed. We used convergence results synthesis to integrate findings. In September 2020, 193 papers were retained (17,628 hits). 154 provided theoretical explanations; 38 were controlled studies. The update generated 39 studies (2695 hits), plus five from reference lists (12 controlled studies). A wide range of explicit and implicit theories were proposed. Eleven non-maternity controlled studies could be meta-analysed, but only for physical restraint, showing little intervention effect. Most interventions were multi-component. Synthesis suggests that a combination of systems level and behavioural change models might be effective. The maternity intervention studies could all be mapped to this approach. Two particular adverse contexts emerged; social normalisation of violence across the socio-ecological system, especially for ‘othered’ groups; and the belief that mistreatment is necessary to minimise clinical harm. The ethos and therefore the expression of mistreatment at each level of the system is moderated by the individuals who enact the system, through what they feel they can control, what is socially normal, and what benefits them in that context. Interventions to reduce verbal and physical abuse in maternity care should be locally tailored, and informed by theories encompassing all socio-ecological levels, and the psychological and emotional responses of individuals working within them. Attention should be paid to social normalisation of violence against ‘othered’ groups, and to the belief that intrapartum maternal mistreatment can optimise safe outcomes.

## Introduction

In 2010, Bowser and Hill published a landscape analysis reporting on disrespect and abuse of women during childbirth [1]. In 2015, WHO published a review that classified disrespect and abuse into seven domains, under the overall typology of ‘mistreatment during childbirth’ [2]. These domains include verbal and physical abuse. Since the publication of these papers, there has been increasing evidence in this field, relating to both women and newborns [3–5]. As a result, efforts to improve maternal health are now focused on improving quality of both the provision and experience of maternity services as a critical component of Universal Health Coverage (UHC), particularly in LMIC settings. The current WHO recommendations on maternity care for a positive childbirth experience [6–8] emphasize the overarching importance of respectful maternity care. This includes the need for staff and health services to create enabling maternity environments to encourage a woman’s sense of control and their involvement in decision-making. Improving experiences of women during maternity care requires both promotion of respectful care, and reduction of mistreatment [1, 2]. In 2018, a systematic review of controlled studies undertaken in low-income settings and designed to increase respectful care was undertaken by some of the authors of the current review [9]. The findings indicated that multifactored approaches to increase respectful care could work in low-income settings. Based on the three findings with moderate confidence, we concluded that effective implementation to increase respectful care requires:

*… a visible*, *sustained*, *and participatory intervention process*, *with committed facility leadership*, *management support*, *and staff engagement*. *It is still unclear precisely which elements of a package of RMC [respectful maternity care] implementation might be most successful*, *and most sustained over time…*.

As part of the move to promote respectful care, WHO has been working on improving the evidence globally in relation to measuring the prevalence of mistreatment of women during childbirth and raising awareness for evidence and action [10]. In 2019 the team published a paper reporting on a prospective cross-sectional study of women from admission in labour and up to two hours postpartum in twelve facilities across four countries (Ghana, Guinea, Myanmar, and Nigeria) [11]. Some degree of physical or verbal abuse or stigma/discrimination was observed in 838 (41·6%) of 2016 women, and reported by 945 (range 4–35% by facility) of those surveyed after their birth. Physical and verbal abuse was particularly prevalent in the half hour before birth and the 15 minutes afterwards, and verbal abuse was more likely to be experienced by younger than older women (≥30 years), adjusting for marital status and parity [11]. These findings suggest that little had changed in the ten years since Bowser and Hill first highlighted the issue of abuse in maternity care, despite a significant increase in studies in this area. Looking beyond the reproductive health literature may provide new insights for future effective interventions.

Issues of mistreatment have been identified in other health and social care fields [12]. In particular, there is a body of literature on de-implementation of inappropriate use of physical and pharmacological restraint in nursing and residential services [13], that mirrors current debates about use of unnecessary or unconsented physical and pharmacological interventions in maternity care. Indeed, use of physical restraint on women using maternity services has been reported most recently in 2020 [14, 15].

Although there may be some particular drivers for mistreatment in maternity care (including gender based inequalities, and social norms about desirable reproduction and undesirable reproduction) most underlying factors for mistreatment of service users in health and social care are likely to be similar across disciplines. To date, however, there have been no studies that explore the issue of mistreatment in general, and verbal and physical abuse in particular, in a cross-disciplinary review.

This paper reports on a mixed-methods systematic review of theoretical insights into what might underpin verbal and/or physical abuse in health and social care in general, and of interventions designed to reduce such abuse, with the aim of identifying candidate theories for designing future intervention studies. The findings are synthesised to generate hypotheses about the optimal theoretical underpinning for future implementation of change in this area, and the synthesis is then mapped to published intervention studies in maternity care.

### Aim

To establish theoretical principles and mechanisms of what works in reducing physical and verbal abuse in health and social care, as a basis for designing effective interventions for maternity services in the future.

### Research questions

1. *What theoretical explanations have been proposed to explain drivers for and/or prevention of physical and verbal mistreatment by professional health and social care providers*?
2. *What interventions are effective*, *feasible and acceptable for reducing physical and/or verbal mistreatment of service users by professional providers of health and social care*, *when compared to usual care*, *or to alternative interventions*?
3. *What kinds of theoretically informed interventions work*, *or might work*, *in a range of contexts*, *to reduce physical and verbal abuse of childbearing women by health care providers*?

### Definitions

We used the following definitions of physical and verbal abuse, adapted from the Bohren [2] typology of mistreatment of women during childbirth, with additional terms that are relevant to other areas of health and social care, based on the results of our initial scoping review:

*Verbal abuse*
  Harsh or rude language; shouting, insults, scolding, mocking; judgemental or accusatory comments; threats of withholding treatment or of using unnecessary treatment, or of poor outcomes; blaming for current situation, or current or potential future poor outcomes.
*Physical abuse*
  Being beaten, slapped, kicked, punched, or pinched; physical restraint; gagged; physically tied down; (childbearing women: forceful downward pressure); rough use of instruments or interventions.

### Reflexive statement

We maintained a reflexive stance throughout the review process, from study selection to data synthesis. Progress was discussed regularly among the team and decisions were explored critically. As a review team, we have a mixture of clinical, public health, and other backgrounds, including in midwifery (SD); medicine (LG, OT); psychology (GT, RN); public health (OT, HM); information science (CH), statistics (NA, HM) health services research (KF, CK); health technology assessment (AC). GT, SD, OT, KF had all undertaken prior primary and/or secondary studies in the area of respectful care, mistreatment, birth trauma, and/or maternal mental health, for maternity care users in general, or for marginalised groups specifically, prior to undertaking this review. LG had prior exposure to reproductive justice research. Based on our collective and individual experiences (as clinicians, academics and researchers, as well as health service users), we anticipated that the findings of our review would reveal that relational theories might underpin more effective interventions, and that multiple components, tailored to context, might be most effective, acceptable, and feasible. As a team, we remained aware of these prior beliefs, and we used disconfirmation checks to ensure that we were not over-interpreting data that supported our prior views, or that we were not overlooking data that disputed our pre-suppositions.

## Materials and methods

We undertook a mixed-methods review. A scoping search was initially undertaken to refine the search terms, and to finalise the data sources, prior to formal searches being undertaken.

Once the included papers were located, those reporting maternity interventions were removed from the initial analysis. This was because our first step was to identify potentially transferable insights that were new to the maternity field. The remaining papers then were separated into those that included implicit or explicit reference to any relevant theory/ies, without reporting any interventions; and those that included interventions (with or without reference to theory). We then undertook the following steps:

1. Logging and narrative description of explicit/implicit theories
2. Meta-analysis of the findings in the non-maternity intervention papers with respect to any underlying theory used
3. Synthesis of the results of the first two steps
4. Mapping of the maternity intervention studies against the emerging synthesis
5. Creation of a logic model from the findings and synthesis

### Search strategy

We undertook an initial scoping search in Medline using search terms proposed in the protocol. The search strategy was iteratively refined through testing in the Medline, SocINDEX and Global Index Medicus databases, and consultation with the review team, to improve the precision of the search and identify additional useful terms.

We initially searched the following databases on 29th Sept 2020 using the finalised search strategy: Medline (Ovid); SocINDEX (EBSCO); Global Index Medicus (including African Index Medicus (AIM), Index Medicus for the Eastern Mediterranean Region (IMEMR), Index Medicus for South-East Asia Region (IMSEAR), Latin America and the Caribbean Literature on Health Sciences (LILACS), ans Western Pacific Region Index Medicus (WPRIM)); CINAHL Complete (EBSCO) and the Cochrane Database of Systematic reviews and Central Register of Controlled Trials (CENTRAL) (Cochrane Library). Given the high yield of results retrieved in these databases, and potential overlap of content, the decision was made not to expand the search to further databases originally considered in the protocol. No date or language limits were applied to the search. The search was updated on March 22^nd^ 2022. The full search strategy for the Medline database (Ovid platform) is provided in S1 Table. A log was set up to record hits and numbers of included papers at each stage of selection. One member of the review team (CH) undertook the searches in each database and carried out de-duplication. The de-duplicated results were imported into Rayyan for screening.

### Study selection

At the title and abstract stage of both searches regular meetings were undertaken with the whole team to ensure consistency in decision making. At the full text stage, selection was initially undertaken in three pairs. Calibration exercises were conducted within each pair, where screeners independently screened 100 hits in batches until an 80% level of agreement was reached within the pair. Where sufficient agreement was not reached after the first 100 hits because of areas of uncertainty, this was discussed in the wider team, and the inclusion process was refined by consensus. This process continued until sufficient agreement was achieved. The remaining studies were then screened by four members of the review team (RN, AF, LG, HM) independently for the index search, and by SD and HM in the updated search, with consultation between team members where decisions could not be easily made.

Studies in languages that could not be translated sufficiently by the author team, google translate, or other contributors were logged but not included in the analysis.

As the selection process proceeded, it became apparent that there are a very large number of studies and papers focused on restraint and seclusion in psychiatric care. It was not always easy to determine if the issue was about therapeutic methods of restraint, or about restraint as abuse. To avoid overwhelming the review with papers from this very specific field, the decision was made to exclude papers on seclusion (as this was not deemed to be either verbal or physical abuse) and also to exclude those on medication in psychiatric settings as a means of restraint. We also excluded mechanical restraint in these settings, though we did retain papers on mechanical restraint in critical illness settings, as these were agreed to be more like the kinds of settings in which such restraint might be used in maternity care. For example, women in labour may be mechanically restrained if they have had a sedative (to prevent falls), or their movement may be constrained by IV lines, lithotomy, or wired fetal monitoring machines. See Table 1 for inclusion and exclusion criteria.

**Table 1:** Inclusion and exclusion criteria.

Inclusion	Exclusion
Published research studies and theoretical analyses	Grey literature, PhD or masters theses, commentaries, blogs, media reports
Papers and studies with a theoretical component in the analysis or interpretation OR prospective intervention studies with ‘usual practice’ controls, including time-series designs, or with comparator interventions	Descriptions/prevalence of factors associated with verbal/physical mistreatment, with no theoretical analysis of underlying issues Intervention studies without controls or comparators
Focus on physical or verbal mistreatment in health and social care provision settings/situations	Focus on understanding or explaining positive communication, or interventions focused on other aspects of mistreatment where verbal or physical mistreatment cannot be disaggregated
Focus on professional carers (people paid to provide care)	Focus on lay (voluntary/unpaid) carers, and family or group relations in general, or service user mistreatment of staff
Any health or social care discipline	Study or paper not specifically applied to health and social care
Any study type	
Any language	
Any date	

The intervention design and outcomes of controlled intervention studies in maternity care published since our previous systematic review of such studies [9] were tabulated and assessed against the synthesised theoretical framework of the current review, to establish the extent to which these interventions have been aligned, or not, to the findings of the review.

Studies and papers based in maternity care settings that included implicit or explicit theory were, however, included in the primary theoretical analysis.

### Quality assessment

Risk of bias ratings were applied to studies included in the meta-analysis [16] There is no quality assessment tool for theoretical data, and, indeed, epistemologically this would not be relevant. Though the protocol stated that quality assessment would be undertaken for qualitative studies, it was decided in the team that this was not required, since the findings were not the unit of analysis: the issue of interest was the explicit and implicit theories that were evident in the included studies.

### Record of study characteristics

For the research studies and articles including theoretical concepts, relevant data from all included full-text studies were logged on a study-specific data capture form. This included the bibliographic details, and characteristics of included papers; aim of study, participant characteristics (where relevant) implicit, explicit theories, other explanations of mistreatment; and findings from research papers. The form also captured the health/social care domain(s) of each paper, and the type of mistreatment that was addressed.

For the controlled studies, data were extracted using a pre-piloted form by one reviewer and checked by a second reviewer. Disagreements were discussed between reviewers and, if consensus was not achieved, arbitration was carried out by a third reviewer. We extracted data on the citation details, country of study, study design, data collection method, primary outcome, secondary outcomes, intervention, underpinning theory, setting, type of mistreatment, participants characteristics, primary and secondary outcomes, and methods of data collection and analysis.

## Analysis

### Studies including theory

We intended to undertake an adaptation of the meta-narrative approach [17] to track theories used by different disciplines over time and to create an initial taxonomy of candidate theories that could underpin intervention studies to reduce physical and verbal abuse. In the event, very few studies included formal underpinning theory, so this analysis could not be undertaken. Our approach was to identify author-identified (‘explicit’) a-priori or post-hoc theories cited in the studies and to identify sections of the text of the included papers that referred to implicit or explicit theoretical concepts or models, or drivers for verbal or physical mistreatment. Both explicit and implicit theories were logged. We summarised these findings narratively.

#### Controlled intervention studies

Our outcome of interest was any reduction in any measure of physical or verbal abuse (primary). We also looked for information on the acceptability of the intervention(s) for staff or service users, fidelity to the protocol, and for any evidence of sustainability.

RCT and before and after (‘controlled’) intervention studies that had measures of relevance to the review were logged. Where possible, meta-analysis was undertaken to assess the effectiveness of the intervention. We pooled studies through the inverse variance method, estimating the standardised mean difference (SMD) with 95% confidence intervals, using random effects models.

Heterogeneity was assessed through visual inspection of the forest plots and the I^2^ statistic. Pre-specified sub-group analyses were produced, focusing on study design, underpinning theory, care setting, people receiving the intervention, type of intervention and country income level. Sensitivity analyses explored the effects of different studies on the outcomes.

We also intended to use network meta-analysis techniques [18], if the data were robust enough, to identify pathways that stood out as highly influential, or contexts that tended to be associated with the success or otherwise of particular theories. In the event, this was not possible due to insufficient data of adequate precision to develop an evidence network.

### Synthesis and logic model

We used a results-based convergent synthesis to integrate the findings across the papers with theoretical elements and intervention studies [19], as a means of answering our third research question. This approach is defined by Hong as: *where qualitative and quantitative evidence is analyzed separately using different synthesis methods and results of both syntheses are integrated during a final synthesis* [19]. The synthesis was intended to illuminate what fundamental theoretical approach(es) might have most utility as a basis for designing interventions to reduce physical or verbal mistreatment in a range of contexts. We then mapped the resulting model to controlled studies in maternity service provision to assess the extent to which our model is reflected currently in interventions used in controlled studies designed to reduce verbal and/or physical abuse in maternity care. Finally, we created a logic model as the basis for hypotheses about critical contexts and mechanisms of effect arising from our findings.

### Changes from protocol

Changes from the protocol are described above.

## Results

**Fig 1:**
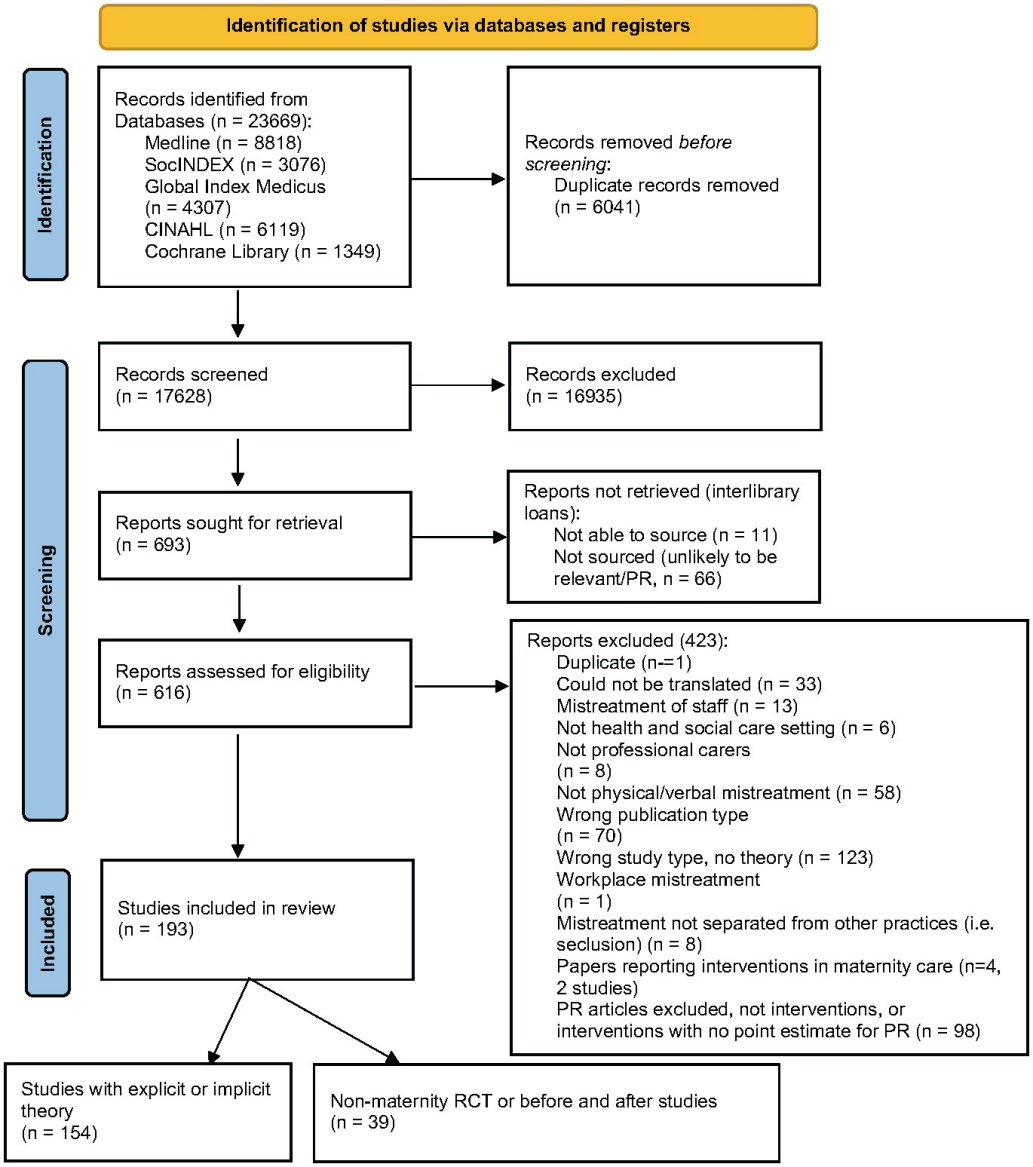
PRISMA flow chart, index search (29^th^ Sept 2020). *From*: Page MJ, McKenzie JE, Bossuyt PM, Boutron I, Hoffmann TC, Mulrow CD, et al. The PRISMA 2020 statement: an updated guideline for reporting systematic reviews. BMJ 2021;372:n71. doi: 10.1136/bmj.n71.

In the primary search (2020), a total of 23,699 studies were identified from the database searches. Following de-duplication 17,628 were retained for screening. Screening on title/abstract revealed 693 relevant studies for full text review. Many of these were focused on physical restraint in a range of settings. Ninety of them required inter-library loan requests, and a decision was made to only pursue a sub-set that were intervention studies, or that were not physical restraint studies (n = 24). Eleven of these could not be sourced, leaving 616 for full text screening selection. During full text screening it became apparent that a very large proportion of the remaining studies meeting the inclusion criteria were also focused on physical restraint. At that point, it was decided that the remaining physical restraint papers would be excluded, unless they were intervention studies, or studies with a strong theoretical basis. During closer scrutiny by the quantitative team, thirteen of the RCT or before and after studies of restraint did not have identifiable point estimate measures of restraint use, and so these were also excluded. Ninety-eight of 150 physical restraint papers were therefore excluded at this stage. A further 324 papers were excluded for a range of reasons (Fig 1).

The remaining 193 included papers were grouped into those including theoretical components (n = 154 [20–173]) (qualitative and quantitative and reviews) and (n = 39 [174–212]) non-maternity controlled intervention studies with at least one outcome measure of verbal or physical abuse.

**Fig 2:**
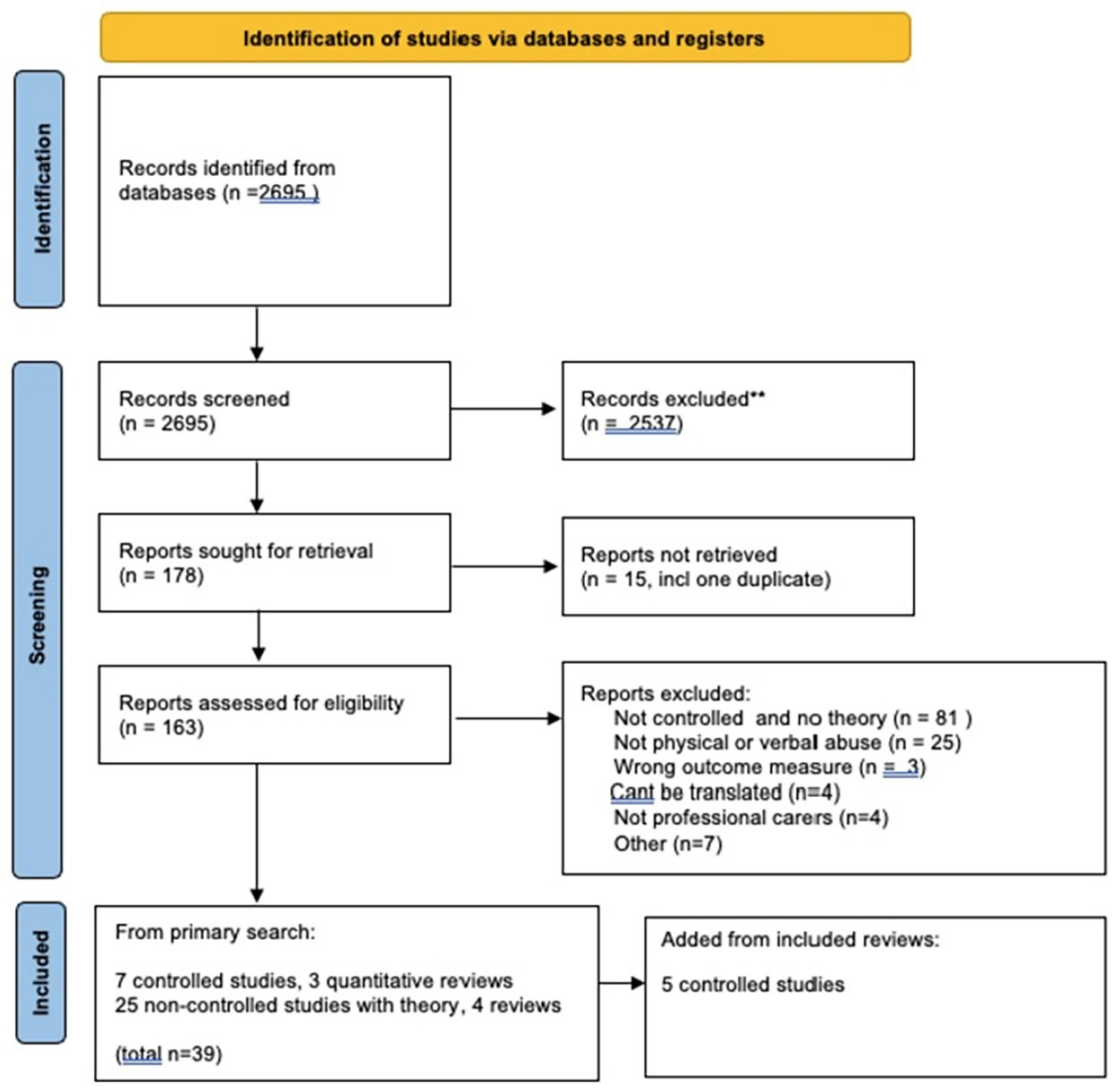
PRISMA flow chart, updated search (22^nd^ March 2022). *From*: Page MJ, McKenzie JE, Bossuyt PM, Boutron I, Hoffmann TC, Mulrow CD, et al. The PRISMA 2020 statement: an updated guideline for reporting systematic reviews. BMJ 2021;372:n71. doi: 10.1136/bmj.n71.

In the updated search (2022), of 2695 hits from the updated search, 29 had theoretical components [213–241], including 4 reviews [217, 218, 221, 233] and 14 non-maternity controlled studies with at least one measure of verbal or physical abuse [242–255] (Fig 2).

Six of these were controlled studies, based in emergency/ITU/critical care[246, 253, 254] inpatient geriatric care [243]; a youth behavioural unit [247] and adult psychiatric care [255]. Three quantitative reviews were included [242, 245, 252] and their reference lists generated five of the included primary studies [244, 248–251]. Two of the included studies could be metaanalysed [248, 253].

In the updated inclusion decision process, we did not check for primary papers included in the previously published reviews that had not been identified by our search, given that the primary papers in the previous reviews were not selected for their theoretical content. However, we did check to ensure there was no substantial overlap between the papers contributing to the previous reviews and our primary theory papers, to avoid ‘double weighting’ of the overall contribution of the prior reviews to our analysis.

The three included reviews of controlled studies [242, 245, 252] and two of the primary controlled papers [243, 244] also included some theory. They are included in the analysis of theory papers, resulting in a total of 34 papers with theoretical components. The results of both searches are combined in the rest of this paper.

### Papers with underlying theoretical components (n = 188)

The characteristics of the 188 papers with explicit or implicit underlying theories in the study design, analysis, interpretation or discussion from the first (n = 154) and second (n = 34) reviews are provided in S2 and S3 Tables. The date of publication ranged from 1988–2022, and they were undertaken in all regions of the world. For maternity focused papers with theory, 48/58 were based in low or middle income countries [20, 22, 26, 30, 31, 38, 39, 44, 46, 53, 54, 59, 60, 63, 75, 100–102, 120, 122, 124, 140, 148, 150, 151, 154, 156, 157, 159–161, 167, 171–173, 217, 219–222, 224, 225, 226, 229, 230, 235, 236], with larger numbers of studies from Ethiopia [31, 38, 59, 60, 154, 221], India [44, 75, 120, 124, 154, 157, 198], Kenya [20, 22, 140, 171, 172] Tanzania [148, 150, 156, 160] and Brazil [161, 222, 225, 226, 230]. In contrast, the vast majority of the 130 studies based in other areas of health care took place in high income countries, with large numbers from North America [23, 28, 32, 40, 48, 55, 66–69, 82–84, 91, 96, 105, 106, 113, 129, 130, 132, 143, 145, 146, 153, 163, 165, 218, 239, 242–244].

The topics included mistreatment in maternity settings (n = 58) [19, 21, 24, 26, 30, 31, 38,39, 43, 44, 46, 53, 54, 59, 60, 63, 75, 85, 88, 90, 97–102, 120, 122–124, 140, 148, 150, 151, 154–157, 159–161, 167, 169, 171–173, 215, 218, 219, 220, 222, 224–226, 229, 230, 235, 236]; physical restraint in mental health settings (n = 30) [23, 32, 40, 41, 48–51, 56, 73, 80, 82, 83, 91, 93, 99, 106, 112, 113, 118, 120, 149, 163, 165, 223, 234, 238, 240, 244, 253]; physical or verbal abuse and/or restraint in elderly residential care settings (n = 44) [17, 28, 29, 36, 42, 45, 47, 57, 58, 62, 65–70, 81, 92, 95, 96, 98, 103, 104, 107–111, 115–117, 119, 126, 132, 134, 135, 189, 164, 168, 214, 228, 232, 241, 243], verbal and/or physical abuse in other settings (n = 35) [33–35, 37, 52, 55, 61, 64, 71, 72, 76–78, 86, 89, 94, 113, 121, 128, 131, 136–138, 143–146, 152, 153, 158, 162, 116, 227, 239, 242]; and physical restraint only in other health care settings (n = 21) [21, 25, 74, 79, 84, 87, 105, 125, 127, 130, 133, 141, 142, 147, 166, 170, 215, 231, 233, 237, 244].

Table 2 provides examples of the theories included for each of these categories.

**Table 2:** Examples of explicit or implicit theories in included papers, by mistreatment type/ setting.

Category	Maternity (n = 58)	Other healthcare settings, not physical restraint (n = 35)*	Physical restraint, not mental health or elderly (n = 21)#	Physical abuse or restraint–elderly (n = 44)	Physical restraint–mental health (n = 30)
***Explicit theories***	Conceptual (structural framework for disrespectful care [53, 54, 173]; Obstetric violence (theory) [157, 215, 218, 222, 230, 225, 226, 229], Overmedicalisation of childbirth [53,75, 217, 218, 230] Gender inequalities (theory developed using USAID Gender Analysis Framework [43] Gender theory [97]; (structural) gender inequality [161, 172, 173];, Gender based violence [75, 140]; Patriarchy [75] Feminist critiques [219]Oppressed groups theory [54], Stigma [140, 151, 172], Moral evaluation of patients[123] Intersectionality [97], Normalisation of violence [50, 217, 218, 224, 226, 235] Foucault (Theory of Inscription/power) [88, 225], Jacobson’s taxonomy of dignity [88], Learned helplessness [88]. Mental models, ‘automaticity’, cognitive availability/scarcity [159], communication theory [54] Authoritative knowledge [160] Cultural health capital [157], Socio-ecological theory [236] Social movements theory [97],	Socioecological/ecological theory [27, 121, 128], positioning [162], medicalisation [113] (medical model of disability), Theory of reasoned action [138]	The theory of planned behaviour [142, 170] Normalisation of restraint [231, 233] Human rights [231], Iceberg theory [213] Risk aversion [242] Labelling theory (victim-blaming) [237]	Stereotyping (ageism [228], normalisation of stereotypes) [234], Racial disparities [66], moral geographies [29], The theory of reasoned actio*n*/planned behaviour [42, 241] Complexity theory [28] open systems theory [68], Socioecological model/theory [216, 228]	(Response to) discriminative stimulus control (behaviour towards staff) & motivating operations (attention-seeking) [129]–relating to the behaviour of the patient, Stigma[56, 234] Trauma informed care [253] Salutogenesis (low Sense of Coherence) [253], Social learning theory [253] Behavioural change theory [240]
***Implicit theories***	Gender inequalities, Gender-based violence, Structural inequalities, Racism, Class inequality, Intersectionality, rurality normalisation/ trivialisation of abuse (personal, organisational, societal), power dynamics, social/cultural norms, behavioural change theory, pathologisation, supervaluation of technology, risk aversion, health industrialisation, behavioural change theory, socio-ecological theory	Institutional, situational and patient characteristics, discrimination, trauma-informed, behavioural change theory	Balance between ‘safety’ and humane care;’safety/risk’ conceptualisations, Institutional norms, risk minimisation (patient safety), labelling theory, depersonalisation and dehumanisation (learning difficulty) behavioural change theory, socio-ecological theory	Risk minimisation/ avoidance—individual level patient safety, staff safety; organisational level–avoiding litigation risk), paternalism (elderly subjects of paternalist control), objectivisation, system failure, dehumanisation, rights violations, behavioural change theory, socio-ecological theory	Patient autonomy, trauma response, organizational culture, risk minimisation/avoidance (patient safety, staff safety, safety vs humanisation), gender inequalities, organisational culture (staffing/resources), behavioural norms (staff knowledge, nurse attitudes), behavioural change theory, socio-ecological theory

*mostly elderly/learning disabilities

^#^mostly surgical or acute care

The following section expands on the theories listed in Table 2.

## Maternity studies (n = 58)

About half of the maternity papers mentioned explicit theories. These included **gender inequalities** (theory developed using USAID Gender Analysis Framework [43]; and feminist critique [219], **gender theory** [97]; **(structural) gender inequality** [161, 172]; **oppressed groups theory** [54] in relation to hospital hierarchies; **gender based violence**[75, 140]; **overmedicalisation** of childbirth [53, 75, 117, 218, 230], and associated **patriarchy** [75]; **cultural health capital** [157]; **stigma** [140, 151, 172] and **moral evaluation** of patients [123]; Foucault’s (1979) **theory of Inscription** [88], in which the risk of being judged “abnormal” by others is internalized, making powerful norms work from within, and Foucaults theories of power/resistance to power [225]; and Jacobson’s **taxonomy of dignity** [88], and **learned helplessness** [88]. One paper [159] explored behavioural concepts of **mental models**, and of **‘automaticity’** (based on the concept of **cognitive availability/scarcity**, in which a hegemonous focus on death avoidance left no space for consideration of other aspects of good quality maternity care. Another [160] was framed by the notion of **authoritative knowledge**, in which the knowledge and beliefs of the most powerful group frame what is acceptable in terms of actions. One paper explicitly cited the **socio-ecological theory** [236].

Implicit theories used in the maternity studies tend to focus on **gender inequalities** [43, 160], internalised, organisational and social **norms around general acceptability of mistreatment** [22, 39, 46, 160, 217, 218, 224, 226, 235] (for example, in the case of the ‘difficult’ patient, or where the safety of the baby was seen to be at risk) and social/cultural norms about the acceptability and expectation of violence against women (**gender-based violence**) [22, 39, 75, 157, 173], **control of deviation** from perceived gender/role or stereotype [43], and **stigma and shaming** around sex (as dirty or sinful) [24, 46, 157, 159]. **Vertical transmission of mistreatment** was captured in theoretical assumptions that gender inequalities and consequent mistreatment experienced by female staff (including limited access to resources and opportunities) could lead to frustration and burnout and consequently blunted compassionate relating, resulting in mistreatment of women during childbirth and labour.

Some of the theories capture how **intersectionality** compounds inequalities in both female staff and service users, and in hierarchies within gender (related to class, ethnicity or marital status). From this perspective, assertion of power differentials (from institutionally oppressed health care professionals to socially oppressed service users) results in **social sanctioning**, and punishment of service users for not following the institutional rules, or for non-payment of fees, as this is a domain over which the staff have control, and the service users do not. Social sanctioning includes restriction of birth companionship to avoid anticipated resistance to rules if the woman has a companion with her, or to minimise external observation of mistreatment.

The relative lack of power of female staff as compared to equivalent male staff is proposed as a reason for finding evidence of greater mistreatment by female midwives/nurses than male health workers in equivalent roles. **Power based theories** also argue that the struggle to assert the professional status of midwifery/nursing results in preference for **over-medicalisation** by staff in some contexts, and of strong **local norms** in which service users are often conceptualised as inferior to staff, socially and morally. In a nuanced take on **gender based violence theories**, where the gender of the person inflicting abuse was explicitly examined, it was more often attributed to female rather than male staff.

Poorly resourced facilities (i.e. resources, workload, skilled staff) and institutional infrastructure (lack of supervision) are repeatedly mentioned across papers, as are **institutional norms about mistreatment** (e.g. as acceptable practice).

In the index review, there are some differences in emphasis across the dataset. Within African countries, some report on gender based violence (Ethiopia [31, 38, 155], Kenya [22]) or sexual shame (Nigeria) [46]). In others, this is focused on other forms of discrimination, such as social class. In contrast, for India and Pakistan, analyses were more likely to be undertaken through the lens of social class discrimination [44, 102, 154]. Studies undertaken in Arabic counties cited predominately cultural/institutional hierarchies/pressures [24, 30, 100]. However, these differences were not as evident in the papers generated by the updated review.

Two conceptual frameworks were cited: The **Conceptual Framework for Disrespectful Care in Maternity** which includes drivers of mistreatment and abuse [53, 54], and the **Framework of Obstetric Violence**, which includes both facilitators of such behaviour (social factors, harmful cultural practices, systemic barriers, historic normalisation), and solutions for change [157]. The general concept of obstetric violence featured in a number of studies [eg, 216, 218, 222, 225, 226, 229, 230].

### Physical and verbal abuse in non-maternity studies, other than physical restraint (n = 35)

A few studies in this category include patients in general wards and emergency departments [33, 35, 71, 72, 131, 158], adults with learning difficulties [64, 113], and psychiatric inpatients [86]. However, most identify risk factors associated with mistreatment of elderly persons in residential care [37, 52, 55, 61, 76–78, 89, 94, 121, 128, 136–138, 143–146, 152, 153, 162], highlighting interactions between institutional factors and staff and patient characteristics, rather than proposing theories to explain this, with the exception of two papers. Moore [136] discusses a range of social psychology theories and explores findings using a socioecological framework, proposing that Henri Tajfel’s theory on the **social psychology of intergroup relations** is a useful theoretical model to explain abuse of elderly people in a care home. Natan [138] uses the theoretical model for predicting causes of maltreatment of elderly residents developed by Pillemer [144]. This includes three components of the work environment; patient traits; and the **Theory of Reasoned Action** developed by Ajzen & Fishbein [256].

One of the papers also mentions exogenous factors [144] such as bed shortage based on local supply and unemployment rates. Another paper [162] on elderly care used the **positioning theory** in which interactions are conceptualised as being based on individuals taking certain ‘positions’: clusters of rights and duties to act in certain ways and impose particular meanings, which enable or prohibit access to certain storylines. The **medical model of disability** is mentioned in a paper about care of women with learning disabilities [113].

More generally, most papers in this area argue that relevant **situational factors**, including staff burnout, patient-staff conflict, lack of knowledge and/or relational factors impact on mistreatment [61, 78, 121, 128, 144–146]. **Victim blaming** was used as a justification for mistreatment in a number of studies, on the basis that elderly residents were aggressive and abusive, and therefore required a robust physical or verbally response to regain control over the individual and/or the situation [37, 76, 77, 138]. **Gender issues** were also raised, in that female older adults were noted to be most likely to experience physical abuse [37, 131, 138, 152]. In parallel with the evidence on mistreatment in maternity care when women do not have labour and birth companionship, **social isolation** was noted to be a relevant factor, particularly for older adults who did not have relatives and friends as protectors, so were deemed to be most likely to experience abuse [37, 144].

A conceptual review [257] was referenced in one of the included papers. This was not located in the search for the current study, as it did not include the key words. This review identifies a typology of risk factors for maltreatment, including institutional factors, carer and patient characteristics.

#### Physical restraint, not in elderly populations or in mental health (n-21)

An explicit theory was only mentioned in a few papers in this category. Both Via-Clavero [170] and Perez [142] used **the theory of planned behaviour** to explain physical restraint use and how to reduce it in intensive care unit settings. The authors discuss the influence of workload pressures on nurses’ intention to use physical restraint, and subjective norms and controlling factors that contribute to this, in line with Ajzans theory [256]. Acevedo-Nuevo uses the Iceberg theory noting: ‘*According to this theory*, *the visible part of the iceberg represents PR use*, *but lower down*, *an intricate network of interrelated elements is at work; here*, *all health care actors exert an influence on the main actors in PR use (i*.*e*., *the nurses)* (p3) [213]. Social and organisational normalisation/ of restraint is explicitly referred to in two studies (p78) [231, 233]. Smithard notes: *For many the use has become institutionalized*, *with accepted practice hard to* change [233].

Staff and external organisational risk aversion [242] and labelling theory (related to victim blaming) [237] also feature in this group of studies.

Implicitly, notions of **institutional norms to protect patient safety** are particularly evident in the studies in the intensive/critical care setting, either due to the nature of the procedure or patients’ functional ability, centred around preventing interference with tubes and/or to prevent extubation [105, 130, 133, 142]. Lach notes that *Prevention of falls is a primary reason for restraint use on medical–surgical units*, *whereas preventing removal of medical devices and confusion are primary reasons in critical care settings*.[125] In many of these analyses, the point is made that there is evidence that restraint use in these settings does not in fact lead to decreased danger for patients, in direct contrast to nurses beliefs.

In three studies [105, 142, 242], the concept of **risk minimisation** is discussed in more depth along with the implications of a **blame culture** in which nurses have to take on responsibility for decision making in relation to restraint use in critical/acute care. The argument is that this leads to unnecessary or over-use of restraint, for fear of litigation, or of blame within the organisational structure, in line with some of the self-protective theories in maternity care noted above. In this case, the personal consequences of making a decision that is not in line with the risk minimisation focus of the organisation outweighs the risk to the patient, or the professional values of the nurse, leading to a sense of lack of mutual support, and therefore increasing the likelihood of using physical restraint.

### Physical abuse or restraint in the elderly (n = 44)

Where explicit theories occurred in this set of papers, they included the use of **complexity theory** [28], and the **theory of self-organisation** (practice that emerges based on the interconnectivity between people and how they relate to each other), and related notions of **open systems theory** [68], in which organisations are influenced by the context and environment they operate in. Approaches also included **moral geographies** [29] in which moral norms for a particular group are calibrated in relation to the physical proximity of people to each other, and across different geographical spaces, such as private versus public domains. **Behavioural change theories** featured in two papers [42, 241]. Theories of **racism** [66] were also used, where Black residents were more likely to be abused than White residents. **Stereotyping and normalisation of stereotypes**, specifically negative beliefs about, and attitudes towards, the social value and status of the elderly were identified [228, 232]. Some authors developed their own frameworks of interactions between factors such as organisational, resident functional capacities/behaviour, personal and psychosocial drivers, and physical environment, to examine associations with physical restraint [164, 132]. For two authors, these levels of action were formally captured in the **socio-ecological model** [116, 228].

Where theory was implicit, the predominant explanation of the use of physical restraint in elderly population is for patient safety (e.g. to protect patients from falls and prevent injury from wandering). Underlying this ideology of protection of elderly residents is a belief system within institutions that patients are frail and have diminished responsibility and lack autonomy, in parallel with staff fear of litigation or blame by peers and superiors when accidents and injuries do occur. Thus, as in other sections of this review, physical pressure or restraint was used in the belief that it offered protection from adverse physical outcomes for the elderly, and was prioritised over dignity and autonomy, even though this was recognised as dehumanising by some staff.

The consequent disconnect between professional values of care and compassion and the action of enacting physical restraint resulted in moral distress for some staff. Indeed, some papers discussed the need for balance in maintaining physical and psychological integrity, presenting nurses with an ethical dilemma with the use of physical restraints. In this case, underpinning decision-making was the ideology of **nursing as a moral practice** (particularly Goethals [95]). This is associated with ‘doing good’ with protecting patients from physical harm and/or maintaining the psychological integrity/safety of other elderly patients.

One review highlighted **social and cultural norms** as an important influencer of physical restraint by nursing staff, highlighting differences in restraint use between the Netherlands, Germany and Switzerland [114].

### Physical restraint in those with mental health issues (n = 30)

Two papers in this group focused on **stigma** against those with mental health issues [56, 234], Trauma informed care, social learning theory, salutogenesis (ie, the impact of a low Sense of Coherence) all featured in a review undertaken by Perers [253], Other studies related to this topic were more focused on behavioural and psychological theories, including how staff response is stimulated by patient actions [129] and behavioural change theory [240].

Implicit theories were linked to the dilemma between risk minimization and humanization. Organisational barriers to environments that promote on-going support of, and knowledge development in, local practitioners included low levels of staffing and resources. Gender inequalities also featured in some of the discussions, in terms of assumptions about how men and women ‘typically‘ provoke violence, or not.

Another emergent topic in this category was the need for staff to optimize a sense of safety and security within a mental health setting, and consequent **risk aversion**. The argument is that when there is increased staff exposure to resident or patient aggression, there may also be an increase in the use of restraints, due to a desire for **self-preservation** by staff [41]. In some cases, nurses acknowledged that while it is not a first line treatment, in situations where no other alternatives exist, restraint is a ‘necessary evil’ [93].

Although not mentioned as a primary theory, **patient-provider communication** was found to be an implicit theme in a number of studies in which patients/residents reported that restraint was unnecessarily overused, linking with the **over-medicalisation** theories in other sections above [56]. In another study, discussion with patients uncovered a desire for communication and collaboration to establish restraint-use alternatives [91].

### Papers reporting non-maternity intervention studies (n = 53)

The full characteristics of these papers are given in S4 and S5 Tables. The summary characteristics are provided in Table 3.

**Table 3:** Summary of characteristics of included primary non maternity intervention studies.

	RCT/CCT	Before & After
***Study Setting***		
In facilities/acute care		3
Care home	10	2
Children’s care home		2
Hospital		1
ICU		11
Psychiatric hospital	5	15
Residential camp		1
***Country***		
Canada		1
China	1	2
Denmark	1	
Finland	1	
Germany	3	
Iran		1
Netherlands	3	
New Zealand		1
Norway	2	
Spain		
Sweden	1	
Taiwan		2
UK	1	2
USA	1	23
NR/various	1	3
***Intervention based on theory***		
Yes	7	11
No	6	12
Unclear	2	12
***Intervention type***		
Education/training	7	5
Mindfulness	1	2
Multicomponent	6	27

They included 15 randomised controlled trials (RCTs) and controlled clinical trials (CCTs) [174, 176, 182, 187, 191–193, 195, 199, 200, 203, 208, 209, 258] and 35 prospective before and after studies (pre- and post-design) [165, 177–181, 183–186, 188–190, 194, 197, 198, 202–210, 243, 244, 246–251, 253, 254],. Three were reviews [242, 245, 252]. Most studies were set in psychiatric hospitals (n = 20) [176, 177, 181–183, 185–189, 198, 200–202, 206, 217, 243, 244, 247, 255] or elderly care homes (n = 12) [174, 191–193, 195, 196, 199, 203–205, 207, 209]. Other settings included acute and other hospitals (n = 4) [118, 178–180], intensive care units (ICU) or emergency units (n = 11) [175, 196, 197, 212, 246, 248–250, 251, 253, 254], children’s care homes (n = 2) [184, 190] and a residential camp (n = 1) [210]. All the studies were conducted in MICs or HICs, particularly the USA (n = 24) [178, 181, 182, 184–186, 188, 190, 194, 198, 201, 202, 206, 207, 210, 201, 243, 244, 247–249, 250, 251, 253].

In 18 studies the intervention was based on an underlying theory (7 RCTs/CCTs and 11 before and after studies) [175, 159, 161, 162, 164–167, 169, 172–176, 180, 181, 224, 225]. The interventions were predominantly either multicomponent (n = 33) [175, 176, 177, 182–190, 195–198, 200, 202, 206, 208, 209, 211, 243, 244, 246–250, 251, 253–255] or educational/training (n = 12) [175, 178, 199, 191–194, 199, 207, 208, 210, 212] They were all compared with usual care (including baseline standard practice for before and after studies). The outcomes assessed focused on different measures of restraint, including prevalence, reduction in numbers of restraint episodes, or staffs’ attitudes towards and perceptions of restraint.

### Fidelity, acceptability and sustainability

This was assessed for the studies in the updated review. In general, these domains were not well reported. Only one paper cited a protocol [255] but this could not be found on the Chinese Trials Registry, and it isn’t clear if the study followed the protocol that was registered.

Chen [246] and Dixon [247] both reported that their tools seemed to be acceptable to staff. Hevener [249] undertook a formal staff audit, and noted that, while the majority of respondents found the intervention tool (the Restraint Decision Wheel) to be useful in principle, they still preferred to use their own judgement in deciding when to restrain a service user.

Sustainability was implied for six studies where time series data were reported [243, 244, 249, 250, 251, 253], all generally showing maintenance of changes over time.

### Effectiveness

For individual studies, comparison of the different interventions with usual care tended to demonstrate positive change in the primary outcomes measured in the intervention arms, or the ‘after’ phase. However, only 14 studies provided point estimates and measures of variability to enable meta-analysis, and these were all measures of impact on physical restraint [174, 183, 187–188, 191, 193, 194, 196–198, 203, 204, 248, 253]. The characteristics of these studies are provided in Table 4.

**Table 4:** Characteristics of studies included in the meta-analysis.

Study	Setting	Design	Primary outcome	Outcome in current analysis	Intervention	Underlying theory	Comparator
Abraham[174] 2019 Germany	120 elderly nursing homes in 4 regions	Cluster RCT	Physical restraint prevalence	Mean prevalence (%) of any physical restraint	1Two versions of a guideline, & a multicomponent educational intervention 2Concise version of 1	No explicit theory mentioned (guidelines developed through consultations with experts)–targeting nurses’ attitudes and organisational culture, though Kopke referenced, which implies the theory of planned behaviour	Optimized usual care (supportive materials only)
Bowers [183] 2006 UK	Two acute psychiatric wards	B&A	Multiple conflict and containment outcomes	Restrained mean per shift	Two ‘City Nurses’ were employed to work with ward staff, to operationalise the working model	No explicit theory mentioned–working model involving positive appreciation, emotional regulation and effective structure–based on 6 core components: 1) the psychiatric philosophy of staff; 2) their moral commitments, 3) their use of cognitive-emotional self-management methods, 4) their technical mastery in interpersonal skills, 5) team work skills, 6) organisational support.	Before intervention
Duxbury [187] 2018 UK	14 acute psychiatric wards in 7 hospitals	Individual RCT	Physical restraint incidents	Restraint event rates per 1000 bed-days	REsTRAIN YOURSELF intervention: (UK) modified version of ’Six Core Strategies’ (US) multimodal approach to reduce restraint—prevention & trauma informed principles	No explicit theory mentioned–—underpinned by principles of prevention and trauma informed care—based on six core strategies–leaders for organisational change, the use of data to inform practice, workforce development, person-centred tools, service user roles within patient settings and debriefing techniques.	Usual care
Godfrey [188] 2014 USA	1 psychiatric ward	B&A	Mechanical restraint incidents	Mechanical restraint use (daily incidence rate)	2 strategies: (1) staff training in de-escalation techniques (2) policy change for the use of mechanical restraint	No explicit theory mentioned–based on recovery oriented trauma-informed care	Before intervention
Huizing [191] 2006 Netherlands	5 psycho-geriatric nursing homes	Cluster RCT	Restraint prevalence and intensity	Mean % of residents observed being physically restrained at any time during a 24hrs period	An educational programme for selected staff to reduce restraint use, and a consultation with a nurse specialist	No explicit theory mentioned	Usual care
Huizing [192] 2009 Netherlands	5 psycho-geriatric nursing homes	Cluster RCT	Restrained status, intensity, use of multiple restraints	No. of times resident was physically restrained in 24hrs	An educational programme for selected staff to reduce restraint use, and a consultation with a nurse specialist	No explicit theory mentioned.	Usual care
Johnson [194] 2016 USA	1 trauma intensive care unit	B&A	Restraint use	Mean Restraints per 1000 patient days	Power point review of non-pharmacological interventions and alternative devices with hands on demonstration with the devices were provided in the TICU on both day/evening shift. The programme was designed to enable staff to tailor appropriate non-pharmacological devices to the individual patient	No explicit theory mentioned	Before intervention
Kopke [196] 2012 Germany	36 nursing home clusters	Cluster RCT	% of residents with physical restraint	% of residents with physical restraint	A multidisciplinary approach designed to address attitudes, subjective norms and perceived behavioural control; evidence-based guideline, information programs, endorsement of nursing home leaders, and support materials	Theory of planned behaviour	Standard information delivered in previously developed brochures
Lin [197] 2018 Taiwan	3 neurological intensive care units	B&A	Incidence rate of physical restraint	Physical restraint hours	Standardised PR reduction program developed by a multidisciplinary team	No explicit theory mentioned	Before intervention
McCue [198] 2004 USA	1 hospital: psychiatric inpatient service	B&A	Restraint use	Number of restraints per 1000 patients days	6 interventions primarily involving changing staff behaviour: better identification of restraint, stress/anger management group for patients, staff training on crisis intervention, development of a crisis response team, daily review of restraints, a staff incentive system	No explicit theory mentioned	Before intervention
Singh [203] 2016a USA	5 care homes for those with intellectual disabilities	Individual RCT	Reduction in psychological stress, physical restraints and restraint medications	Average use of physical restraint per week	7-day Mindfulness-Based Positive Behaviour Support (MBPBS) for caregivers	No explicit theory mentioned.	Training as usual
Singh [204] 2016b USA	1 care home for those with intellectual and developmental disabilities	B&A	Reduction in physical restraints, staff injury, peer injury, staff turnover	Weekly frequency of staff use of physical restraints	7-day Mindfulness-Based Positive Behaviour Support (MBPBS) for caregivers	No explicit theory mentioned.	Before intervention
Hall [248] 2018 USA	1 intensive care unit	B&A	Proportion of patients restrained	Restrained patients per patient day	Daily audit and review of restraint information; Customized restraint management education, including case studies; standardising and monitoring restraint audit tool	No explicit theory	Before intervention
Shields [253] 2021 USA	1 ICU unit	B&A	Restraint use	Mean % with restraints	12 different components for the intervention package	No explicit theory mentioned.	Before intervention

Most of the meta-analysed papers do not explicitly mention underlying theory. Some report consultation work to develop guidelines with experts based on the evidence base, but do not mention specifics about the evidence base. Where explicit theory is mentioned, and where theory was implicit, the general approach seems to have been some version of the theory of planned behaviour [262]. Thirteen of the interventions [174, 183, 187, 188, 191, 193, 194, 196–198, 203, 204] were primarily focused on training in alternative strategies so appear to be based on the hypothesis that excessive use of restraint is related to a lack of knowledge about alternatives. Two papers (same study team) [203, 204] used mindfulness methods, implying a hypothesis that stress/distress in staff or patients could be a trigger for unnecessary physical restraint

### Risk of Bias

Risk of bias was evaluated for all of the studies included in the meta-analysis (See Table 5). Full risk of bias details are in S6 Table.

**Table 5:** Summary risk of bias for studies included in the meta-analysis.

**RCT (Parallel)**	
Singh 2016a [203]	Some concerns
**RCT (Cluster)**	
Abraham 2019 [174]	Some concerns
Huizing 2006 [191]	Some concerns
Huizing 2009 [192]	Some concerns
Kopke 2012 [196]	Some concerns
**Before & After**	
Bowers 2006 [183]	Fair
Duxbury 2019 [187]	Fair
Godfrey 2014 [188]	Good
Hall 2018 [248]	Fair
Johnson 2016 [194]	Fair
Lin 2018 [197]	Good
McCue 2004 [198]	Poor
Shields 2021 [254]	Fair
Singh 2016b [204]	Fair

The assessment revealed some concerns with twelve of the fourteen studies (and all of the RCTs) [174, 183, 187, 191, 193, 194, 196, 198, 203, 204]. The funnel plots also suggest some publication bias, so the results of the meta-analysis should be treated with caution.

### Findings

The 14 studies made 15 comparisons. Although two studies presented two comparisons, only one comparison could be included as the other reported no events [188].

**Fig 3:**
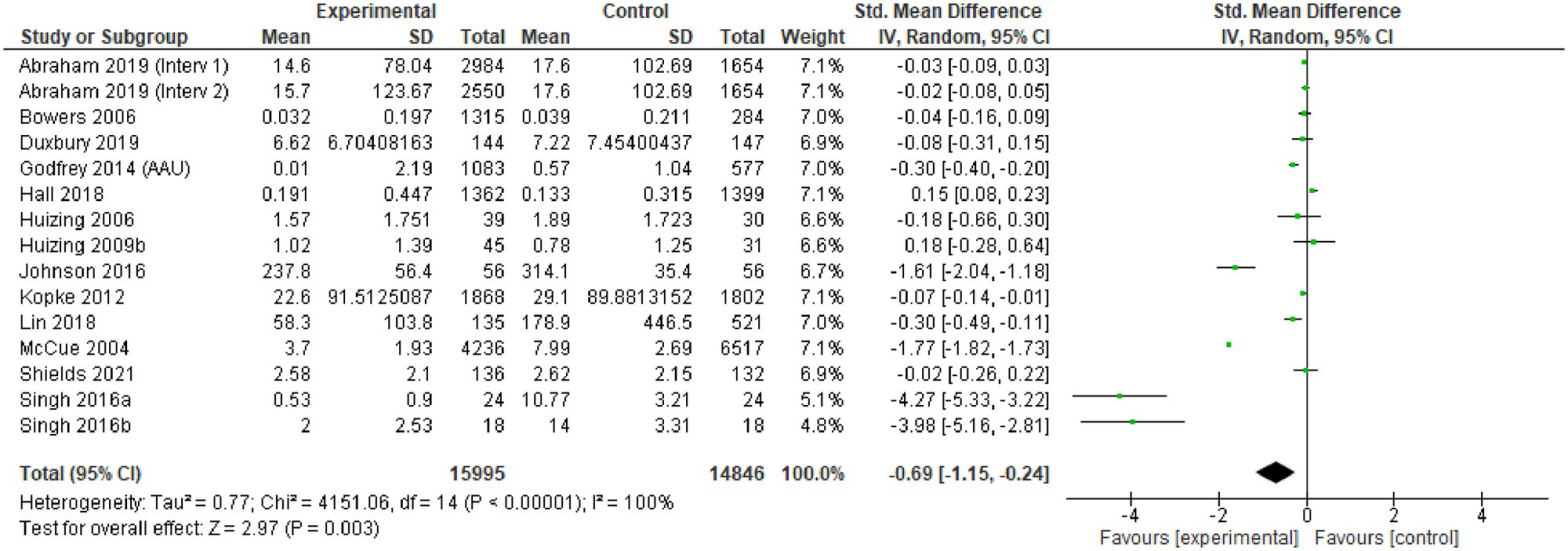
Effectiveness of interventions on the use of restraint: All included studies.

The pooled outcome showed a statistically significant beneficial effect in reducing restraint following the different interventions compared to usual care (SMD -0.69 (95% CI: -1.15; -0.24) (Fig 3).

**Fig 4:**
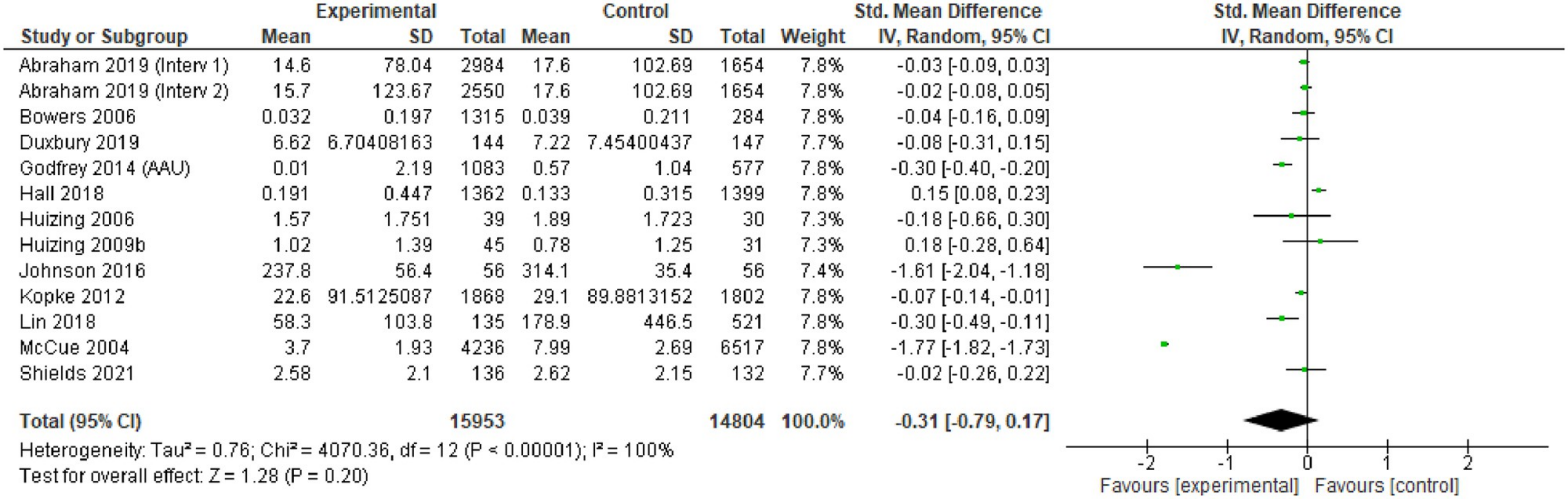
Effectiveness of interventions on the use of restraint, excluding outliers.

Two studies, which assessed the effects of mindfulness-based positive behaviour support, found markedly strong effects[203, 204]. When these studies were excluded through a sensitivity analysis, the pooled effect was reduced, becoming marginally statistically insignificant (SMD -0.31 (95% CI: -0.79; 0.17)) (Fig 4).

**Fig 5:**
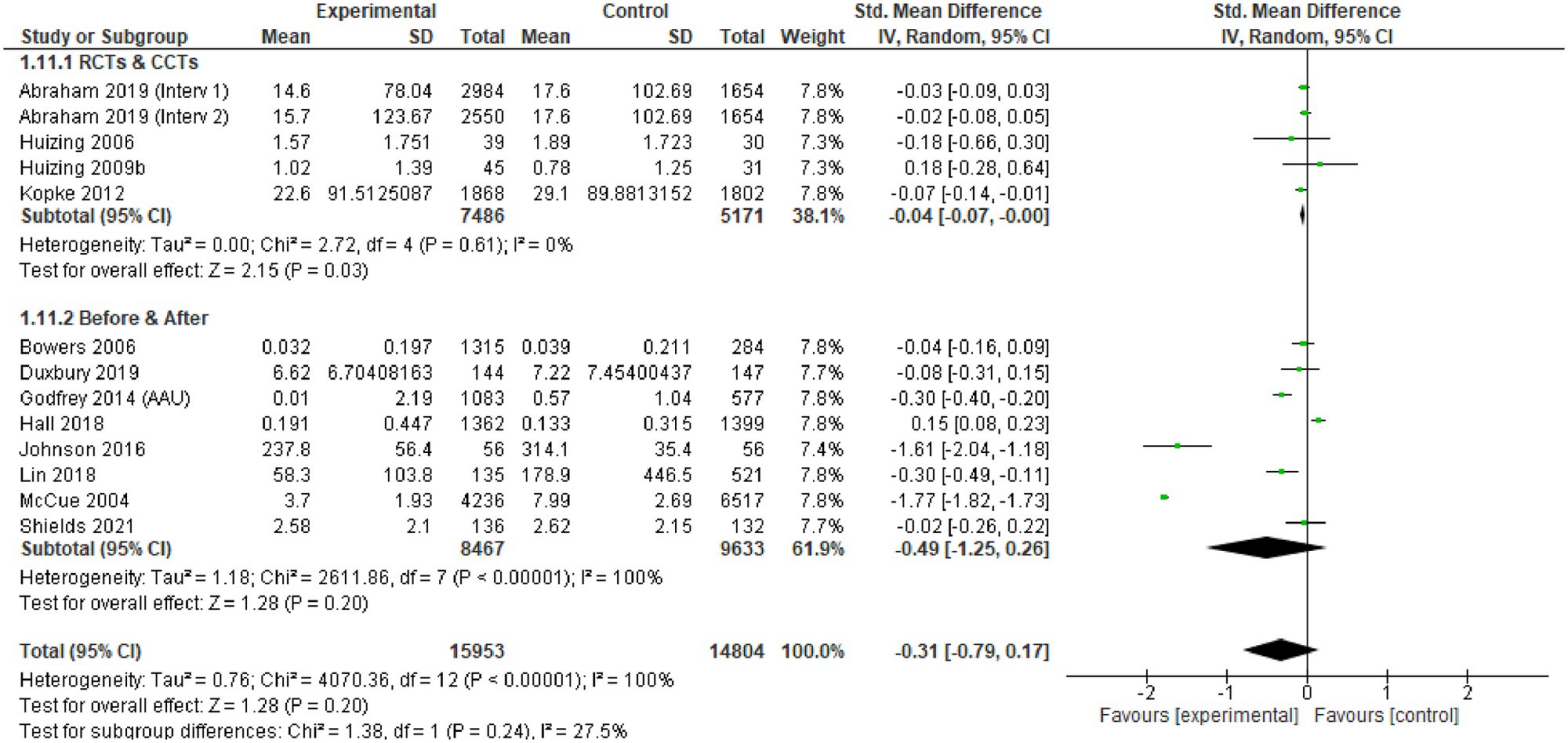
Effectiveness of interventions on the use of restraint by study design, excluding outliers.

**Fig 6:**
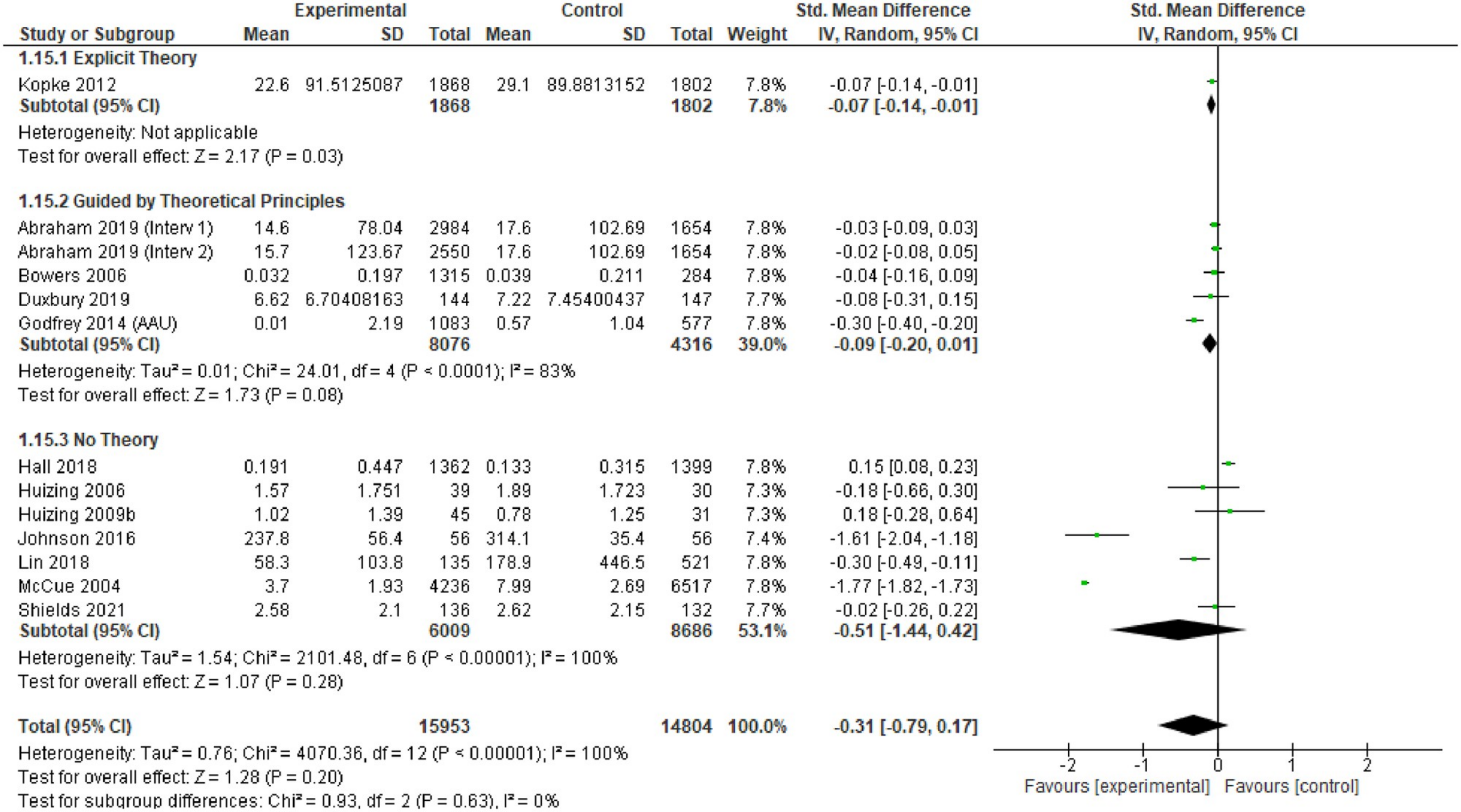
Effectiveness of interventions with an underpinning theory on the use of restraint, excluding outliers.

**Fig 7:**
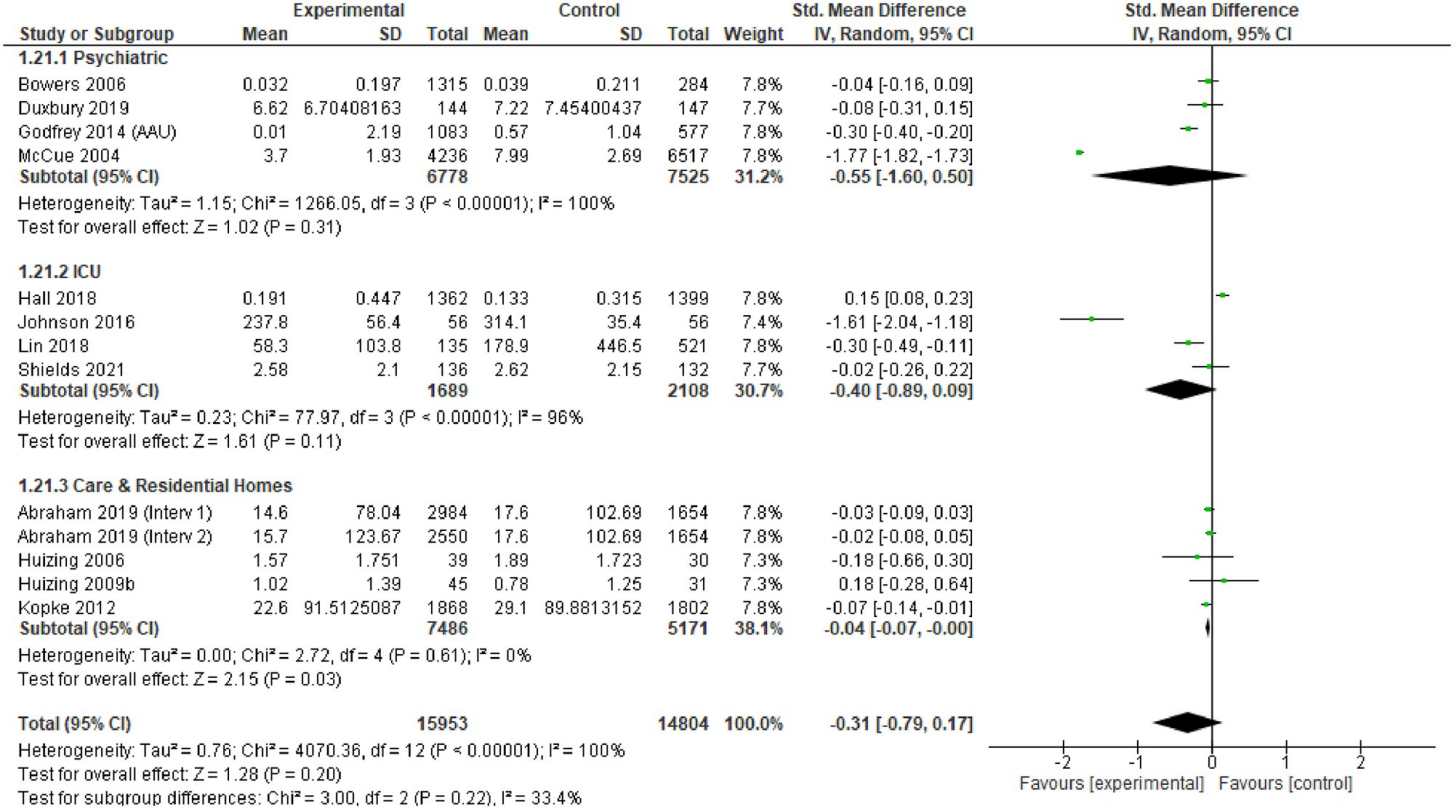
Effectiveness of interventions on the use of restraint by setting of care, excluding outliers.

Sub-group analyses were largely inconclusive, reflecting uncertainties in the evidence base. RCTs and CCTs, (providing more robust evidence), reported a small statistically significant benefit from the different interventions when compared with before and after studies (SMD -0.04 (95% CI: -0.07; -0.00)) (Fig 5). A similar small benefit was found from interventions based on explicit theory, but this only comprised one study [177] (SMD -0.07 (95% CI: -0.14; -0.01); from studies with explicit or implicit underpinning theory (4 studies, 5 comparisons); SMD -0.09 (95% CI: -0.20–0.01) (Fig 6); or for interventions used in care and residential homes [155, 172, 174, 177] (SMD -0.04 (95% CI: -0.07; -0.00)) (Fig 7).

**Fig 8:**
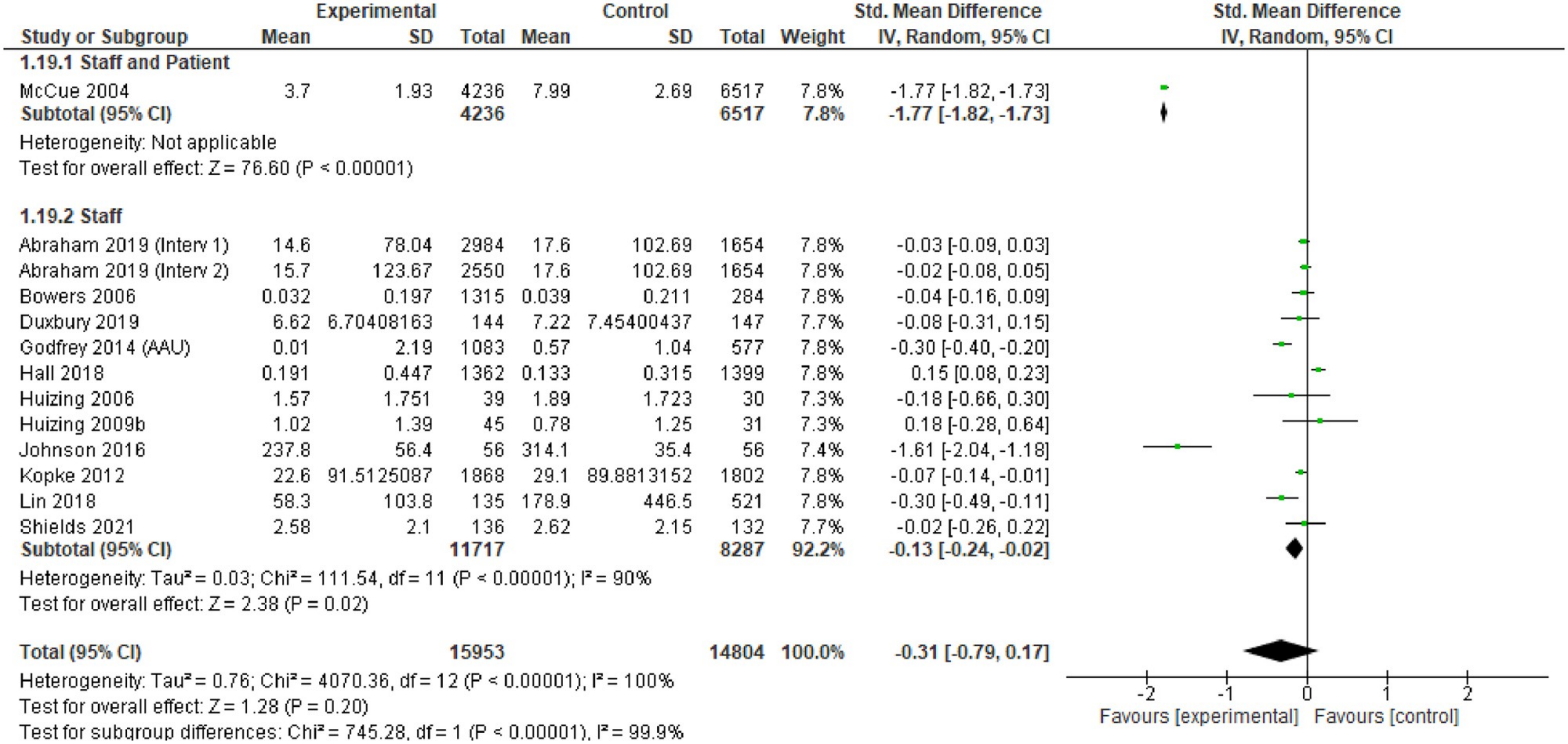
Effectiveness of interventions on the use of restraint by those receiving the intervention, excluding outliers.

**Fig 9:**
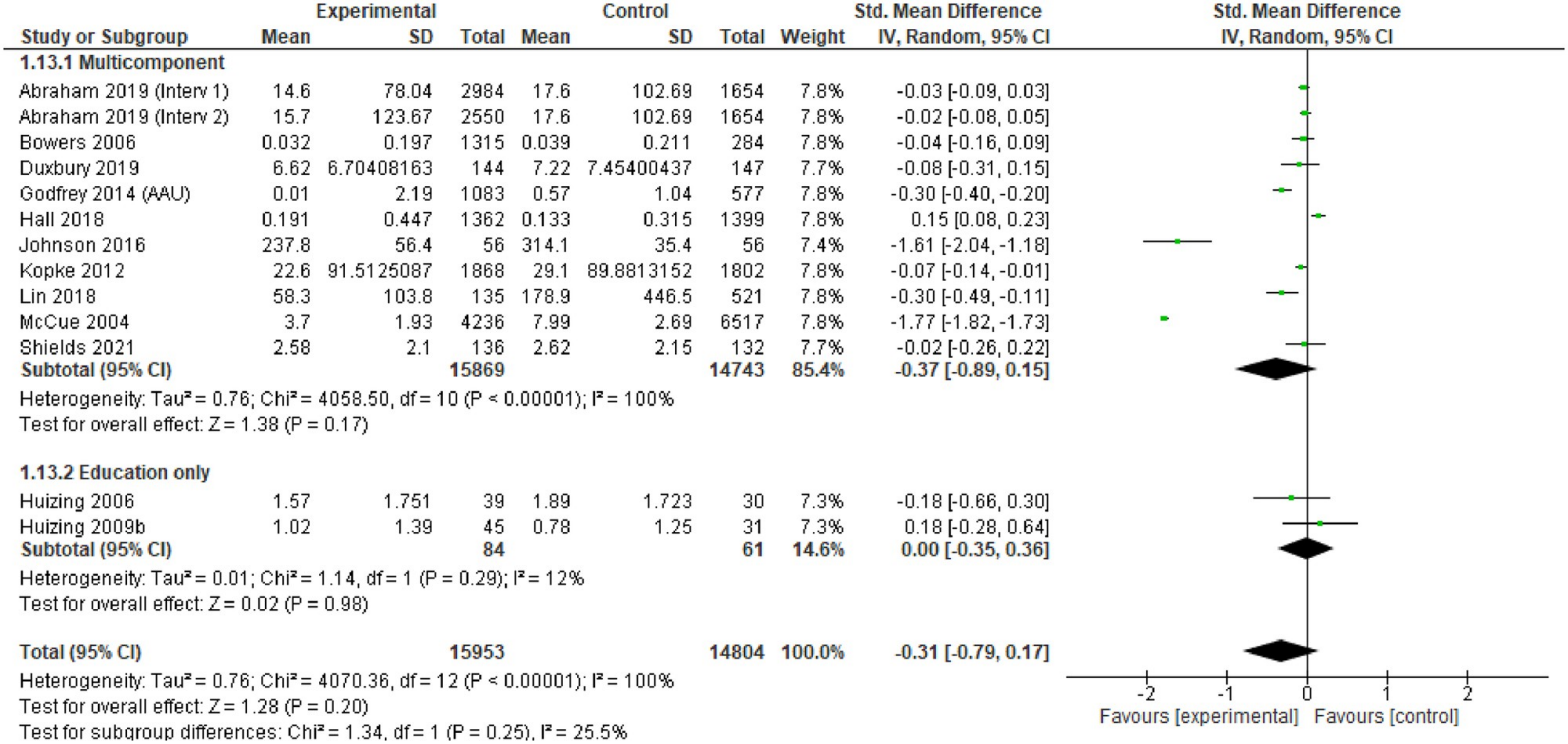
Effectiveness of interventions on the use of restraint by intervention type, excluding outliers.

An intervention that involved both staff and patients had a greater benefit than those focused on staff only [179] (SMD -1.77 (95% CI: -1.82; -1.73)) (Fig 8). Multicomponent interventions seemed to have a larger effect than those based on education alone, but this did not reach statistical significance (SMD -0.37 (95% CI: -0.89–0.15) (Fig 9,. The influence of a country’s income could not be assessed as all studies that were suitable for meta-analysis were undertaken in high-income settings.

Heterogeneity affected most of the meta-analyses, despite undertaking sub-group and sensitivity analyses (I^2^ >90%). Only the sub-group analyses that focused on the RCTs and CCTs, on the education only interventions, and on care and residential home settings reduced the heterogeneity to being unimportant. The occurrence of heterogeneity is unsurprising given the diverse evidence base, where participants, interventions, outcome measures, study designs and setting vary considerably.

Importantly only 14 of the 50 non-maternity controlled studies were included in the meta-analyses. This reflects the lack of data reported in the studies, with many not reporting measures of variability (e.g. standard deviations, confidence intervals), or participant numbers, or outcomes for different groups.

## Synthesis

The analysis above indicates that a wide range of implict or explicit theories have been used in studies and papers that address mistreatment, including physical and/or verbal abuse, in health and social care. The theories used tend to focus either on aspects of the health or social care system, or on wider societal and cultural norms, or on the behaviours, norms and attitudes of the individual practitioners working within these systems. Based on these observations, we propose a model that could explain the mechanisms that might trigger and sustain physical and/or verbal abuse (and that therefore could be used for change in future maternity care interventions). The approach is based on an integration of both the social-ecological theory [258, 259] and the theory of planned behaviour [256], to capture both systems and people factors.

We propose that this model, or alternatives that integrate both a systems wide and a human factors approach, might be a useful template for formative research. This could be used to identify mechanisms of effect in settings where disrespectful behavioural norms are proving to be resistant to change, as well as being a vehicle for ensuring that, once identified, any future local intervention can be designed to address and change these negative drivers.

**Fig 10:**
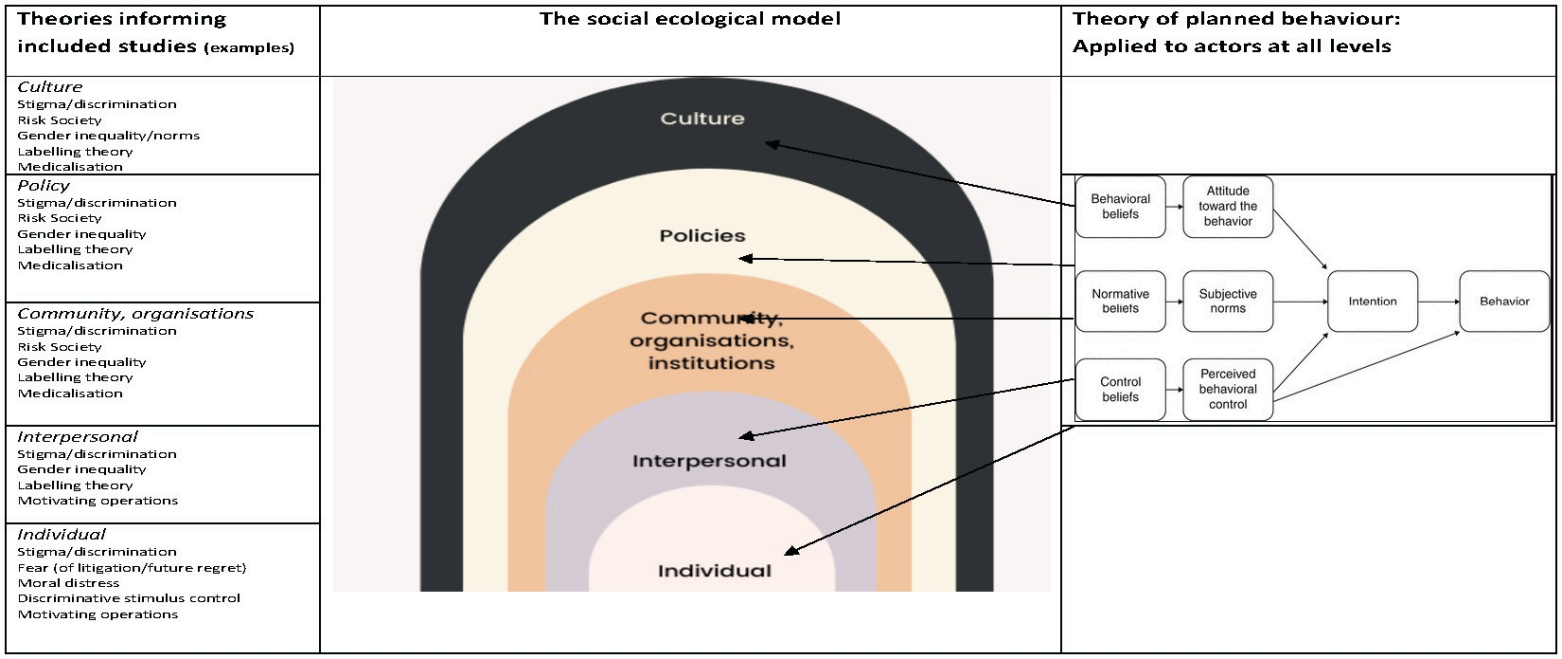
Proposed socio-ecological-behavioural approach linked to example theories from included studies.

There are many variations of the socio-ecological model in current circulation, applied to a wide range of disciplines and problems. What they all have in common is an understanding that human behaviour occurs in nested layers of the social systems in which people operate. The original model was proposed by Bronfenbrenner in 1977 [258] in the context of child development, with his final version being published in 1994 [259]. Fig 10 summarises the levels that are usually included in socio-ecological models (sometimes also categorised into ‘micro-meso-exo-macro’ domains).

The model has an affinity with systems thinking and with complexity theory concepts of inter-connectivity, emergence, self-organisation and self-similarity at different levels of an organism or agency [260]. In other words, systems that are connected across some or all of these levels can tend to express similar characteristics in the component parts at each level. This is important in terms of understanding verbal and physical abuse in maternity care, as it is likely that attitudes, behaviours and norms that are apparent at lower levels of the system are modelled at higher levels of the system. Operating theories located in this review, such as gender norms, stigma and discrimination provide examples of this hypothesis.

Bronfenbrenner himself acknowledged that levels of the system operate in interaction with the people within them. His thinking moved from an emphasis on context towards an increasing recognition of the importance of recognising how far the individual (specifically the developing child, in his work) is in interplay with, and influences, that context. In this reading, the emergence of the cultures and norms, and, therefore the operation of each level of the socio-ecological system can be seen as a function of personal interaction, at least to some extent. This suggests that the analysis of and impacts on human behaviour at the individual/interpersonal level (as many implementation of change strategies are designed to do) misses critical human agency drivers and barriers at the meso-exo-macro level. This might limit the possibility of enacting change effectively, raising the hypothesis that understanding and influencing human behaviour at all levels of the system might be the key to change.

The family of theories that was most often evident in this review (either implicitly or explicitly) was one of the forms of the theory of planned behaviour. First published as the theory of Reasoned Action in 1980 [261] following an earlier paper setting the groundwork [262], the theory has undergone a series of transformations [263] as it has been refined to take into account psychological processes that human beings engage with as they decide whether or not to act in particular ways in specific circumstances. The labelling and composition of the model that results from the theory have varied over time, but the fundamental components relate to (perceived or actual) control over undertaking the behaviour; subjective and social norms (whether people that matter to the individual would approve, and whether the local social norms would support the action); and behavioural attitude (the extent to which performing the behaviour is perceived to be beneficial). The later formulations of the model add a level of intention to each of these three core components.

This iteration of the theory is detailed enough to capture both attitude and intent, while being simple enough to be interpreted in formative research. Implicitly or explicitly, many subsequent change models and theories encompass these concepts of, broadly, what is ‘normal’/supported/enabled in the local context; what is easy for the individual to do; and what gives the individual a sense of benefit.

Despite the apparent utility of both systems and psychological theories in the context of health and social care, they have rarely been synthesised together. While socio-ecological theory is built on human/system interaction at the supra-individual level, this is usually in terms of the person experiencing the system (the child, patient, service user and so on) and not at the level of the people who are creating and sustaining it (staff, managers, policy makers, social influencers, for instance). In 2015 Nilsen identified around 40 frameworks, models and theories used in implementation science [264] most of which either have a primary focus on either systems/ environment, or on human factors. Few recognise that human factors influence all levels of the health and social care system. The assumption seems to be that either ‘the system’ is the issue, with resource limitations or availability and system constraints or freedoms forcing individuals to act in certain ways: or the individuals within the system are the drivers, having freewill to accept or resist institutional and social norms and expectations, including norms of stigma, stereotyping, and the range of theories of discrimination, othering, and exclusion identified in this review.

However, arguably, and as implied by Bronfenbrenner in his later outputs [259], systems are people. Deciding which resources to allocate to ensuring staff are well trained and that services are well-staffed, or that training schools are available, or who has the right to access care, or any of a myriad of other system level barriers and facilitators, are all decisions made by individuals acting on the basis of what they believe to be normal/acceptable to those they value or who have power over them; who make a judgement about what is the most likely (easiest) action or set of actions for a positive outcome; and about the degree of control they have to enact that resource allocation, or to legislate for a certain kind of (non)-discrimination; or to use nudge techniques to create a certain new cultural norm, for instance.

This suggests that interventions that do not take account of **both** organisational, cultural and political pressures, **and** the psychological intentions of actors at all system levels (based on their prior prejudices, attitudes and beliefs) will not be able to enact effective shifts in behaviours across the socio-ecological levels. In terms of respectful care, we have modelled this assumption in Fig 10, using examples of some of the explicit or implicit theories located in the review. This is not an exhaustive exercise–the intent is to show that at least some of the theories identified in the review could be mapped to all levels of the model.

### Mapping the synthesis to maternity care interventions

We only located two controlled intervention studies in maternity care, published (in three papers [14, 15, 265] since our systematic review of the effectiveness of interventions to increase respectful care in 2018 [9]. The Asefa study [14, 15] was before and after, was reported in 2020, and was undertaken in three hospitals in Ethiopia. The intervention was based on a multi-component programme, including training of service providers, putting posters about the right to respectful care in labour rooms, and post-training quality improvement visits. It included measures of physical restraint and of gagging, both of which were reduced by the intervention, though the reduction did not reach statistical significance.

The study undertaken by Mihret [265] was also a before and after study, undertaken in 2020 in one hospital in Ethiopia. It was a multicomponent intervention, focused on both the antenatal and intrapartum period. The authors reported large differences between pre and post study measures, in overall disrespect and abuse, and in physical abuse specifically.

Putting these studies alongside the five studies (six papers) included in the Downe review [266–271] results in seven intervention studies, all undertaken in hospitals in either Ethiopia or Tanzania. Study characteristics and explicit/implicit underlying theories are given in Table 6.

**Table 6:** Characteristics of published studies of respectful care interventions in maternity services. (outcomes that improved with the intervention in bold: associated theory papers in italics).

Author	Design	Focus	Setting	Country	Intervention	Outcome measures^+^	Underlying theory
Abuya 2015 [270] *[172]*	Before/ after	Reduction in disrespect/ abuse	13 maternity facilities	Kenya	Multi-component multi-system multi-site change programme, including policy maker engagement, training in values and attitudes transformation; quality improvement teams; caring for carers; D&A monitoring; mentorship; maternity open days; community workshops; mediation/alternative dispute resolution; Counselling community members who had experienced D&A	**Physical and verbal abuse** **Violations of confidentiality** and privacy **Detainment** Abandonment	*Explicit*: gender theory/discrimination, structural disrespect *Implicit*: social engagement, hierarchy, systems theory; socio-ecological theory; behaviour change theories
Asefa 2020 [14, 15]	Before/ after	Reduction in mistreatment	3 maternity facilities	Ethiopia	3 day interactive training workshop including presentations, role play, videos and hospital visits, using a manual based on human rights, the law, ethics and continuous quality improvement. Labour ward wall posters with WRA universal rights, and with WHO positive childbirth experience infographics. Post training quality improvement facility visits including checklist based appraisal based on observations, documents and interviews. Gap analysis resulting in escalation of solutions to hospital administrators	Verbal abuse **Physical abuse** **Non-consented care** Lack of information, privacy, dignity Neglect, discrimination **Refusal of preference**	*Explicit*: none *Implicit*: normalisation theory (of verbal abuse); systems theory; socio-ecological theory; behaviour change theories
Brown 2007 [266]	RCT	Increase in labour companions (as a means to respectful care)	10 maternity facilities	South Africa	Access to the WHO Reproductive Health Library and linked training, plus: an educational intervention to promote childbirth companions, introduction of WHO RHL facilities, including an interactive workbook and workshop; posters and banners encouraging women to bring in a companion; illustrated pamphlets for staff and pregnant women to show how companionship could be promoted locally; a magazine style video on birth companionship including interviews with recent South African mothers and with staff. Encouragement by the research team for senior staff to attend the workshop. Visits by research team every two weeks to discuss progress, and how to overcome obstacles.	birth companionship **mobility (favoured control group)** routine **episiotomy**, enema verbal abuse physical abuse abandonment	*Explicit*: none *Implicit*: social support theory
Kujawski 2017 [267]	Before/ after	Reduction in disrespect/abuse	2 maternity facilities	Tanzania	1) Participatory process with multiple community, policy and facility stakeholders, designed to create a Client Service Charter built on consensus on norms to foster mutual respect and respectful care. The Charter was then widely disseminated in communities and local health facilities (6 months). 2) Quality Improvement process in one local facility to address D&A as a system-level issue, using plan-do-act type cycles with local staff, resulting in a number of changes at ward and facility level, including provision of curtains to ensure privacy, transparency about stock-outs, running continuous customer satisfaction exit surveys, providing tea for on-shift staff, best-practice sharing with other wards and the regional hospital, counselling staff who showed D&A behaviours, and mutual staff encouragement to exhibit respectful care	Non confidential care Non dignified care (incl verbal abuse) **Neglect** Non-consented care **Physical abuse** Inappropriate demands for payment	*Explicit*: normalisation theory *Implicit*: systems theory; socio-ecological theory; behaviour change theories
Mihret 2020 [265]	Before/ after	Reduction in disrespect/ abuse	1 maternity facility	Ethiopia	Multi-component package across the organisation based on prior qualitative work with a senior multi-disciplinary team	**Overall disrespect and abuse**: subscales: (**physical abuse, non-consented care, non-confidential care, non-dignified care**, **discrimination and neglected care**)	None
Ratcliffe 2016 [268, 269] *[148]*	Before/ after	Reduction in disrespect/ abuse	1 maternity facility	Tanzania	A three-part step-wise dissemination and participatory process with local stakeholders from the facility, district community, and national representatives, and a multi-stakeholder working group. Two components were developed. The first (May-Oct 2014) was a series of Open Birth Days (antenatal education, communication, and information sessions for women re birth and what would happen to them in hospital, their rights, what they should bring in, open discussions between attendees and staff to build trust, tours of the hospital, including the complaints department; accompanied by posters of the ‘universal rights of childbearing women’, translated into Kiswahili and hung on all the wards, notebook copies sent to all staff, and postcard copies given to all women attending the sessions). The second was a Respectful Care Workshop, held over 6 sessions over 2 days, ending with an agreed action plan agreed by each participating group, based on the WHO Health Workers for Change curriculum.	Measures mostly of **changes in knowledge among service users and attitudes providers about D&A, and provider communication and job satisfaction**. Service user **satisfaction with services** and perceptions of **quality of care** and of **respect shown by providers**	*Explicit*: none *Implicit*: logic model (called ‘theory of change’ in the text) developed based on knowledge, communication and attitudes; systems theory; socio-ecological theory
Umbeli 2011 [271]	Before/ after	Improving communication during labour	1 maternity facility	Sudan	Training of registrars, house officers, midwives and data collectors on communication skills, support during childbirth, providing information, and empathy.	information to women: **onset of labour, investigations** **vaginal examination, antibiotics**, adverse effect of drugs and procedures informed consent views of staff as supportive, friendly and respectful	*Explicit*: communication theory I*mplicit*: none

In general, maternity studies in this area have included multi-component interventions, at various levels of the maternity care system, based on prior development work with key stakeholders. All the included studies report improvements in at least some of their outcomes. The one study that was largely focused on educational interventions alone reduced rates of episiotomy, but did not have a significant effect on overall verbal or physical abuse [266]

To assess the extent to which the theories, models or approaches referenced might fit with the socio-ecological-behavioural approach proposed in our synthesis, we broadly aligned the intervention components described in the studies to both the socio-ecological and the behavioural elements (Table 7).

**Table 7:** Mapping published studies of respectful care interventions in maternity services to the proposed socio-ecological-behavioural approach.

*Socio-ecological components*	*Behavioural beliefs*	*Normative beliefs*	*Control beliefs*
Culture (macro level)	Kujawski [267] Abuya [270]	Kujawski [267] Abuya [270]	? Kujawski [267]^?^ Abuya [270]
Policy (government level)	? Kujawski [267]? Ratcliffe [268] Abuya [270]	? Kujawski [267] Ratcliffe [268] Abuya [270]	? Kujawski [267]^?^ Ratcliffe [268] Abuya [270]
Community/organisations	Abuya [270] Asefa [14, 15] Brown [266] Kujawski [267] Mihret [265] Ratcliffe [268]	Abuya [270] Asefa [14, 15] Brown [266] Kujawski [267] Mihret [265] Ratcliffe [268]	Abuya [270]? Asefa [14, 15] Brown [266]? Kujawski [267] Mihret [265] Ratcliffe [268]
Interpersonal	Abuya [270] Asefa [14, 15] Brown [266] Kujawski [267] Mihret [265] Ratcliffe [268] Umbali [271]	Abuya [270] Asefa [14, 15] Brown [266] Kujawski [267] Mihret [265] Ratcliffe [268] Umbali [271]	? Abuya [270] Asefa [14, 15] Brown [266] Kujawski [267] Mihret [265] Ratcliffe [268] Umbali [271]
Individual	Abuya [270] Asefa [14, 15] Brown [266] Kujawski [267] Mihret [265] Ratcliffe [268] Umbali [271]	Abuya [270] Asefa [14, 15] Brown [266] Kujawski [267] Mihret [265] Ratcliffe [268] Umbali [271]	? Abuya [270] Asefa Asefa [14, 15] Brown [266] Kujawski [267] Mihret [265] Ratcliffe [268] Umbali [271]

This exercise demonstrates that the components of the proposed socio-ecological-behavioural approach can account for interventions that have been tested, with some success, in the maternity setting in low-income countries. It also demonstrates, however, that most interventions in the maternity field are focused on the personal, interpersonal and community/organisation level of the socio-ecological model, and on the normative and behavioural benefit components of behavioural change. At least in the published accounts we located, there appears to be less evidence of interventions targeted at the policy and cultural system level, though community and staff engagement was apparent in some of the included studies. Including these aspects more systematically in future interventions may impact on longer term sustainability, and the potential for a successful roll-out.

Unlike the non-maternity studies, some of the controlled maternity studies do address verbal abuse. However, even where this is done, there does not seem to be strong evidence of effectiveness in reducing such abuse in most cases (see Table 6).

### Observations on critical contexts and mechanisms of effect

**Fig 11:**
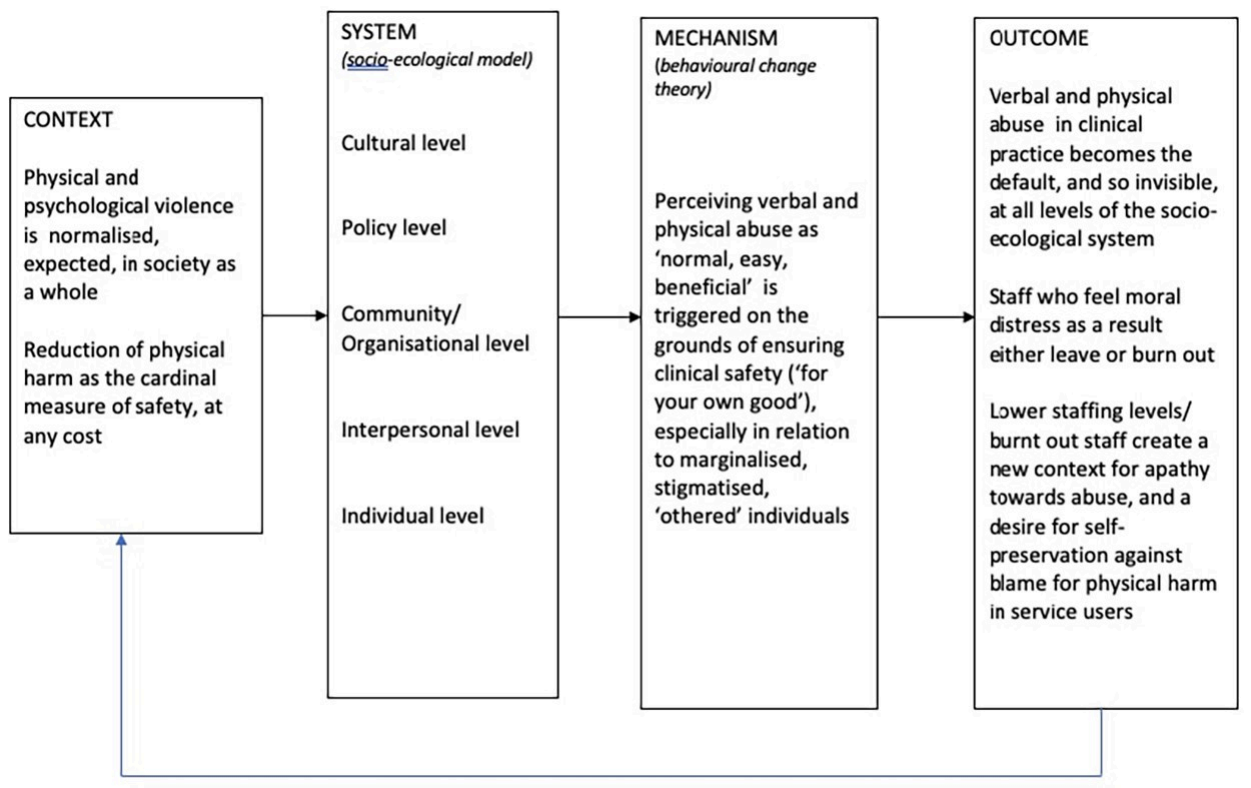
Logic model for contexts and mechanisms integrated with the socio-ecological-behavioural approach.

This review describes the large spectrum of theories and models that have been used to explain the occurrence of verbal and physical abuse from health and social care staff towards those to whom they are providing care. Beyond these disparate theories, however, there appear to be two distinct contextual features across the range of included papers that appear to be associated with verbal and physical abuse, and that, we hypothesise, could map across the proposed socio-ecological-behavioural model. These are described in the logic model in Fig 11.

In critical realist theory, mechanisms have been conceptualised as phenomena which, when triggered by specific contexts, produce specific outcomes through the resources and reasoning they generate [272]. They can catalyse ‘demi-regularities’ or similar outcomes, when similar contexts operate in different settings. We hypothesise that the two contextual features that emerged from a number of the studies we included may trigger ‘mechanisms of effect’ for verbal and physical abuse. These mechanisms could be discrimination, intersectional victimisation, stereotyping, bias, burnout, moral distress, or any of the other theoretical explanations described in the included studies.

The first context is captured in the ‘normalisation’ theories logged against a number of studies in the review. The hypothesis is that behavioural intentions, attitudes, and actions (mechanisms) of discrimination, ‘othering’, staff burnout and etc; are triggered in contexts where ***violence is normalised across all levels of the socio-ecological system***. The relevant contexts are those where violence is an everyday, and even trivial, ‘fact of life’, to the extent that it becomes expected, invisible, and, therefore, structurally embedded. Service users expect it, and report that they ‘deserve’ it, or that it is necessary for them to be abused, to ensure they are ‘safe’ (in psychiatric care) or that their baby is born ‘safely’. Professionals expect to carry it out, and their organisations do not see such violence as requiring disciplinary action–indeed, if there is an adverse event and physical or verbal pressure has not been applied, organisations may hold the practitioner to account. Local communities anticipate that physical and verbal violence will be required when they attend health care settings, not least because such behaviour is normalised in general social interactions, especially in terms of violence towards women and people in other minoritized and stigmatised groups. This can be particularly evident for those experiencing intersectional marginalisation.

The second context intersects with the first. This is where there is a ***belief that physical or verbal mistreatment is necessary to minimise clinical harm***. These are contexts where physical and verbal mistreatment are justified as being in the interests of the service user (or, in maternity care, the mother and/or baby) because they are believed to reduce particular physical harms, even when there is no evidence of such benefits, and even if the mistreatment might result in emotional and/or psychological harm. Selective use of this approach can be based on the belief that people with certain characteristics are more at risk of physical harm or less able or willing to take care of themselves, or (in the case of maternity care, or restraint use in ITU/emergency departments) the belief that routine use of restrictive interventions is necessary to ensure ‘safety’ as measured by specific clinical outcomes.

This second context is paradoxical, since in most settings (including critical care, psychiatric nursing and maternity care) there is no evidence that verbal or physical violence or restraint improves clinical outcomes, and their use can be associated with iatrogenic harm. Indeed, in papers included in this review, some staff experience moral distress as a consequence of the dissonance between peer, organisational and societal imperatives to act with violence or physical restraint to reduce physical risk, and their professional identity as someone who has a compassionate duty to prevent emotional and psychological harm to their patients. As is evident in several of the included studies, one consequent outcome is that some staff will leave, creating increased staffing pressures for those who stay. Others stay in practice, but burn out, becoming increasingly indifferent to the use of physical and verbal abuse, and thus reinforcing the behavioural and contextual norms for such abuse, at all levels of the socio-ecological model.

### Potential of the behavioural change wheel for design of future intervention studies

**Fig 12:**
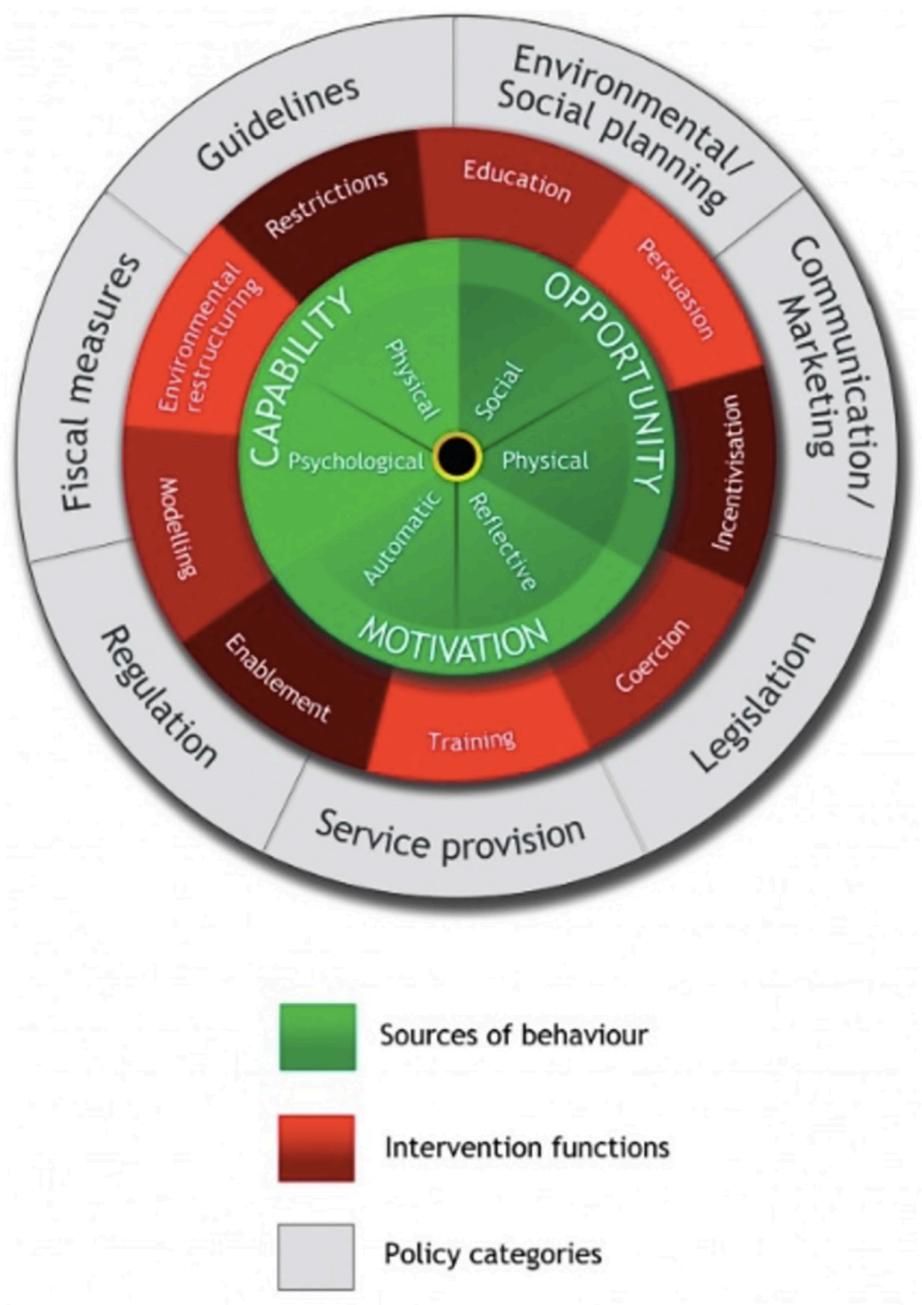
Behavioural change wheel.

While the integrated socio-ecological behavioural approach proposed in this paper explains many of the findings in the review, it does not provide an analytic tool for intervention design. In 2011, Michie and colleagues published a paper introducing the Behavioural Change Wheel (BCW) [273, 274]. The widely used COM-B behavioural change model, is at the heart of the BCW. The core drivers of behaviour in the COM-B model (Capability, Opportunity, and Motivation) have some synergy with the constructs of control beliefs, normative beliefs, and behavioural beliefs in the Theory of Planned Behaviour. However, the full BCW, as illustrated in Fig 12, also includes proposed drivers and techniques for addressing each of these behavioural drivers.

The BCW does not explicitly address all levels of the system, and it is relatively silent on the potential reciprocity between and within change at different system levels. Along with other behavioural change and implementation models, it has tended to be used to address well-defined practical changes that are not based on deep-seated values and norms (sitting behaviour, diet, handwashing, conversations about physical activity, for instance). The long-term sustainability of behavioural change following the use of such tools has not yet been widely assessed. This is important for the current review since the ‘othering’ that was identified in this study is a complex and deeply entrenched, often cross-generational social and behavioural norm, that requires a complete shift in individual, institutional, social and community beliefs and attitudes to ensure sustainable change. It remains to be seen if the new theoretical and practical behavioural tools can get beyond performative change in specific behaviours while the individuals concerned are under scrutiny, and ensure sustainable change in attitudes, beliefs and behaviours into the longer term. Despite these potential limitations, the BCW could provide a framework for developing a logic model for a multi-component intervention across the socio-ecological system to drive behavioural change in relation to normative violence against ‘othered’ groups in maternity care, and to remove the social acceptability of verbal and physical abuse as a means of optimising clinical maternal and neonatal outcomes.

## Discussion

Our search for studies that have used a theoretical lens in the area of verbal and physical abuse found a large body of knowledge across multiple health settings, using a range of theories that could be captured by both the socio-ecological model, and the theory of planned behaviour. Most of the maternity care papers that incorporated some kind of implicit or explicit theory were undertaken in low and middle-income countries. This was also true for the maternity intervention studies assessed at the synthesis stage of the review. In contrast, in the other areas of health care represented, the vast majority were from high income settings, and notably from North America. This reflects the global emphasis on safe and respectful motherhood, particularly in low and middle- income countries, in contrast to specific concerns about physical restraint in a range of health and social care settings in high income countries, particularly in elderly and psychiatric settings.

Strikingly, we found very few non-maternity studies that addressed verbal abuse, and, even in the maternity studies, where this was more likely to be an outcome measure, there was little evidence of effect. The rise of on-line and remote health and social care delivery suggests that the risk of such mistreatment is likely to rise over the coming years. This is an important gap in the literature, both in general health and social care, and in maternity services.

Meta-analysing the included intervention studies to assess which approaches and theory-based interventions might be most likely to be effective was problematic, since the only measure that was common in the eligible intervention studies was physical restraint, and only 14 provided sufficient data for statistical analysis. Even though most of the individual studies claimed benefits for their interventions, the meta-analysis indicated there was overall little or marginal benefit for interventions used to date to reduce physical restraint. Only one study in the meta-analysis included either a theoretical approach, or an intervention focused on both staff and service users. Though these did appear to be more effective than other studies in the meta-analysis, this can only be the basis for hypothesis generation for future studies, given the paucity of evidence.

The theories and operational factors discussed in the included papers in both phases of the review covered all levels of the socio-ecological model, and are similar to those identified in other papers in the Series of which this review is a part [4, 5]. The theories identified operated at the individual level (e.g. patient characteristics, staff stress/workload) the facility level (eg staff turnover, staffing levels, institutional culture), the level of policy (risk averse policies, resources and funding); and societal levels (gender inequality, marginalisation, stigma, and etc). Female gender inequality, and stereotyping, stigma and prejudice (involving immigrants, ethnic minorities, those of lower economic status, for instance) were noted as being influential and intersectional in many of the included studies/reviews.

Determining how to tackle complex multi-system problems (so called ‘wicked’ problems [275]) can seem to be an overwhelming task. Trying to resolve such complexity can lead to a sense of helplessness, and a retreat from the attempt, especially when many have tried before without success. It is for this reason that we are proposing an integration of the theory of planned behaviour with socio-ecological theory as one route to reducing verbal and physical abuse.

We are not proposing to add to the myriad of models and approaches for implementation of change that already exist. Some of these models and approaches are targeted on the index individuals in whom behavioural change is sought [264]. However, many do not include individuals at management, policy, funder and government level who have the potential power to change aspects of the socio-ecological system that could impact on behavioural change in the index individuals. Existing models could, though, be applied to such individuals in theory, if they were integrated with the socio-ecological model. The Behavioural Change Wheel [273], of which COM-B is a component, could be a useful starting point for the design of an intervention along these lines.

Whatever model, theory or approach is used, it is important to note that complex systems are not complicated. They have simple rules [276]. Complicated theoretical models are therefore unlikely to be effective. We hypothesise that the two simple rules that underpin our proposed model are that *to be effective and sustained*, *change must be fractal*, *across the whole of the relevant socio-ecological system*: and *that such sustained change depends on synergy between the beliefs*, *attitudes and norms of human actors at each level of the relevant system*. Applying a context/mechanism/outcome lens to the data suggest that two contextual factors could be critical in sustaining the mechanisms that underpin the outcome of verbal and physical abuse in health and social care from both the socio-ecological and the behavioural perspective: namely, *social normalisation of violence*, *particularly for ‘othered’ groups*, and the *belief that physical or verbal mistreatment is necessary to minimise clinical harm*.

We suggest that one way of understanding and then addressing the issue of verbal and physical mistreatment in specific maternity care systems in future is to work with local stakeholders (staff, managers, community, service users, policy makers) to establish the extent to which the ‘simple rules’, contextual drivers and theories of discrimination and stereotyping revealed in this paper, and in the other papers in the Series [4, 5] explain verbal and physical abuse locally. This mapping exercise should be done at **every level** of the local socio-ecological system, using the proposed socio-ecological-behavioural approach, alongside an existing compatible model, such as the Behavioural Change Wheel [274]. Only once this mapping, consultation or co-production, and analysis is done should the intervention be designed and then tested. This should involve stakeholders in considering how the identified drivers could be mapped to processes designed to be effective in changing the contexts that trigger harmful attitudes, norms and beliefs of the key actors at each level of that particular social system, including senior management, the local community, and political stakeholders. Consequent reductions in the harms caused by verbal and physical abuse could go beyond particular maternity care institutions, potentially improving the wellbeing of communities and staff, as well as service users.

In addition to improving the design and delivery of programmes, there is an urgent need to make sure that studies that do take place in future in this area capture outcomes consistently, both to allow for inter-study comparisons, and to optimise rapid learning and implementation of what works.

### Limitations

We could not be sure that we located all the studies that could be relevant to the question as the topic area was broad. We did not include all the physical restraint studies we originally identified, due to the very large numbers of such studies, but we did include all the intervention studies designed to reduce unnecessary physical restraint, apart from any we could have missed due to translation issues. Our search and selection criteria were rigorous, and we do not believe that any studies that we missed would change our conclusions significantly.

We did not locate any controlled studies designed to address verbal abuse as a primary outcome. This an area that is in urgent need of addressing, especially as social media and remote provision of care provides a new space in which verbal abuse could be a primary focus for distress for pregnant and postnatal women and birthing people.

We were only able to meta-analyse studies that reported on physical restraint, as this was the only outcome that was both common to a large percentage of the study data set, and that was reported with data that could be meta-analysed. This resulted in an analysis that was heavily skewed towards high income countries, as physical restraint in elderly and mental health facilities is a topic of interest in these settings. It may also be seen to be somewhat removed from the kinds of maternity care mistreatment and abuse that are often reported. However, one maternity intervention paper we located did include measures of restraint and gagging in the Ethiopian context [15] and found a base rate of about 10% in each case, with a non-significant reduction to about 5%. This suggests that physical restraint is an important part of mistreatment in some maternity contexts, and that learning from studies that have tried to address it is important, even if they are undertaken in other health and social care areas, and in high income contexts.

## Conclusions

Despite the interest in mistreatment in maternity care specifically and in health and social care more generally, there are still few intervention studies that are robust enough to be meta-analysed, and with outcomes that are consistent enough to be compared. Most non-maternity controlled studies are focused on physical restraint in elderly care and in psychiatric settings, suggesting that other areas of physical abuse, and the adverse effects of verbal abuse, have not yet been widely recognised. There is little evidence that controlled studies have been based on interventions and outcomes that take account of theoretical analysis of causation, despite the very wide and rich range of theories that have been suggested to have utility in this field. Intervention studies in maternity care seem to be more likely than those in other settings to take account of both systems level and behavioural factors in their intervention design, though with less emphasis on wider cultural and policy levels of the socio-ecological model than on those of the individual or the organisation.

Our findings suggest that the ethos and therefore the expression of D&A at each level of the system is moderated by the individuals who enact the system, and by what they feel they can control, what is socially normal, and what ultimately benefits them in that particular social context. In maternity care, systematic and routine disrespect and abuse of some women, carried out, condoned, or ignored by people operating in social groups at all system levels indicates that ‘othering’ of those with specific characteristics (depending on the particular social context) is (made) easy, normal and beneficial for individuals. In line with both the socio-ecological model, and the Theory of Planned Behaviour, our findings suggest that the solution is not to focus only on staff failure through systems level interventions (for example, through guidelines or training or audit, set up by the institution and targeted at particular behaviours of front line professionals). This may be part of the solution, but strategies based narrowly on such interventions risk being short-term, and only effective for the particular behaviours being targeted. The ultimate aim is to encourage sustainable changes in attitudes and beliefs that then permanently change a range of behaviours in individuals at all levels of the socio-ecological system, including senior staff and middle managers who set the organizational tone, politicians, organizational funders and auditors, leaders of local communities, front-line staff, and other key stakeholders. The intention is that resulting individual, group, institutional, and community norms change profoundly and sustainably to resist ‘othering’ at a fundamental level, and into the longer term, after the formal intervention programme is complete.

Using the complexity theory concept of ‘simple rules’ to address change in complex systems implies that approaches used in this field do not need to be complicated, as long as they address the simple rules of addressing socio-ecological system factors at all levels; and of influencing the attitudes and beliefs of the individuals who create these system factors, at all levels. This work needs to focus of societal normalisation of violence, particularly for ‘othered’ groups, and the belief that physical or verbal mistreatment is necessary to minimise clinical harm no matter what the cost for emotional and psychological wellbeing, It is important to combine an analysis of these factors in intervention design with local stakeholders, ideally in co-production models. Theoretically informed tools that integrate practical solutions, such as the Behavioural Change Wheel, might be helpful in designing such tailored interventions. Framing interventions that are locally determined, but which can ‘read across’ between studies, based on simple rules and realist demi-regularities is likely to maximise rapid learning between studies.

## Supporting information

S1 Table Medline (Ovid) search conducted 29th September 2020. (DOCX)

S2 Table Index search, 29^th^ Sept 2020; characteristics of included papers with theory. (PDF)

S3 Table Updated search, 22^nd^ March 2022; characteristics of included papers with theory. (XLSX)

S4 Table Index search, 29^th^ Sept 2020; characteristics of included intervention papers. (PDF)

S5 Table Updated search, 22^nd^ March 2022; characteristics of included intervention papers. (XLSX)

S6 Table Risk of bias in included intervention studies. (XLSX)

## References

[1] Bowser D, Hill K. Exploring evidence for disrespect and abuse in facility-based childbirth report of a landscape analysis. 2010 https://cdn2.sph.harvard.edu/wp-content/uploads/sites/32/2014/05/Exploring-Evidence-RMC_Bowser_rep_2010.pdf Feb 3rd 2023

[2] Bohren MA, Vogel JP, Hunter EC, Lutsiv O, Makh SK, Souza JP, et al. The mistreatment of women during childbirth in health facilities globally: a mixed-methods systematic review. PLoS Med. 2015;12(6). doi: 10.1371/journal.pmed.1001847 26126110 PMC4488322

[3] Sacks E. Defining disrespect and abuse of newborns: a review of the evidence and an expanded typology of respectful maternity care. Reprod Health 14, 66 (2017). doi: 10.1186/s12978-017-0326-1 28545473 PMC5445465

[4] Bohren MA, Vazquez Corona M, Odiase OJ, Wilson AN, Sudhinaraset M, Diamond-Smith N, et al. (2022) Strategies to reduce stigma and discrimination in sexual and reproductive healthcare settings: A mixed-methods systematic review. PLOS Glob Public Health 2(6): e0000582. doi: 10.1371/journal.pgph.0000582 36962453 PMC10021469

[5] Schaaf M, Jaffe M, Tunçalp Ö, Freedman L (2023) A critical interpretive synthesis of power and mistreatment of women in maternity care. PLOS Glob Public Health 3(1): e0000616. doi: 10.1371/journal.pgph.0000616 36962936 PMC10021192

[6] WHO Antenatal guidelines for a positive pregnancy experience 2016 https://www.who.int/publications/i/item/9789241549912. Accessed on Feb 3rd 2023

[7] WHO Intrapartum guidelines for a positive childbirth experience 2018 https://apps.who.int/iris/bitstream/handle/10665/260178/9789241550215-eng.pdf Accessed on Feb 3^rd^ 2023

[8] WHO recommendations on maternal and newborn care for a positive postnatal experience.2022 https://www.who.int/publications/i/item/9789240045989. Accessed on Feb 3^rd^ 2023 35467813

[9] Downe S, Lawrie TA, Finlayson K, Oladapo OT. Effectiveness of respectful care policies for women using routine intrapartum services: a systematic review. Reprod Health. 2018;15(1):23. doi: 10.1186/s12978-018-0466-y 29409519 PMC5801845

[10] WHO Prevention and elimination of disrespect and abuse during childbirth. 2014 https://apps.who.int/iris/bitstream/handle/10665/134588/WHO_RHR_14.23_eng.pdf Accessed on Feb 3rd 2023

[11] Bohren MA, Mehrtash H, Fawole B, Maung TM, Balde MD, Maya E et al. How women are treated during facility-based childbirth in four countries: a cross-sectional study with labour observations and community-based surveys. Lancet. 2019 Nov 9;394(10210):1750–1763. doi: 10.1016/S0140-6736(19)31992-0 31604660 PMC6853169

[12] Botngård A, Eide AH, Mosqueda L, Blekken L, Malmedal W. Factors associated with staff-to-resident abuse in Norwegian nursing homes: a cross-sectional exploratory study. BMC Health Serv Res. 2021 Mar 19;21(1):244. doi: 10.1186/s12913-021-06227-4 33740965 PMC7977325

[13] Rietbergen T, Spoon D, Brunsveld-Reinders AH, Schoones JW, Huis A, Heinen M et al. Effects of de-implementation strategies aimed at reducing low-value nursing procedures: a systematic review and meta-analysis. Implement Sci. 2020 May 25;15(1):38. doi: 10.1186/s13012-020-00995-z 32450898 PMC7249362

[14] Asefa A, Morgan A, Gebremedhin S, Tekle E, Abebe S, Magge H et al Mitigating the mistreatment of childbearing women: evaluation of respectful maternity care intervention in Ethiopian hospitals. BMJ Open. 2020a Sep 3;10(9). doi: 10.1136/bmjopen-2020-038871 32883738 PMC7473661

[15] Asefa A, Morgan A, Bohren MA, Kermode M. Lessons learned through respectful maternity care training and its implementation in Ethiopia: an interventional mixed methods study. Reprod Health. 2020b Jul 2;17(1):103. doi: 10.1186/s12978-020-00953-4 32615999 PMC7331171

[16] Higgins JPT, Thomas J, Chandler J, Cumpston M, Li T, Page MJ et al (editors) Cochrane Handbook for Systematic Reviews of Interventions version 6.0 (updated August 2022). www.training.cochrane.org/handbook. Accessed: Feb 3rd 2023

[17] Greenhalgh T, Robert G, Macfarlane F, Bate P, Kyriakidou O, Peacock R. 2005 Storylines of research in diffusion of innovation: a meta-narrative approach to systematic review. Soc Sci Med. 2005;61(2):417–430. doi: 10.1016/j.socscimed.2004.12.001 15893056

[18] Chaimani A, Caldwell DM, Li T, Higgins JPT, Carernti G 2021 Network meta-analyses. In: Cochrane Handbook for systematic reviews of Interventions, Chapter 11, https://training.cochrane.org/handbook/current/chapter-11 Accessed Feb 3rd 2023

[19] Hong QN, Pluye P, Bujold M, Wassef M. 2017 Convergent and sequential synthesis designs: implications for conducting and reporting systematic reviews of qualitative and quantitative evidence. Syst Rev.6(1):61 doi: 10.1186/s13643-017-0454-2 28335799 PMC5364694

[20] Abuya T, Sripad P, Ritter J, Ndwiga C, Warren CE. Measuring mistreatment of women throughout the birthing process: implications for quality of care assessments. Reprod Health Matters. 2018;26(53):48–61. doi: 10.1080/09688080.2018.1502018 30212308

[21] Acevedo-Nuevo M, Gonzalez-Gil MT, Solis-Munoz M, Arias-Rivera S, Torano-Olivera MJ, Carrasco et al. Physical restraint in critical care units from the experience of doctors and nursing assistants: In search of an interdisciplinary interpretation. Enferm Intensiva. 2020;31(1):19–34. 10.1016/j.enfi.2019.01.004 31253585

[22] Afulani PA, Kelly AM, Buback L, Asunka J, Kirumbi L, Lyndon A. Providers’ perceptions of disrespect and abuse during childbirth: a mixed-methods study in Kenya. Health Policy Plan. 2020;35(5):577–86. doi: 10.1093/heapol/czaa009 32154878 PMC7225569

[23] Al-Maraira OA, Hayajneh FA. Use of Restraint and Seclusion in Psychiatric Settings: A Literature Review. J Psychosoc Nurs Ment Health Serv. 2019;57(4):32–9. doi: 10.3928/02793695-20181022-01 30376587

[24] Alghamdi RS, Stockdale J, Boyle B, Perra O. Mistreatment of pregnant women at health facilities in Arab countries: a qualitative systematic review. British Journal of Midwifery. 2019;27(8):514–24.

[25] Allen JJ. Seclusion and restraint of children: a literature review. J Child Adolesc Psychiatr Nurs. 2000;13(4):159–67. doi: 10.1111/j.1744-6171.2000.tb00096.x 11883404

[26] Amroussia N, Hernandez A, Vives-Cases C, Goicolea I. "Is the doctor God to punish me?!" An intersectional examination of disrespectful and abusive care during childbirth against single mothers in Tunisia. Reprod Health. 2017;14(1):32. doi: 10.1186/s12978-017-0290-9 28259180 PMC5336668

[27] Anderson K, Bird M, MacPherson S, Blair A. How do staff influence the quality of long-term dementia care and the lives of residents? A systematic review of the evidence. Int Psychogeriatr. 2016;28(8):1263–81. doi: 10.1017/S1041610216000570 27082717

[28] Anderson RA, Issel LM, McDaniel RR Jr. Nursing homes as complex adaptive systems: relationship between management practice and resident outcomes. Nurs Res. 2003;52(1):12–21. doi: 10.1097/00006199-200301000-00003 12552171 PMC1993902

[29] Andrews GJ, Peter E. Moral geographies of restraint in nursing homes. Worldviews Evid Based Nurs. 2006;3(1):2–7. doi: 10.1111/j.1741-6787.2006.00044.x 17040517

[30] Arnold R, van Teijlingen E, Ryan K, Holloway I. Villains or victims? An ethnography of Afghan maternity staff and the challenge of high quality respectful care. BMC Pregnancy Childbirth. 2019;19(1):307. doi: 10.1186/s12884-019-2420-6 31443691 PMC6708168

[31] Asefa A, Bekele D, Morgan A, Kermode M. Service providers’ experiences of disrespectful and abusive behavior towards women during facility based childbirth in Addis Ababa, Ethiopia. Reprod Health. 2018;15(1):4. doi: 10.1186/s12978-017-0449-4 29304814 PMC5756390

[32] Ashcraft L, Anthony W. Eliminating seclusion and restraint in recovery-oriented crisis services. Psychiatr Serv. 2008;59(10):1198–202. doi: 10.1176/ps.2008.59.10.1198 18832507

[33] Asmaningrum N, Kurniawati D, Tsai YF. Threats to patient dignity in clinical care settings: A qualitative comparison of Indonesian nurses and patients. J Clin Nurs. 2020;29(5–6):899–908. doi: 10.1111/jocn.15144 31855306

[34] Attar-Schwartz S. Maltreatment by Staff in Residential Care Facilities: The Adolescents’ Perspectives. Social Service Review. 2011;85(4):635–64.

[35] Ayhan CHB, Bilgin H, Uluman OT, Sukut O, Yilmaz S, Buzlu S. A Systematic Review of the Discrimination Against Sexual and Gender Minority in Health Care Settings. International Journal of Health Services. 2020;50(1):44–61. doi: 10.1177/0020731419885093 31684808

[36] Backhaus R, Verbeek H, van Rossum E, Capezuti E, Hamers JP. Nurse staffing impact on quality of care in nursing homes: a systematic review of longitudinal studies. J Am Med Dir Assoc. 2014;15(6):383–93. doi: 10.1016/j.jamda.2013.12.080 24529872

[37] Baker PR, Francis DP, Hairi NN, Othman S, Choo WY. Interventions for preventing abuse in the elderly. Cochrane Database Syst Rev. 2016(8):CD010321. doi: 10.1002/14651858.CD010321.pub2 27528431 PMC7169376

[38] Bakker R, Sheferaw ED, Stekelenburg J, Yigzaw T, de Kroon MLA. Development and use of a scale to assess gender differences in appraisal of mistreatment during childbirth among Ethiopian midwifery students. PLoS ONE. 2020;15(1):e0227958. doi: 10.1371/journal.pone.0227958 31945110 PMC6964878

[39] Balde MD, Bangoura A, Diallo BA, Sall O, Balde H, Niakate AS, et al. A qualitative study of women’s and health providers’ attitudes and acceptability of mistreatment during childbirth in health facilities in Guinea. Reprod Health. 2017;14(1):4. doi: 10.1186/s12978-016-0262-5 28086975 PMC5237275

[40] Barbui C, Purgato M, Abdulmalik J, Caldas-de-Almeida JM, Eaton J, Gureje O, et al. Efficacy of interventions to reduce coercive treatment in mental health services: umbrella review of randomised evidence. Br J Psychiatry. 2020:1–11. 10.1192/bjp.2020.144 32847633

[41] Barr L, Wynaden D, Heslop K. Promoting positive and safe care in forensic mental health inpatient settings: Evaluating critical factors that assist nurses to reduce the use of restrictive practices. Int J Ment Health Nurs. 2019;28(4):888–98. doi: 10.1111/inm.12588 30916443

[42] Ben Natan M, Akrish O, Zaltkina B, Noy RH. Physically restraining elder residents of long-term care facilities from a nurses’ perspective. Int J Nurs Pract. 2010;16(5):499–507. doi: 10.1111/j.1440-172X.2010.01875.x 20854348

[43] Betron ML, McClair TL, Currie S, Banerjee J. Expanding the agenda for addressing mistreatment in maternity care: a mapping review and gender analysis. Reprod Health. 2018;15(1):143. doi: 10.1186/s12978-018-0584-6 30153848 PMC6114528

[44] Bhattacharya S, Sundari Ravindran TK. Silent voices: institutional disrespect and abuse during delivery among women of Varanasi district, northern India. BMC Pregnancy Childbirth. 2018;18(1):338. doi: 10.1186/s12884-018-1970-3 30126357 PMC6102865

[45] Blakeslee JA, Goldman BD, Papougenis D, Torell CA. Making the transition to restraint-free care. J Gerontol Nurs. 1991;17(2):4–8. doi: 10.3928/0098-9134-19910201-04 1902243

[46] Bohren MA, Vogel JP, Tuncalp O, Fawole B, Titiloye MA, Olutayo AO, et al. Mistreatment of women during childbirth in Abuja, Nigeria: a qualitative study on perceptions and experiences of women and healthcare providers. Reprod Health. 2017;14(1):9. doi: 10.1186/s12978-016-0265-2 28095911 PMC5240205

[47] Bourbonniere M, Strumpf NE, Evans LK, Maislin G. Organizational characteristics and restraint use for hospitalized nursing home residents. J Am Geriatr Soc. 2003;51(8):1079–84. doi: 10.1046/j.1532-5415.2003.51355.x 12890069

[48] Bower FL, McCullough CS, Timmons ME. A synthesis of what we know about the use of physical restraints and seclusion with patients in psychiatric and acute care settings: 2003 update. Online J Knowl Synth Nurs. 2003;10:1. doi: 10.1111/j.1524-475x.2003.00001.x 12800050

[49] Bowers L. Safewards: a new model of conflict and containment on psychiatric wards. J Psychiatr Ment Health Nurs. 2014;21(6):499–508. doi: 10.1111/jpm.12129 24548312 PMC4237187

[50] Bowers L, Alexander J, Bilgin H, Botha M, Dack C, James K, et al. Safewards: the empirical basis of the model and a critical appraisal. J Psychiatr Ment Health Nurs. 2014;21(4):354–64. doi: 10.1111/jpm.12085 24460906 PMC4237197

[51] Bowers L, Van Der Merwe M, Paterson B, Stewart D. Manual restraint and shows of force: the City-128 study. Int J Ment Health Nurs. 2012;21(1):30–40. doi: 10.1111/j.1447-0349.2011.00756.x 21733054

[52] Braaten KL, Malmedal W. Preventing physical abuse of nursing home residents- as seen from the nursing staff’s perspective. Nurs. 2017;4(4):274–81. doi: 10.1002/nop2.98 29085653 PMC5653394

[53] Bradley S, McCourt C, Rayment J, Parmar D. Disrespectful intrapartum care during facility-based delivery in sub-Saharan Africa: A qualitative systematic review and thematic synthesis of women’s perceptions and experiences. Soc Sci Med. 2016;169:157–70. doi: 10.1016/j.socscimed.2016.09.039 27723514

[54] Bradley S, McCourt C, Rayment J, Parmar D. Midwives’ perspectives on (dis)respectful intrapartum care during facility-based delivery in sub-Saharan Africa: a qualitative systematic review and meta-synthesis. Reprod Health. 2019;16(1):116. doi: 10.1186/s12978-019-0773-y 31345239 PMC6659209

[55] Braun KL, Suzuki KM. Developing and testing training materials on elder abuse and neglect for nurse aides. Journal of Elder Abuse & Neglect. 1997;9(1):1.

[56] Brophy LM, Roper CE, Hamilton BE, Tellez JJ, McSherry BM. Consumers and Carer perspectives on poor practice and the use of seclusion and restraint in mental health settings: results from Australian focus groups. International Journal of Mental Health Systems. 2016;10:6. doi: 10.1186/s13033-016-0038-x 26855669 PMC4744440

[57] Brower HT. The alternatives to restraints. J Gerontol Nurs. 1991;17(2):18–22. doi: 10.3928/0098-9134-19910201-07 1902242

[58] Brugnolli A, Canzan F, Mortari L, Saiani L, Ambrosi E, Debiasi M. The Effectiveness of Educational Training or Multicomponent Programs to Prevent the Use of Physical Restraints in Nursing Home Settings: A Systematic Review and Meta-Analysis of Experimental Studies. Int J Environ Res Public Health. 2020;17(18):16. doi: 10.3390/ijerph17186738 32947851 PMC7558973

[59] Bulto GA, Demissie DB, Tulu AS. Respectful maternity care during labor and childbirth and associated factors among women who gave birth at health institutions in the West Shewa zone, Oromia region, Central Ethiopia. BMC Pregnancy Childbirth. 2020;20(1):443. doi: 10.1186/s12884-020-03135-z 32746788 PMC7398399

[60] Burrowes S, Holcombe SJ, Jara D, Carter D, Smith K. Midwives’ and patients’ perspectives on disrespect and abuse during labor and delivery care in Ethiopia: a qualitative study. BMC Pregnancy Childbirth. 2017;17(1):263. doi: 10.1186/s12884-017-1442-1 28830383 PMC5567643

[61] Buzgová R, Ivanová K. Elder abuse and mistreatment in residential settings. Nursing Ethics. 2009;16(1):110–26. doi: 10.1177/0969733008097996 19103695

[62] Buzgova R, Ivanova K. Violation of ethical principles in institutional care for older people. Nursing Ethics. 2011;18(1):64–78. doi: 10.1177/0969733010385529 21285198

[63] Calvo Aguilar O, Torres Falcon M, Valdez Santiago R. Obstetric violence criminalised in Mexico: a comparative analysis of hospital complaints filed with the Medical Arbitration Commission. BMJ sex. 2019;05:05. doi: 10.1136/bmjsrh-2018-200224 31690580

[64] Cambridge P. The First Hit: a case study of the physical abuse of people with learning disabilities and challenging behaviours in a residential service. Disability & Society. 1999;14(3):285–308.

[65] Caprio TV, Katz PR, Karuza J. Commentary: The physician’s role in nursing home quality of care: focus on restraints. J Aging Soc Policy. 2008;20(3):295–304. doi: 10.1080/08959420802050868 19024029

[66] Cassie KM, Cassie W. Racial disparities in the use of physical restraints in U.s. nursing homes. Health Soc Work. 2013;38(4):207–13. doi: 10.1093/hsw/hlt020 24432487

[67] Castle NG. Differences in nursing homes with increasing and decreasing use of physical restraints. Med Care. 2000;38(12):1154–63. doi: 10.1097/00005650-200012000-00002 11186294

[68] Castle NG, Banaszak-Holl J. The Effect of Administrative Resources on Care in Nursing Homes. Journal of Applied Gerontology. 2003;22(3):405–24.

[69] Castle NG, Fogel B. Characteristics of nursing homes that are restraint free. Gerontologist. 1998;38(2):181–8. doi: 10.1093/geront/38.2.181 9573662

[70] Castle NG, Fogel B, Mor V. Risk factors for physical restraint use in nursing homes: pre- and post-implementation of the Nursing Home Reform Act. Gerontologist. 1997;37(6):737–47. doi: 10.1093/geront/37.6.737 9432990

[71] Castro A, Savage V, Kaufman H. Assessing equitable care for Indigenous and Afrodescendant women in Latin America. Rev Panam Salud Publica. 2015;38(2):96–109. 26581050

[72] Ceron A, Ruano AL, Sanchez S, Chew AS, Diaz D, Hernandez A, et al. Abuse and discrimination towards indigenous people in public health care facilities: experiences from rural Guatemala. Intern. 2016;15:77. doi: 10.1186/s12939-016-0367-z 27177690 PMC4866428

[73] Chandler GE. Reducing use of restraints and seclusion to create a culture of safety. J Psychosoc Nurs Ment Health Serv. 2012;50(10):29–36. doi: 10.3928/02793695-20120906-97 22998537

[74] Chapman R, Ogle KR, Martin C, Rahman A, McKenna B, Barnfield J. Australian nurses’ perceptions of the use of manual restraint in the Emergency Department: a qualitative perspective. J Clin Nurs. 2016;25(9–10):1273–81. doi: 10.1111/jocn.13159 26992047

[75] Chattopadhyay S, Mishra A, Jacob S. ’Safe’, yet violent? Women’s experiences with obstetric violence during hospital births in rural Northeast India. Cult Health Sex. 2018;20(7):815–29. doi: 10.1080/13691058.2017.1384572 29096592

[76] Conner T, Prokhorov A, Page C, Fang Y, Xiao Y, Post LA. Impairment and abuse of elderly by staff in long-term care in Michigan: evidence from structural equation modeling. J Interpers Violence. 2011;26(1):21–33. doi: 10.1177/0886260510362880 20448233

[77] Cooper C, Dow B, Hay S, Livingston D, Livingston G. Care workers’ abusive behavior to residents in care homes: a qualitative study of types of abuse, barriers, and facilitators to good care and development of an instrument for reporting of abuse anonymously. Int Psychogeriatr. 2013;25(5):733–41. doi: 10.1017/S104161021200227X 23290126

[78] Corbi G, Grattagliano I, Ivshina E, Ferrara N, Solimeno Cipriano A, Campobasso CP. Elderly abuse: risk factors and nursing role. Intern. 2015;10(3):297–303. doi: 10.1007/s11739-014-1126-z 25190624

[79] Cui N, Long M, Zhou S, Zhang T, He C, Gan X. Knowledge, Attitudes, and Practices of Chinese Critical Care Nurses Regarding Physical Restraint. J Contin Educ Nurs. 2019;50(3):121–6. doi: 10.3928/00220124-20190218-07 30835322

[80] Curran SS. Staff resistance to restraint reduction: identifying & overcoming barriers. J Psychosoc Nurs Ment Health Serv. 2007;45(5):45–50. 17526330 10.3928/02793695-20070501-09

[81] Darcy L. Reducing and/or minimising physical restraint in a high care, rural aged care facility. Int. 2007;5(4):458–67. 10.1111/j.1479-6988.2007.00083.x 21631806

[82] De Benedictis L, Dumais A, Sieu N, Mailhot MP, Letourneau G, Tran MA, et al. Staff perceptions and organizational factors as predictors of seclusion and restraint on psychiatric wards. Psychiatr Serv. 2011;62(5):484–91. doi: 10.1176/ps.62.5.pss6205_0484 21532073

[83] Delaney KR, Fogg L. Patient characteristics and setting variables related to use of restraint on four inpatient psychiatric units for youths. Psychiatr Serv. 2005;56(2):186–92. doi: 10.1176/appi.ps.56.2.186 15703346

[84] Dolan J, Dolan Looby SE. Determinants of Nurses’ Use of Physical Restraints in Surgical Intensive Care Unit Patients. Am J Crit Care. 2017;26(5):373–9. doi: 10.4037/ajcc2017244 28864433

[85] Dwekat IMM, Tengku Ismail TA, Ibrahim MI, Ghrayeb F. Exploring factors contributing to mistreatment of women during childbirth in West Bank, Palestine. Women Birth. 2020;16:16. doi: 10.1016/j.wombi.2020.07.004 32684342

[86] Eren N. Nurses’ attitudes toward ethical issues in psychiatric inpatient settings. Nursing Ethics. 2014;21(3):359–73. doi: 10.1177/0969733013500161 24091350

[87] Eskandari F, Abdullah KL, Zainal NZ, Wong LP. The effect of educational intervention on nurses’ knowledge, attitude, intention, practice and incidence rate of physical restraint use. Nurse Educ Pract. 2018;32:52–7. doi: 10.1016/j.nepr.2018.07.007 30029085

[88] Forssen AS. Lifelong significance of disempowering experiences in prenatal and maternity care: interviews with elderly Swedish women. Qual Health Res. 2012;22(11):1535–46. doi: 10.1177/1049732312449212 22745366

[89] Frazao SL, Correia AM, Norton P, Magalhaes T. Physical abuse against elderly persons in institutional settings. J Forensic Leg Med. 2015;36:54–60. doi: 10.1016/j.jflm.2015.09.002 26397767

[90] Fuzy E, Clow SE, Fouché N. ’Please treat me like a person’—respectful care during adolescent childbirth. British Journal of Midwifery. 2020;28(6):360–9.

[91] Gagnon MP, Desmartis M, Dipankui MT, Gagnon J, St-Pierre M. Alternatives to seclusion and restraint in psychiatry and in long-term care facilities for the elderly: perspectives of service users and family members. Patient. 2013;6(4):269–80. doi: 10.1007/s40271-013-0023-2 23949927

[92] Gallinagh R, Nevin R, Mc Ilroy D, Mitchell F, Campbell L, Ludwick R, et al. The use of physical restraints as a safety measure in the care of older people in four rehabilitation wards: findings from an exploratory study. International Journal of Nursing Studies. 2002;39(2):147–56. doi: 10.1016/s0020-7489(01)00020-7 11755445

[93] Gerace A, Muir-Cochrane E. Perceptions of nurses working with psychiatric consumers regarding the elimination of seclusion and restraint in psychiatric inpatient settings and emergency departments: An Australian survey. Int J Ment Health Nurs. 2019;28(1):209–25. doi: 10.1111/inm.12522 30019798 PMC7818138

[94] Gil AP. Quality procedures and complaints: nursing homes in Portugal. Journal of Adult Protection. 2019;21(2):126–43.

[95] Goethals S, Dierckx de Casterle B, Gastmans C. Nurses’ ethical reasoning in cases of physical restraint in acute elderly care: a qualitative study. Med Health Care Philos. 2013;16(4):983–91. doi: 10.1007/s11019-012-9455-z 23192571

[96] Gordon SE, Dufour AB, Monti SM, Mattison ML, Catic AG, Thomas CP, et al. Impact of a Videoconference Educational Intervention on Physical Restraint and Antipsychotic Use in Nursing Homes: Results From the ECHO-AGE Pilot Study. J Am Med Dir Assoc. 2016;17(6):553–6. doi: 10.1016/j.jamda.2016.03.002 27161317 PMC5331906

[97] Grilo Diniz CS, Rattner D, Lucas d’Oliveira AFP, de Aguiar JM, Niy DY. Disrespect and abuse in childbirth in Brazil: social activism, public policies and providers’ training. Reprod Health Matters. 2018;26(53):19–35. doi: 10.1080/09688080.2018.1502019 30106349

[98] Gunawardena R, Smithard DG. The Attitudes Towards the Use of Restraint and Restrictive Intervention Amongst Healthcare Staff on Acute Medical and Frailty Wards-A Brief Literature Review. Geriatr. 2019;4(3):04. doi: 10.3390/geriatrics4030050 31487923 PMC6787583

[99] Hadi F, Khosravi T, Shariat SV, Jalali Nadoushan AH. Predictors of physical restraint in a psychiatric emergency setting. Med J Islam Repub Iran. 2015;29:296. 26913259 PMC4764265

[100] Hajizadeh K, Vaezi M, Meedya S, Mohammad Alizadeh Charandabi S, Mirghafourvand M. Prevalence and predictors of perceived disrespectful maternity care in postpartum Iranian women: a cross-sectional study. BMC Pregnancy Childbirth. 2020;20(1):463. doi: 10.1186/s12884-020-03124-2 32795326 PMC7427776

[101] Hall KS, Manu A, Morhe E, Dalton VK, Challa S, Loll D, et al. Bad girl and unmet family planning need among Sub-Saharan African adolescents: the role of sexual and reproductive health stigma. Qual Res Med Healthc. 2018;2(1):55–64. doi: 10.4081/qrmh.2018.7062 30556052 PMC6292434

[102] Hameed W, Avan BI. Women’s experiences of mistreatment during childbirth: A comparative view of home- and facility-based births in Pakistan. PLoS ONE. 2018;13(3):e0194601. doi: 10.1371/journal.pone.0194601 29547632 PMC5856402

[103] Hamers JP, Bleijlevens MH, Gulpers MJ, Verbeek H. Behind Closed Doors: Involuntary Treatment in Care of Persons with Cognitive Impairment at Home in the Netherlands. J Am Geriatr Soc. 2016;64(2):354–8. doi: 10.1111/jgs.13946 26805454

[104] Hamers JP, Meyer G, Kopke S, Lindenmann R, Groven R, Huizing AR. Attitudes of Dutch, German and Swiss nursing staff towards physical restraint use in nursing home residents, a cross-sectional study. International Journal of Nursing Studies. 2009;46(2):248–55. doi: 10.1016/j.ijnurstu.2008.06.007 18656876

[105] Hamilton D, Griesdale D, Mion LC. The prevalence and incidence of restraint use in a Canadian adult intensive care unit: A prospective cohort study. Canadian Journal of Critical Care Nursing. 2017;28(3):25–33.

[106] Hammervold UE, Norvoll R, Aas RW, Sagvaag H. Post-incident review after restraint in mental health care -a potential for knowledge development, recovery promotion and restraint prevention. A scoping review. BMC Health Serv Res. 2019;19(1):235. doi: 10.1186/s12913-019-4060-y 31014331 PMC6480590

[107] Hantikainen V. Physical restraint: a descriptive study in Swiss nursing homes. Nursing Ethics. 1998;5(4):330–46. doi: 10.1177/096973309800500406 9782918

[108] Hantikainen V. Nursing staff perceptions of the behaviour of older nursing home residents and decision making on restraint use: a qualitative and interpretative study. J Clin Nurs. 2001;10(2):246–56. doi: 10.1046/j.1365-2702.2001.00468.x 11820346

[109] Hantikainen V, Kappeli S. Using restraint with nursing home residents: a qualitative study of nursing staff perceptions and decision-making. J Adv Nurs. 2000;32(5):1196–205. doi: 10.1046/j.1365-2648.2000.01590.x 11115005

[110] Hardin SB, Magee R, Stratmann D, Vinson MH, Owen M, Hyatt EC. Extended care and nursing home staff attitudes toward restraints. Moderately positive attitudes exist. J Gerontol Nurs. 1994;20(3):23–31. doi: 10.3928/0098-9134-19940301-06 8157876

[111] Harding G, King L. Adhering to the principles of restraint free environments in residential aged care: a literature review. Geriaction. 2005;23(2):13–21.

[112] Hasan AA, Abulattifah A. Psychiatric nurses’ knowledge, attitudes, and practice towards the use of physical restraints. Perspect Psychiatr Care. 2019;55(2):218–24. doi: 10.1111/ppc.12335 30430581

[113] Hassouneh-Phillips D, McNeff E, Powers L, Curry MA. Invalidation: a central process underlying maltreatment of women with disabilities. Women Health. 2005;41(1):33–50. doi: 10.1300/J013v41n01_03 16048867

[114] Hawsawi T, Power T, Zugai J, Jackson D. Nurses’ and consumers’ shared experiences of seclusion and restraint: A qualitative literature review. Int J Ment Health Nurs. 2020;29(5):831–45. doi: 10.1111/inm.12716 32198811

[115] Heeren P, Van de Water G, De Paepe L, Boonen S, Vleugels A, Milisen K. Staffing levels and the use of physical restraints in nursing homes: a multicenter study. J Gerontol Nurs. 2014;40(12):48–54. doi: 10.3928/00989134-20140407-03 24716645

[116] Heinze C, Dassen T, Grittner U. Use of physical restraints in nursing homes and hospitals and related factors: a cross-sectional study. J Clin Nurs. 2012;21(7–8):1033–40. doi: 10.1111/j.1365-2702.2011.03931.x 22176771

[117] Hennessy CH, McNeely EA, Whittington FJ, Strasser DC, Archea CK. Perceptions of physical restraint use and barriers to restraint reduction in a long-term care facility. J Aging Stud. 1997;11(1):49–62. doi: 10.1016/s0890-4065(97)90011-6 11774882

[118] Hopton J. Control and restraint in contemporary psychiatric nursing: some ethical considerations. J Adv Nurs. 1995;22(1):110–5. doi: 10.1046/j.1365-2648.1995.22010110.x 7560517

[119] Huang HC, Huang YT, Lin KC, Kuo YF. Risk factors associated with physical restraints in residential aged care facilities: a community-based epidemiological survey in Taiwan. J Adv Nurs. 2014;70(1):130–43. doi: 10.1111/jan.12176 23734585

[120] Hulton LA, Matthews Z, Stones RW. Applying a framework for assessing the quality of maternal health services in urban India. Soc Sci Med. 2007;64(10):2083–95. doi: 10.1016/j.socscimed.2007.01.019 17374551

[121] Hyde P, Burns D, Killett A, Kenkmann A, Poland F, Gray R. Organisational aspects of elder mistreatment in long term care. Quality in Ageing & Older Adults. 2014;15(4):197–209.

[122] Ishola F, Owolabi O, Filippi V. Disrespect and abuse of women during childbirth in Nigeria: A systematic review. PLoS ONE. 2017;12(3):e0174084. doi: 10.1371/journal.pone.0174084 28323860 PMC5360318

[123] Jewkes R, Abrahams N, Mvo Z. Why do nurses abuse patients? Reflections from South African obstetric services. Soc Sci Med. 1998;47(11):1781–95. doi: 10.1016/s0277-9536(98)00240-8 9877348

[124] Jungari S, Sharma B, Wagh D. Beyond Maternal Mortality: A Systematic Review of Evidences on Mistreatment and Disrespect During Childbirth in Health Facilities in India. Trauma Violence Abuse Rev J. 2019:1524838019881719. doi: 10.1177/1524838019881719 31630667

[125] Lach HW. Changing the Practice of Physical Restraint Use in Acute Care. J Gerontol Nurs. 2016;42(2):17–26. 26820185 10.3928/00989134-20160113-04

[126] Lai CKY, Kong SKF, Chow SKY, Lee JCS, Lok CKW. A restraint reduction program in a local old age home. Asian Journal of Nursing Studies. 2003;6(2):1–10.

[127] Lei R, Jiang X, Liu Q, He H. Nurse education to reduce physical restraints use in ICU: A scoping review. Nurs Crit Care. 2020;23:23. doi: 10.1111/nicc.12557 32969127

[128] Lim J. Factors Affecting Mistreatment of the Elderly in Long-Term Care Facilities. Healthcare (Basel). 2020;8(3):23. doi: 10.3390/healthcare8030224 32717835 PMC7551777

[129] Luiselli JK. Physical restraint of people with intellectual disability: a review of implementation reduction and elimination procedures. Journal of Applied Research in Intellectual Disabilities. 2009;22(2):126–34.

[130] Martin B, Mathisen L. Use of physical restraints in adult critical care: a bicultural study. Am J Crit Care. 2005;14(2):133–42. 15728955

[131] Mercier E, Nadeau A, Brousseau AA, Emond M, Lowthian J, Berthelot S, et al. Elder Abuse in the Out-of-Hospital and Emergency Department Settings: A Scoping Review. Ann Emerg Med. 2020;75(2):181–91. doi: 10.1016/j.annemergmed.2019.12.011 31959308

[132] Mion LC, Sandhu SK, Khan RH, Ludwick R, Claridge JA, Pile J, et al. Effect of situational and clinical variables on the likelihood of physicians ordering physical restraints. J Am Geriatr Soc. 2010;58(7):1279–88. doi: 10.1111/j.1532-5415.2010.02952.x 20579166

[133] Mitchell DA, Panchisin T, Seckel MA. Reducing Use of Restraints in Intensive Care Units: A Quality Improvement Project. Crit Care Nurse. 2018;38(4):e8–e16. doi: 10.4037/ccn2018211 30068727

[134] Mohler R, Richter T, Kopke S, Meyer G. Interventions for preventing and reducing the use of physical restraints in long-term geriatric care. Cochrane Database Syst Rev. 2011(2):CD007546. doi: 10.1002/14651858.CD007546.pub2 21328295 PMC8978305

[135] Mohler R, Richter T, Kopke S, Meyer G. Interventions for preventing and reducing the use of physical restraints in long-term geriatric care—a Cochrane review. J Clin Nurs. 2012;21(21–22):3070–81. doi: 10.1111/j.1365-2702.2012.04153.x 22978254

[136] Moore S. The relativity of theory: applying theories of social psychology to illuminate the causes of the abuse of older people in care homes. Journal of Adult Protection. 2019;21(2):89–110.

[137] B Natan M, A Lowenstein. Feature. Study of factors that affect abuse of older people in nursing homes. Nursing Management—UK. 2010;17(8):20–4. doi: 10.7748/nm2010.12.17.8.20.c8143 21229867

[138] Natan MB, Lowenstein A, Eisikovits Z. Psycho-social factors affecting elders’ maltreatment in long-term care facilities. Int Nurs Rev. 2010;57(1):113–20. doi: 10.1111/j.1466-7657.2009.00771.x 20487483

[139] Nay R, Koch S. Overcoming restraint use: examining barriers in Australian aged care facilities. J Gerontol Nurs. 2006;32(1):33–8. doi: 10.3928/0098-9134-20060101-13 16475463

[140] Oluoch-Aridi J, Smith-Oka V, Milan E, Dowd R. Exploring mistreatment of women during childbirth in a peri-urban setting in Kenya: experiences and perceptions of women and healthcare providers. Reprod Health. 2018;15(1):209. doi: 10.1186/s12978-018-0643-z 30558618 PMC6296108

[141] Paterson B. How corrupted cultures lead to abuse of restraint interventions. Learning Disability Practice. 2011;14(7):24–8.

[142] Perez D, Peters K, Wilkes L, Murphy G. Physical restraints in intensive care-An integrative review. Aust Crit Care. 2019;32(2):165–74. doi: 10.1016/j.aucc.2017.12.089 29559190

[143] Phillips LR, Guo G. Mistreatment in assisted living facilities: complaints, substantiations, and risk factors. Gerontologist. 2011;51(3):343–53. doi: 10.1093/geront/gnq122 21278080

[144] Pillemer K. Maltreatment of patients in nursing homes: overview and research agenda. J Health Soc Behav. 1988;29(3):227–38. 3071547

[145] Pillemer K, Bachman-Prehn R. Helping and hurting. Research on Aging. 1991;13(1):74.

[146] Pillemer K, Moore DW. Highlights from a study of abuse of patients in nursing homes. Journal of Elder Abuse & Neglect. 1990;2(1–2):5–29.

[147] Rainier NC. Reducing physical restraint use in alcohol withdrawal patients: a literature review. Dccn. 2014;33(4):201–6. doi: 10.1097/DCC.0000000000000059 24895949

[148] Ratcliffe HL, Sando D, Mwanyika-Sando M, Chalamilla G, Langer A, McDonald KP. Applying a participatory approach to the promotion of a culture of respect during childbirth. Reprod Health. 2016;13(1):80. doi: 10.1186/s12978-016-0186-0 27424514 PMC4948103

[149] Riahi S, Thomson G, Duxbury J. An integrative review exploring decision-making factors influencing mental health nurses in the use of restraint. J Psychiatr Ment Health Nurs. 2016;23(2):116–28. doi: 10.1111/jpm.12285 26809740

[150] Sando D, Kendall T, Lyatuu G, Ratcliffe H, McDonald K, Mwanyika-Sando M, et al. Disrespect and abuse during childbirth in Tanzania: are women living with HIV more vulnerable? J Acquir Immune Defic Syndr. 2014;67 Suppl 4:S228–34. 25436822 10.1097/QAI.0000000000000378 PMC4251905

[151] Santiago RV, Monreal LA, Rojas Carmona A, Dominguez MS. "If we’re here, it’s only because we have no money…" discrimination and violence in Mexican maternity wards. BMC Pregnancy Childbirth. 2018;18(1):244. 29914421 10.1186/s12884-018-1897-8 PMC6006746

[152] Saveman B, Astrom S, Bucht G, Norberg A. Elder abuse in residential settings in Sweden. Journal of Elder Abuse & Neglect. 1999;10(1/2):43–60.

[153] Schiamberg LB, Oehmke J, Zhang Z, Barboza GE, Griffore RJ, Von Heydrich L, et al. Physical abuse of older adults in nursing homes: a random sample survey of adults with an elderly family member in a nursing home. Journal of Elder Abuse & Neglect. 2012;24(1):65–83. doi: 10.1080/08946566.2011.608056 22206513

[154] Sharma G, Penn-Kekana L, Halder K, Filippi V. An investigation into mistreatment of women during labour and childbirth in maternity care facilities in Uttar Pradesh, India: a mixed methods study. Reprod Health. 2019;16(1):7. doi: 10.1186/s12978-019-0668-y 30674323 PMC6345007

[155] Sheferaw ED, Bazant E, Gibson H, Fenta HB, Ayalew F, Belay TB, et al. Respectful maternity care in Ethiopian public health facilities. Reprod Health. 2017;14(1):60. doi: 10.1186/s12978-017-0323-4 28511685 PMC5434569

[156] Shimoda K, Leshabari S, Horiuchi S. Self-reported disrespect and abuse by nurses and midwives during childbirth in Tanzania: a cross-sectional study. BMC Pregnancy Childbirth. 2020;20(1):N.PAG–N.PAG. doi: 10.1186/s12884-020-03256-5 33023499 PMC7542114

[157] Shrivastava S, Sivakami M. Evidence of ’obstetric violence’ in India: an integrative review. J Biosoc Sci. 2020;52(4):610–28. doi: 10.1017/S0021932019000695 31722765

[158] Silverman BC, Stern TW, Gross AF, Rosenstein DL, Stern TA. Lewd, crude, and rude behavior: the impact of manners and etiquette in the general hospital. Psychosomatics. 2012;53(1):13–20. doi: 10.1016/j.psym.2011.08.011 22221717

[159] Smith J, Banay R, Zimmerman E, Caetano V, Musheke M, Kamanga A. Barriers to provision of respectful maternity care in Zambia: results from a qualitative study through the lens of behavioral science. BMC Pregnancy Childbirth. 2020;20(1):26. doi: 10.1186/s12884-019-2579-x 31918682 PMC6953303

[160] Solnes Miltenburg A, van Pelt S, Meguid T, Sundby J. Disrespect and abuse in maternity care: individual consequences of structural violence. Reprod Health Matters. 2018;26(53):88–106. doi: 10.1080/09688080.2018.1502023 30132403

[161] Souza KJ, Rattner D, Gubert MB. Institutional violence and quality of service in obstetrics are associated with postpartum depression. Rev Saude Publica. 2017;51:69. doi: 10.1590/S1518-8787.2017051006549 28746574 PMC5510781

[162] Stevens M, Biggs S, Dixon J, Tinker A, Manthorpe J. Interactional perspectives on the mistreatment of older and vulnerable people in long-term care settings. Br J Sociol. 2013;64(2):267–86. doi: 10.1111/1468-4446.12017 23713559

[163] Stewart D, Van der Merwe M, Bowers L, Simpson A, Jones J. A review of interventions to reduce mechanical restraint and seclusion among adult psychiatric inpatients. Issues Ment Health Nurs. 2010;31(6):413–24. doi: 10.3109/01612840903484113 20450344

[164] Sullivan-Marx EM, Strumpf NE, Evans LK, Baumgarten M, Maislin G. Predictors of continued physical restraint use in nursing home residents following restraint reduction efforts. J Am Geriatr Soc. 1999;47(3):342–8. doi: 10.1111/j.1532-5415.1999.tb02999.x 10078898

[165] Taxis JC. Ethics and praxis: alternative strategies to physical restraint and seclusion in a psychiatric setting. Issues Ment Health Nurs. 2002;23(2):157–70. doi: 10.1080/016128402753542785 11901660

[166] Unoki T, Hamamoto M, Sakuramoto H, Shirasaka M, Moriyasu M, Zeng H, et al. Influence of mutual support and a culture of blame among staff in acute care units on the frequency of physical restraint use in patients undergoing mechanical ventilation. Acute med. 2020;7(1):e479. doi: 10.1002/ams2.479 31988791 PMC6971454

[167] Vacaflor CH. Obstetric violence: a new framework for identifying challenges to maternal healthcare in Argentina. Reprod Health Matters. 2016;24(47):65–73. doi: 10.1016/j.rhm.2016.05.001 27578340

[168] Varon M, Tabak N. Restraining patients as part of hospital policy. Med Law. 1996;15(3):571–87. 9009606

[169] Vedam S, Stoll K, Taiwo TK, Rubashkin N, Cheyney M, Strauss N, et al. The Giving Voice to Mothers study: inequity and mistreatment during pregnancy and childbirth in the United States. Reprod Health. 2019;16(1):77. doi: 10.1186/s12978-019-0729-2 31182118 PMC6558766

[170] Via-Clavero G, Guardia-Olmos J, Falco-Pegueroles A, Gil-Castillejos D, Lobo-Civico A, De La Cueva-Ariza L, et al. Factors influencing critical care nurses’ intentions to use physical restraints adopting the theory of planned behaviour: A cross-sectional multicentre study. Aust Crit Care. 2020;33(5):426–35. doi: 10.1016/j.aucc.2019.09.003 32331708

[171] Warren CE, Ndwiga C, Sripad P, Medich M, Njeru A, Maranga A, et al. Sowing the seeds of transformative practice to actualize women’s rights to respectful maternity care: reflections from Kenya using the consolidated framework for implementation research. BMC Womens Health. 2017;17(1):69. doi: 10.1186/s12905-017-0425-8 28854925 PMC5577789

[172] Warren CE, Njue R, Ndwiga C, Abuya T. Manifestations and drivers of mistreatment of women during childbirth in Kenya: implications for measurement and developing interventions. BMC Pregnancy Childbirth. 2017;17(1):102. doi: 10.1186/s12884-017-1288-6 28351350 PMC5371243

[173] Warren N, Beebe M, Chase RP, Doumbia S, Winch PJ. Negenegen: Sweet talk, disrespect, and abuse among rural auxiliary midwives in Mali. Midwifery. 2015;31(11):1073–80. 26299369 10.1016/j.midw.2015.07.007

[174] Abraham J, Kupfer R, Behncke A, Berger-Hoger B, Icks A, Haastert B, et al. Implementation of a multicomponent intervention to prevent physical restraints in nursing homes (IMPRINT): A pragmatic cluster randomized controlled trial. International Journal of Nursing Studies. 2019;96:27–34. doi: 10.1016/j.ijnurstu.2019.03.017 31014546

[175] Ahmadi M, Bagheri-Saweh MI, Nouri B, Mohamadamini O, Valiee S. Effect of Interventional Educational Programs on Intensive Care Nurses’ Perception, Knowledge, Attitude, and Practice About Physical Restraints: A Pre-/Postclinical Trial. Crit Care Nurs Q. 2019;42(1):106–16. doi: 10.1097/CNQ.0000000000000244 30507671

[176] Andersen C, Kolmos A, Andersen K, Sippel V, Stenager E. Applying sensory modulation to mental health inpatient care to reduce seclusion and restraint: a case control study. Nord J Psychiatry. 2017;71(7):525–8. doi: 10.1080/08039488.2017.1346142 28719249

[177] Barton SA, Johnson MR, Price LV. Achieving restraint-free on an inpatient behavioral health unit. J Psychosoc Nurs Ment Health Serv. 2009;47(1):34–40. doi: 10.3928/02793695-20090101-01 19227108

[178] Beaulieu C, Wertheimer JC, Pickett L, Spierre L, Schnorbus T, Healy W, et al. Behavior management on an acute brain injury unit: evaluating the effectiveness of an interdisciplinary training program. J Head Trauma Rehabil. 2008;23(5):304–11. doi: 10.1097/01.HTR.0000336843.60961.b7 18815507

[179] Bell A, Gallacher N. Succeeding in Sustained Reduction in the use of Restraint using the Improvement Model. BMJ qual. 2016;5(1). doi: 10.1136/bmjquality.u211050.w4430 27335641 PMC4915305

[180] Black V, Bobier C, Thomas B, Prest F, Ansley C, Loomes B, et al. Reducing seclusion and restraint in a child and adolescent inpatient area: implementation of a collaborative problem-solving approach. Australas. 2020:1039856220917081. doi: 10.1177/1039856220917081 32378414

[181] Blair EW, Woolley S, Szarek BL, Mucha TF, Dutka O, Schwartz HI, et al. Reduction of Seclusion and Restraint in an Inpatient Psychiatric Setting: A Pilot Study. Psychiatr Q. 2017;88(1):1–7. doi: 10.1007/s11126-016-9428-0 26897657

[182] Borckardt JJ, Madan A, Grubaugh AL, Danielson CK, Pelic CG, Hardesty SJ, et al. Systematic investigation of initiatives to reduce seclusion and restraint in a state psychiatric hospital. Psychiatr Serv. 2011;62(5):477–83. doi: 10.1176/ps.62.5.pss6205_0477 21532072

[183] Bowers L, Brennan G, Flood C, Lipang M, Oladapo P. Preliminary outcomes of a trial to reduce conflict and containment on acute psychiatric wards: City Nurses. Journal of Psychiatric & Mental Health Nursing (Wiley-Blackwell). 2006;13(2):165–72. doi: 10.1111/j.1365-2850.2006.00931.x 16608471

[184] Crosland KA, Cigales M, Dunlap G, Neff B, Clark HB, Giddings T, et al. Using staff training to decrease the use of restrictive procedures at two facilities for foster care children. Research on Social Work Practice. 2008;18(5):401–9.

[185] Dike CC, Lamb-Pagone J, Howe D, Beavers P, Bugella BA, Hillbrand M. Implementing a program to reduce restraint and seclusion utilization in a public-sector hospital: Clinical innovations, preliminary findings, and lessons learned. Psychol Serv. 2020;17:17. 10.1037/ser0000502 32940500

[186] Donat DC. An analysis of successful efforts to reduce the use of seclusion and restraint at a public psychiatric hospital. Psychiatr Serv. 2003;54(8):1119–23. doi: 10.1176/appi.ps.54.8.1119 12883139

[187] Duxbury J, Baker J, Downe S, Jones F, Greenwood P, Thygesen H, et al. Minimising the use of physical restraint in acute mental health services: the outcome of a restraint reduction programme (’REsTRAIN YOURSELF’). International journal of nursing studies. 2019;95:40–8. doi: 10.1016/j.ijnurstu.2019.03.016 31009823

[188] Godfrey JL, McGill AC, Jones NT, Oxley SL, Carr RM. Anatomy of a transformation: a systematic effort to reduce mechanical restraints at a state psychiatric hospital. Psychiatr Serv. 2014;65(10):1277–80. doi: 10.1176/appi.ps.201300247 25123671

[189] Goulet MH, Larue C, Lemieux AJ. A pilot study of "post-seclusion and/or restraint review" intervention with patients and staff in a mental health setting. Perspect Psychiatr Care. 2018;54(2):212–20. doi: 10.1111/ppc.12225 28635150

[190] Holstead J, Lamond D, Dalton J, Horne A, Crick R. Restraint Reduction in Children’s Residential Facilities: Implementation at Damar Services. Residential Treatment for Children & Youth. 2010;27(1):1–13.

[191] Huizing AR, Hamers JP, Gulpers MJ, Berger MP. Short-term effects of an educational intervention on physical restraint use: a cluster randomized trial. BMC geriatr. 2006;6:17. doi: 10.1186/1471-2318-6-17 17067376 PMC1635553

[192] Huizing AR, Hamers JP, Gulpers MJ, Berger MP. A cluster-randomized trial of an educational intenvention to reduce the use of physical restraints with psychogeriatric nursing home residents. J Am Geriatr Soc. 2009;57(7):1139–48. 19558484 10.1111/j.1532-5415.2009.02309.x

[193] Huizing AR, Hamers JP, Gulpers MJ, Berger MP. Preventing the use of physical restraints on residents newly admitted to psycho-geriatric nursing home wards: a cluster-randomized trial. International Journal of Nursing Studies. 2009;46(4):459–69. doi: 10.1016/j.ijnurstu.2008.03.005 18486133

[194] Johnson K, Curry V, Steubing A, Diana S, McCray A, McFarren A, et al. A non-pharmacologic approach to decrease restraint use. Intensive Crit Care Nurs. 2016;34:12–9. doi: 10.1016/j.iccn.2015.08.004 26652790

[195] Koczy P, Becker C, Rapp K, Klie T, Beische D, Buchele G, et al. Effectiveness of a multifactorial intervention to reduce physical restraints in nursing home residents. J Am Geriatr Soc. 2011;59(2):333–9. doi: 10.1111/j.1532-5415.2010.03278.x 21314651

[196] Kopke S, Muhlhauser I, Gerlach A, Haut A, Haastert B, Mohler R, et al. Effect of a guideline-based multicomponent intervention on use of physical restraints in nursing homes: a randomized controlled trial. Jama. 2012;307(20):2177–84. doi: 10.1001/jama.2012.4517 22618925

[197] Lin YL, Liao CC, Yu WP, Chu TL, Ho LH. A Multidisciplinary Program Reduces Over 24 Hours of Physical Restraint in Neurological Intensive Care Unit. J Nurs Res. 2018;26(4):288–96. doi: 10.1097/jnr.0000000000000251 29389807

[198] McCue RE, Urcuyo L, Lilu Y, Tobias T, Chambers MJ. Reducing restraint use in a public psychiatric inpatient service. J Behav Health Serv Res. 2004;31(2):217–24. doi: 10.1007/BF02287384 15255229

[199] Pellfolk TJ, Gustafson Y, Bucht G, Karlsson S. Effects of a restraint minimization program on staff knowledge, attitudes, and practice: a cluster randomized trial. J Am Geriatr Soc. 2010;58(1):62–9. doi: 10.1111/j.1532-5415.2009.02629.x 20122041

[200] Putkonen A, Kuivalainen S, Louheranta O, Repo-Tiihonen E, Ryynanen OP, Kautiainen H, et al. Cluster-randomized controlled trial of reducing seclusion and restraint in secured care of men with schizophrenia. Psychiatr Serv. 2013;64(9):850–5. doi: 10.1176/appi.ps.201200393 23771480

[201] Schreiner GM, Crafton CG, Sevin JA. Decreasing the use of mechanical restraints and locked seclusion. Adm Policy Ment Health. 2004;31(6):449–63. doi: 10.1023/b:apih.0000036413.87440.83 15478875

[202] Seckman A, Paun O, Heipp B, Van Stee M, Keels-Lowe V, Beel F, et al. Evaluation of the use of a sensory room on an adolescent inpatient unit and its impact on restraint and seclusion prevention. J Child Adolesc Psychiatr Nurs. 2017;30(2):90–7. doi: 10.1111/jcap.12174 28653508

[203] Singh NN, Lancioni GE, Karazsia BT, Chan J, Winton AS. Effectiveness of Caregiver Training in Mindfulness-Based Positive Behavior Support (MBPBS) vs. Training-as-Usual (TAU): A Randomized Controlled Trial. Front Psychol. 2016a;7:1549. doi: 10.3389/fpsyg.2016.01549 27766088 PMC5053082

[204] Singh NN, Lancioni GE, Karazsia BT, Myers RE. Caregiver Training in Mindfulness-Based Positive Behavior Supports (MBPBS): Effects on Caregivers and Adults with Intellectual and Developmental Disabilities. Front Psychol. 2016b;7:98. doi: 10.3389/fpsyg.2016.00098 26903906 PMC4746712

[205] Singh NN, Lancioni GE, Winton ASW, Singh AN, Adkins AD, Singh J. Mindful staff can reduce the use of physical restraints when providing care to individuals with intellectual disabilities. Journal of Applied Research in Intellectual Disabilities. 2009;22(2):194–202.

[206] Sivak K. Implementation of comfort rooms to reduce seclusion, restraint use, and acting-out behaviors. J Psychosoc Nurs Ment Health Serv. 2012;50(2):24–34. doi: 10.3928/02793695-20110112-01 22439145

[207] Smith NH, Timms J, Parker VG, Reimels EM, Hamlin A. The impact of education on the use of physical restraints in the acute care setting. J Contin Educ Nurs. 2003;34(1):26–33; quiz 46–7. doi: 10.3928/0022-0124-20030101-06 12546131

[208] Testad I, Aasland AM, Aarsland D. The effect of staff training on the use of restraint in dementia: a single-blind randomised controlled trial. Int J Geriatr Psychiatry. 2005;20(6):587–90. doi: 10.1002/gps.1329 15920716

[209] Testad I, Mekki TE, Forland O, Oye C, Tveit EM, Jacobsen F, et al. Modeling and evaluating evidence-based continuing education program in nursing home dementia care (MEDCED)—training of care home staff to reduce use of restraint in care home residents with dementia. A cluster randomized controlled trial. Int J Geriatr Psychiatry. 2016;31(1):24–32. doi: 10.1002/gps.4285 25845462

[210] Van Loan CL, Gage NA, Cullen JP. Reducing Use of Physical Restraint: A Pilot Study Investigating a Relationship-Based Crisis Prevention Curriculum. Residential Treatment for Children & Youth. 2015;32(2):113–33.

[211] Wisdom JP, Wenger D, Robertson D, Van Bramer J, Sederer LI. The New York State Office of Mental Health Positive Alternatives to Restraint and Seclusion (PARS) Project. Psychiatr Serv. 2015;66(8):851–6. doi: 10.1176/appi.ps.201400279 25930039

[212] Yeh SH, Hsiao CY, Ho TH, Chiang MC, Lin LW, Hsu CY, et al. The effects of continuing education in restraint reduction on novice nurses in intensive care units. J Nurs Res. doi: 10.1097/01.jnr.0000387508.44620.0e 15362016

[213] Acevedo-Nuevo M, Gonzalez-Gil MT, Martin-Arribas MC. Physical Restraint Use in Intensive Care Units: Exploring the Decision-Making Process and New Proposals. A Multimethod Study. Int J Environ Res Public Health. 2021;18(22):11. doi: 10.3390/ijerph182211826 34831583 PMC8623552

[214] Adams C Y. Maltrato en el adulto mayor institucionalizado realidad e invisibilidad. Rev Méd Clín Condes. 2012;23(1):84–90.

[215] Annborn A, Finnbogadottir HR. Obstetric violence a qualitative interview study. Midwifery. 2022;105:103212. doi: 10.1016/j.midw.2021.103212 34872035

[216] Botngard A, Eide AH, Mosqueda L, Blekken L, Malmedal W. Factors associated with staff-to-resident abuse in Norwegian nursing homes: a cross-sectional exploratory study. BMC Health Serv Res. 2021;21(1):244. doi: 10.1186/s12913-021-06227-4 33740965 PMC7977325

[217] Faheem A. The nature of obstetric violence and the organisational context of its manifestation in India: a systematic review. Sexual & Reproductive Health Matters. 2021;29(2):2004634. doi: 10.1080/26410397.2021.2004634 34872466 PMC8654405

[218] Garcia LM. A concept analysis of obstetric violence in the United States of America. Nursing Forum. 2020;55(4):654–63. doi: 10.1111/nuf.12482 33070371

[219] Hussein S, Dahlen HG, Ogunsiji O, Schmied V. From hopelessness to some hope: A qualitative interpretive research project to improve birthing experiences in Jordan. Sex Reprod Healthc. 2021;27:100580. doi: 10.1016/j.srhc.2020.100580 33279817

[220] Jaffre Y, Lange IL. Being a midwife in West Africa: Between sensory experiences, moral standards, socio-technical violence and affective constraints. Soc Sci Med. 2021;276:113842. doi: 10.1016/j.socscimed.2021.113842 33773475

[221] Mengesha MB, Desta AG, Maeruf H, Hidru HD. Disrespect and Abuse during Childbirth in Ethiopia: A Systematic Review. BioMed Research International. 2020;2020:8186070. doi: 10.1155/2020/8186070 33150181 PMC7603554

[222] Miranda FL, Velloso GS, Lima de O P, Rangel C S, de Almeida F H, Pinheiro PML, Neves Costa NV L. Violência obstétrica: percepções de enfermeiros obstétricos em uma maternidade de Minas Gerais. Hu Revista 45(4):415–20. https://periodicos.ufjf.br/index.php/hurevista/article/view/27818

[223] Mooney M, Kanyeredzi A. ’You get this conflict between you as a person and you in your role…that changes you’: A thematic analysis of how inpatient psychiatric healthcare staff in the UK experience restraint, seclusion, and other restrictive practices. International Journal of Mental Health Nursing. 2021;30(6):1703–12. 34494346 10.1111/inm.12926

[224] Okedo-Alex IN, Akamike IC, Nwafor JI, Abateneh DD, Uneke CJ. Multi-stakeholder Perspectives on the Maternal, Provider, Institutional, Community, and Policy Drivers of Disrespectful Maternity Care in South-East Nigeria. Int J Women Health. 2020;12:1145–59. doi: 10.2147/IJWH.S277827 33324116 PMC7733334

[225] Rabelo ARM, Duarte ED, França BD, Silva KL. The care by obstetric nurses: the encounter between self care bodies and other woman who is cared for. Esc Anna Nery Rev Enferm. 2020;24(1):e20190131–e.

[226] Ribeiro DdO, Gomes GC, Oliveira AMNd, Alvarez SQ, Gonçalves BG, Acosta DF. Obstetric violence in the perception of multiparous women. Rev gaúch enferm. 2020;41:e20190419–e. doi: 10.1590/1983-1447.2020.20190419 33237223

[227] Rossa-Roccor V, Schmid P, Steinert T. Victimization of People With Severe Mental Illness Outside and Within the Mental Health Care System: Results on Prevalence and Risk Factors From a Multicenter Study. Front Psychiatr. 2020;11:563860. doi: 10.3389/fpsyt.2020.563860 33033483 PMC7509533

[228] Saga S, Blekken LE, Nakrem S, Sandmoe A. Relatives’ experiences with abuse and neglect in Norwegian nursing homes. A qualitative study. BMC Health Serv Res. 2021;21(1):684. doi: 10.1186/s12913-021-06713-9 34247595 PMC8272837

[229] Sala VVV. La enfermedad normal: Aspectos históricos y políticos de la medicalización del parto. Sex, salud soc (Rio J). 2020(34):90–107.

[230] Santos Carer AM, Bezerra da Costa MS, Costa Maia Monteiro V, da Costa Belarmino A, de Oliveira Cavalcante K, Rodrigues Ferreira A Junior. Experiencias de puérperas sobre violencia obstétrica en la perspectiva fenomenológica. Rev cuba enferm. 2021;37(1):e3549–e.

[231] Shenton F, Smith R. Behaviour management or institutionalised repression? Children’s experiences of physical restraint in custody. Children & Society. 2021;35(1):159–75.

[232] Shepherd BF, Brochu PM. How do stereotypes harm older adults? A theoretical explanation for the perpetration of elder abuse and its rise. Aggression & Violent Behavior. 2021;57:N.PAG–N.PAG.

[233] Smithard D, Randhawa R. Physical Restraint in the Critical Care Unit: A Narrative Review. New Bioeth. 2022;28(1):68–82. doi: 10.1080/20502877.2021.2019979 35083967

[234] Solmi M, Granziol U, Boldrini T, Zaninotto L, Salcuni S. Stigma and attitudes towards restrictive practices in psychiatry among psychology students: a network and path analysis study in an Italian sample. J Ment Health. 2022;31(1):66–74 doi: 10.1080/09638237.2021.1875405 33502923

[235] Strong AE, White TL. Re-examining Norms of Disrespect and Abuse in the Second Stage of Labor in Tanzanian Maternity Care. Med Anthropol. 2021;40(4):307–21. doi: 10.1080/01459740.2021.1884075 33703977

[236] Tabong PT, Kyilleh JM, Amoah WW. Reasons for the utilization of the services of traditional birth attendants during childbirth: A qualitative study in Northern Ghana. Womens Health (Lond Engl). 2021;17:17455065211002483. doi: 10.1177/17455065211002483 33730960 PMC7983476

[237] Teece A, Baker J, Smith H. Understanding the decision-making of critical care nurses when restraining a patient with psychomotor agitation secondary to hyperactive delirium: A ’Think Aloud’ study. J Clin Nurs. 2022;31(1–2):121–33. doi: 10.1111/jocn.15889 34056784

[238] Thomann S, Hahn S, Bauer S, Richter D, Zwakhalen S. Variation in restraint use between hospitals: a multilevel analysis of multicentre prevalence measurements in Switzerland and Austria. BMC Health Serv Res. 2021;21(1):367. doi: 10.1186/s12913-021-06362-y 33879134 PMC8056521

[239] Tong JKC, Akpek E, Naik A, Sharma M, Boateng D, Andy A, et al. Reporting of Discrimination by Health Care Consumers Through Online Consumer Reviews. JAMA netw. 2022;5(2):e220715. doi: 10.1001/jamanetworkopen.2022.0715 35226076 PMC8886543

[240] Tsai PC, Cheng CH, Tzeng IS. A cross-sectional study examining the factors affecting nurses’ knowledge, attitude, and behavior toward physical restraint use. Perspectives in Psychiatric Care. 2021;22:22. doi: 10.1111/ppc.12951 34553392

[241] Wang J, Liu W, Zhao Q, Xiao M, Peng D. An Application of the Theory of Planned Behavior to Predict the Intention and Practice of Nursing Staff Toward Physical Restraint Use in Long-Term Care Facilities: Structural Equation Modeling. Psychol. 2021;14:275–87. doi: 10.2147/PRBM.S293759 33688280 PMC7936668

[242] Alostaz Z, Rose L, Mehta S, Johnston L, Dale C. Implementation of nonpharmacologic physical restraint minimization interventions in the adult intensive care unit: A scoping review. Intensive Crit Care Nurs. 2022;69:103153. doi: 10.1016/j.iccn.2021.103153 34920932

[243] Ayres S, Tracy MF. Recovery Model Implementation for a Medical/Geriatric Psychiatric Unit to Decrease Restraint and Seclusion Episodes: A Quality Improvement Project. J Am Psychiatr Nurses Assoc. 2021;27(5):355–60. doi: 10.1177/10783903211048449 34651520

[244] Azeem MW, Reddy B, Wudarsky M, Carabetta L, Gregory F, Sarofin M. Restraint Reduction at a Pediatric Psychiatric Hospital: A Ten-Year Journey. J Child Adolesc Psychiatr Nurs. 2015;28(4):180–4. doi: 10.1111/jcap.12127 26549698

[245] Baker J, Berzins K, Canvin K, Benson I, Kellar I, Wright J, et al. Non-pharmacological interventions to reduce restrictive practices in adult mental health inpatient settings: the COMPARE systematic mapping review NIHR Journals Library. 2021;9(5). 33651527

[246] Chen X, Zhuang Y, Lao Y, Qiao L, Chen Y, Guo F. Development and implementation of a novel decision support tool on physical restraint use in critically ill adult patients. Int J Nurs Pract. 2021:e12961. doi: 10.1111/ijn.12961 34075650

[247] Dixon M, Long EM. An Educational Intervention to Decrease the Number of Emergency Incidents of Restraint and Seclusion on a Behavioral Health Unit. J Contin Educ Nurs. 2022;53(2):70–6. doi: 10.3928/00220124-20220104-07 35103503

[248] Hall DK, Zimbro KS, Maduro RS, Petrovitch D, Ver Schneider P, Morgan M. Impact of a Restraint Management Bundle on Restraint Use in an Intensive Care Unit. Journal of nursing care quality. 2018;33(2):143–8. doi: 10.1097/NCQ.0000000000000273 28658189

[249] Hevener S, Rickabaugh B, Marsh T. Using a Decision Wheel to Reduce Use of Restraints in a Medical-Surgical Intensive Care Unit. Am J Crit Care. 2016;25(6):479–86. doi: 10.4037/ajcc2016929 27802948

[250] Kirk AP, McGlinsey A, Beckett A, Rudd P, Arbour R. Restraint Reduction, Restraint Elimination, and Best Practice: Role of the Clinical Nurse Specialist in Patient Safety. Clin Nurse Spec. 2015;29(6):321–8. doi: 10.1097/NUR.0000000000000163 26444510

[251] Mitchell DA, Panchisin T, Seckel MA. Reducing Use of Restraints in Intensive Care Units: A Quality Improvement Project. Crit Care Nurse. 2018;38(4):e8–e16. doi: 10.4037/ccn2018211 30068727

[252] Perers C, Backstrom B, Johansson BA, Rask O. Methods and Strategies for Reducing Seclusion and Restraint in Child and Adolescent Psychiatric Inpatient Care. Psychiatr Q. 2021;25:25. doi: 10.1007/s11126-021-09887-x 33629229 PMC8993718

[253] Shields LBE, Edelen A, Daniels MW, Flanders K. Decline in Physical Restraint Use Following Implementation of Institutional Guidelines. J Nurs Adm. 2021;51(6):318–23. doi: 10.1097/NNA.0000000000001020 34006803

[254] Wen X, Sun W, Wang Y, Zeng D, Shao Y, Zhou X. Application of Joanna Briggs Institute physical restraint standards to critical emergency department patients following CONSORT guidelines. Medicine (Baltimore). 2020;99(50):e23108. doi: 10.1097/MD.0000000000023108 33327232 PMC7738102

[255] Ye J, Xia Z, Wang C, Liao Y, Xu Y, Zhang Y, et al. Effectiveness of CRSCE-Based De-escalation Training on Reducing Physical Restraint in Psychiatric Hospitals: A Cluster Randomized Controlled Trial. Frontiers in Psychiatry. 2021;12. doi: 10.3389/fpsyt.2021.576662 33679467 PMC7928340

[256] Ajzen I, Fishbein M. The prediction of behavior from attitudinal and normative variables. Journal of experimental social Psychology. 1970 Oct 1;6(4):466–87.

[257] Kamavarapu YS, Ferriter M, Morton S, Völlm B. Institutional abuse—Characteristics of victims, perpetrators and organsations: A systematic review. Eur Psychiatry. 2017 Feb;40:45–54. Epub 2016 Nov 10. doi: 10.1016/j.eurpsy.2016.07.002 . 27837673

[258] Bronfenbrenner U. Toward an experimental ecology of human development. American Psychologist 1977 32(7), 513

[259] Bronfenbrenner U., & Ceci S. J. Nature-nurture reconceptualised: A bio-ecological model. Psychological Review 1994, 10(4), 568–586. 10.1037/0033-295x.101.4.568 7984707

[260] Best A, Greenhalgh T, Lewis S, Saul JE, Carroll S, Bitz J. Large-system transformation in health care: a realist review. Milbank Q. 2012 Sep;90(3):421–56. doi: 10.1111/j.1468-0009.2012.00670.x 22985277 PMC3479379

[261] Ajzen I., & Fishbein M. Understanding attitudes and predicting social behavior. Englewood Cliffs, NJ: Prentice-Hall 1980

[262] Fishbein M., & Ajzen I. Belief, attitude, intention and behavior: An introduction to theory and research. Reading, MA: Addison-Wesley 1975

[263] Fishbein M. & Ajzen I. Predicting and changing behavior: The Reasoned Action Approach. New York: Taylor & Francis 2010

[264] Nilsen P. Making sense of implementation theories, models and frameworks. Implementation Sci 2015 10, 53 doi: 10.1186/s13012-015-0242-0 25895742 PMC4406164

[265] Mihret H, Atnafu A, Gebremedhin T, Dellie E. Reducing Disrespect and Abuse of Women During Antenatal Care and Delivery Services at Injibara General Hospital, Northwest Ethiopia: A Pre-Post Interventional Study. Int J Women Health. 2020;12:835–47. 10.2147/IJWH.S273468 PMC7568622 33116933

[266] Brown H, Hofmeyr GJ, Nikodem VC, Smith H, Garner P. Promoting childbirth companions in South Africa: a randomised pilot study. BMC Med. 2007;5:7. doi: 10.1186/1741-7015-5-7 17470267 PMC1905915

[267] Kujawski SF, Freedman LP, Ramsey K, Mbaruku G, Mbuyita S, Moyo W, et al. Community and health system intervention to reduce disrespect and abuse during childbirth in Tanga region, Tanzania: a comparative before-and-after study. PLoS Med. 2017;14:7. doi: 10.1371/journal.pmed.1002341 28700587 PMC5507413

[268] Ratcliffe HL, Sando D, Mwanyika-Sando M, Chalamilla G, Langer A, McDonald KP. Applying a participatory approach to the promotion of a culture of respect during childbirth. Reprod Health. 2016;13(1):80. doi: 10.1186/s12978-016-0186-0 27424514 PMC4948103

[269] Ratcliffe HL, Sando D, Lyatuu GW, Emil F, Mwanyika-Sando M, Chalamilla G, et al. Mitigating disrespect and abuse during childbirth in Tanzania: an exploratory study of the effects of two facility-based interventions in a large public hospital. Reprod Health. 2016;13:79. doi: 10.1186/s12978-016-0187-z 27424608 PMC4948096

[270] Abuya T, Ndwiga C, Ritter J, Kanya L, Bellows B, Binkin N, et al. The effect of a multi-component intervention on disrespect and abuse during childbirth in Kenya. BMC Pregnancy Childbirth. 2015;15:224. doi: 10.1186/s12884-015-0645-6 26394616 PMC4580125

[271] Umbeli T, Murwan I, Kunna A, Ishmail S, Sulman MM, Elmahgoub A. Impact of health care Provider’s training on Patients’ communication during labor at Omdurman maternity hospital, Sudan. Sudan J Med Sciences. 2011;9(4):211–216

[272] Shaw J., Gray C.S., Baker G.R. Denis JL, Breton M, Gutberg J, et al. Mechanisms, contexts and points of contention: operationalizing realist-informed research for complex health interventions (2018). BMC Med Res Methodol 18, 178 doi: 10.1186/s12874-018-0641-4 30587138 PMC6307288

[273] Michie S., van Stralen M.M. & West R. The behaviour change wheel: A new method for characterising and designing behaviour change interventions. Implementation Sci 6, 42 (2011). doi: 10.1186/1748-5908-6-42 21513547 PMC3096582

[274] Michie S, Atkins L, West R. The Behaviour Change Wheel: A Guide to Designing Interventions. London: Silverback Publishing 2014. www.behaviourchangewheel.com Accessed Feb 3^rd^ 2023

[275] Colbert-Getz JM, Good B, Stevenson A, Moore KB, Lamb S. Systems Thinking to Solve Wicked Problems Like Mistreatment. Acad Med. 2021 Nov 1;96(11S):S180–S181. doi: 10.1097/ACM.0000000000004312 34705678

[276] Etz R, Miller WL, Stange KC. Simple Rules That Guide Generalist and Specialist Care. Fam Med. 2021 Sep;53(8):697–700. doi: 10.22454/FamMed.2021.463594 . 34587265

