# Supplementary material for: Theories for interventions to reduce physical and verbal abuse: A mixed methods review of the health and social care literature to inform future maternity care
Source: PLOS Glob Public Health. 2023 Apr 24;3(4):e0001594. doi: 10.1371/journal.pgph.0001594 (PMC10124898; doi:10.1371/journal.pgph.0001594)
Supplement: S1 Table — (DOCX) [file pgph.0001594.s001.docx]

| Searches | Query | Hits |
| --- | --- | --- |
| 1 | (Nurs* or midwi* or medic or medics or doctor* or physician* or staff or therapist* or attendant* or social worker* or social services or provider* or welfare worker*).tw. | 1197822 |
| 2 | ((Health* or care or caring or psychiatr* or medical) adj2 (professional* or practitioner* or personnel or worker* or employee* or assistant*)).tw | 205914 |
| 3 | 1 or 2 | 1324538 |
| 4 | ((harsh or judgemental or accuse or accusatory or accusing or threaten* or mock* or derogatory) adj3 (language or words or comments or remarks or speech or speaking or action or actions or communicat* or voice or statements)).tw. | 474 |
| 5 | ((harsh or rough*) adj4 (treatment* or medicat* or intervention* or care or examin* or handling)).tw. | 1586 |
| 6 | (rude or shout* or insult* or scold* or humiliat* or condescend* or intimidat* or yell or yelling or yelled or verbal abuse or Ridicule or ridiculing or condescend* or gag or gagged or gagging or tie down or restrain* or physical abuse or physically abus* or verbal violence or physical violence or maltreat* or mistreat* or dehumani* or abusive treatment or undignif* or non dignif*).tw. | 126171 |
| 7 | ((force or forceful) adj2 (using or "use" or used)).tw. | 10657 |
| 8 | "use of force".tw. | 778 |
| 9 | (physical force or held down or hold down or downward force or pinned down or forceful downward pressure).tw. | 735 |
| 10 | ((disrespect adj2 abuse) or (disrespectful adj2 abusive)).tw. | 148 |
| 11 | or/4-10 | 139373 |
| 12 | 3 and 11 | 9499 |
| 13 | Workplace Violence/ | 961 |
| 14 | (workplace violence or professional violence or workplace bully* or work related bully* or patient violence or patient initiated violence or cerebrovascular insult* or substance abuse).tw. | 26273 |
| 15 | (autobiography or bibliography or biography or clinical conference or comment or editorial or personal narrative or portrait).pt. | 1450922 |
| 16 | 13 or 14 or 15 | 1477235 |
| 17 | 12 not 16 | 8910 |
| 18 | exp animals/ not humans.sh. | 4738847 |
| 19 | 17 not 18 | 8818 |
