# Supplementary material for: Theories for interventions to reduce physical and verbal abuse: A mixed methods review of the health and social care literature to inform future maternity care
Source: PLOS Glob Public Health. 2023 Apr 24;3(4):e0001594. doi: 10.1371/journal.pgph.0001594 (PMC10124898; doi:10.1371/journal.pgph.0001594)
Supplement: S2 Table — (PDF) [file pgph.0001594.s002.pdf]

**Authors**	**Year published**	**Title**	**Aim of study**	Review (yes / no)	Type of review	Country	Study context - maternity care, elderly population	Type of Mistreatment (physical, verbal, physical restraint)	Explicit theoretical perspective (i.e. constructivist, feminist)?	Study design (grounded theory/phenomenology)	Participant characteristics - **staff/professionals** (age, profession, ethnicity, etc)	Participant characteristics - **patients/service-users** (age, ethnicity, etc)
Abuya, T. , Sripad, P. , Ritter, J. , Ndwiga, C. , Warren, C. E.	2018	Measuring mistreatment of women throughout the birthing process: implications for quality of care assessments	To examine prevelance of and factors associated with disrespect and abuse in maternity care (baseline measurement for Heshima project)	No	n/a	Kenya	maternity	physical, verbal	not identified	cohort study (before and after measures)	n/a	women giving birth in 13 facilities: 641 at baseline and 728 at endline
Afulani, P. A. , Kelly, A. M. , Buback, L. , Asunka, J. , Kirumbi, L. , Lyndon, A.	2020	Providers' perceptions of disrespect and abuse during childbirth: a mixed-methods study in	To examine providers' perspectives on the frequency and drivers of disrespect and abuse during facility-based childbirth in a	No	n/a	Kenya	maternity	physical, verbal	not identified	mixed methods	49 staff, various settings	n/a
Alghamdi RS, Stockdale J, boyle B, Perra B	2019	Mistreatment of pregnant women at health facilities in Arab countries: a qualitative systematic review	To explore the evidence regarding mistreatment during childbirth in Arab countries.	Yes	Qualitative	focused on Arab region	maternity	physical, verbal	not identified	n/a	Arab women in labour: 8 studies	n/a
Amroussia, N. , Hern , ez, A. , Vives-Cases, C. , Goicolea, I.	2017	"Is the doctor God to punish me?!" An intersectional examination of disrespectful and abusive care during childbirth against single mothers in Tunisia	To examine the self-perceptions and childbirth experiences of single mothers at the public healthcare facilities in Tunisia.	No	n/a	Tunisia	maternity	physical, verbal	not identified	qualitative	11 single mothers aged 19-43	n/a

Arnold, R. , van Teijlingen, E. , Ryan, K. , Holloway, I.	2019	Villains or victims? An ethnography of Afghan maternity staff and the challenge of high quality respectful care	To understand staff notions of care, varying levels of commitment, and the obstacles and dilemmas that affected standards	No	n/a	Afghanista n	maternity	verbal, neglect	not identified	ethnogr aphy	22 maternal healthcare providers (doctors, midwives, obstetricians, managers, etc) and 4 group discussions (n not specified)	16 women from diverse backgrounds
Asefa, A. , Bekele, D. , Morgan, A. , Kermode, M.	2018	Service providers' experiences of disrespectful and abusive behavior towards women during facility based childbirth in Addis Ababa, Ethiopia	To enhance understanding of service providers' experiences of D&A during facility based childbirth in health facilities in Addis Ababa.	No	n/a	Ethiopia	maternity	physical	not identified	Cross sectiona l	57 maternal health professionals	n/a

Bakker, R. , Sheferaw, E. D. , Stekelenburg, J. , Yigzaw, T. , de Kroon, M. L. A.	2020	Development and use of a scale to assess gender differences in appraisal of mistreatment during childbirth among Ethiopian midwifery students	To test hypothesis that male HCPs are less likely to mistreat patients during labour	No	n/a	Ethiopia	maternity care	physical, verbal	not identified	cross sectional/ correlation	390 final-year midwifery students	n/a
Balde, M. D. , Bangoura, A. , Diallo, B. A. , Sall, O. , Balde, H. , Niakate, A. S. , Vogel, J. P. , Bohren, M. A.	2017	A qualitative study of women's and health providers' attitudes and acceptability of mistreatment during childbirth in health facilities in Guinea	To better understand social norms and acceptablity of mistreatment using 4 scenarios (slapping, refusing to help, verbal abuse and forcing to give birth on the floor) from perspectives of women and service providers	No	n/a	Guinea	maternity care	physical, verbal	not identified	qualitative	13 midwives/nurses 5 doctors, 6 administrators	40 interviews and 8 focus groups with women of reproductive age

Betron, M. L. , McClair, T. L. , Currie, S. , Banerjee, J.	2018	Expanding the agenda for addressing mistreatment in maternity care: a mapping review and gender analysis	To examine whether and how gender inequalities and unequal power dynamics in the health system undermine quality of care or obstruct women's capacities to exercise their rights as both users and providers of maternity care.	yes	mapping	Ten were global; 19 reported on 11 countries in Africa, five in Asia, and three in Latin America.	maternity	verbal, physical	not identified	mapping review	Total 37 papers: participant breakdown not given	Total 37 papers: participant breakdown not given
Bhattacharya, S. , Sundari Ravindran, T. K.	2018	Silent voices: institutional disrespect and abuse during delivery among women of Varanasi district, northern India	To examine the prevalence and nature of abuse of women during delivery	No	n/a	India	Maternity care	physical, verbal, physical restraint	not identified	cross sectiona l/prevale nce	n/a	410 rural women who gave birth between June 2014 to August 2015 at any health facility of Varanasi district, northern India

Bohren, M. A. , Vogel, J. P. , Tuncalp, O. , Fawole, B. , Titiloye, M. A. , Olutayo, A. O. , Ogunlade, M. , Oyeniran, A. A. , Osunsan, O. R. , Metiboba, L. , Idris, H. A. , Alu, F. E. , Oladapo, O. T. , Gulmezoglu, A. M. , Hindin, M. J.	2017	Mistreatment of women during childbirth in Abuja, Nigeria: a qualitative study on perceptions and experiences of women and healthcare providers	To explore women and providers' experiences and perceptions of mistreatment during childbirth in two health facilities and catchment areas in Abuja, Nigeria.	No	n/a	Nigeria	maternity care	physical, verbal, physical restraint	not identified	qualitative explorat ory	17 midwives, 17 doctors and 9 facility administrators.	75 women of a reproductive age
Bradley, S. , McCourt, C. , Rayment, J. , Parmar, D.	2016	Disrespectful intrapartum care during facility-based delivery in sub-Saharan Africa: A qualitative systematic review and thematic synthesis of women's perceptions and experiences	To examine the drivers of disrespectful intrapartum care.	yes	Systematic review & meta-synthesis	Sub-Saharan Africa, including Sudan	maternity care	physical, verbal	not identified	review	n/a	Total 25 papers from 9 African countries: detailed characteristics given in the paper

Bradley, S. , McCourt, C. , Rayment, J. , Parmar, D.	2019	Midwives' perspectives on (dis)respectful intrapartum care during facility-based delivery in sub-Saharan Africa: a qualitative systematic review and meta-synthesis	to explore the broader drivers of (dis)respectful care during facility-based delivery in the sub-Saharan African context.	Yes	Systematic review & meta-synthesis	Sub-Saharan Africa	maternity care	physical, verbal	not identified	Qualitative review	Total 11 papers (10 studies) from 6 African countries: detailed characteristics given in the paper	n/a
Bulto, G. A. , Demissie, D. B. , Tulu, A. S.	2020	Respectful maternity care during labor and childbirth and associated factors among women who gave birth at health institutions in the West Shewa zone, Oromia region, Central Ethiopia	To assess RMC during Labor and Childbirth and associated factors amongwomen who gave-birth at health-institutions in the West Shewa zone, Central Ethiopia.	no	n/a	Ethiopia	maternity care	physical, verbal, physical restraint	not identified	cross sectional - prevalence survey	n/a	567 women who gave birth athealth institutions in the West Shewa zone

Burrowes, S. , Holcombe, S. J. , Jara, D. , Carter, D. , Smith, K.	2017	Midwives' and patients' perspectives on disrespect and abuse during labor and delivery care in Ethiopia: a qualitative study	To examine the experiences ofdisrespect and abuse in maternal care from the perspectives of both providers and patients.	no	n/a	Ethiopia	maternity	physical, verbal	not identified	mixed methods	4 midwives, 15 third year midwifery students	26 women who had given birth in the last year
Calvo Aguilar, O. , Torres Falcon, M. , Valdez Santiago, R.	Nov	Obstetric violence criminalised in Mexico: a comparative analysis of hospital complaints filed with the Medical Arbitration Commission	To analyse whether criminalising obstetric violence has been conducive to the recognition and observance of the reproductive rights of women, based on the records of poor health care complaints filed by women with the Medical Arbitration Commissions (CAMs by their Spanish initials) in two Mexican states.	no	n/a	Mexico	maternity	physical, verbal	not identified	claimed to be phenomenology: seems to be content analysis of complaints records	n/a	61 filed complaints from two Mexican states
Chattopadhyay, S. , Mishra, A. , Jacob, S.	2018	'Safe', yet violent? Women's experiences with obstetric violence during hospital births in rural Northeast India	To examine obstetric violence from the point of view of women	no	n/a	India	maternity	physical, verbal	Feminist	anthropology	Discussions with physicians and community health workers (Accredited Social Health Activists, ASHAs), nurses to contextualise the findings	66 women (25 with 'prolonged interactions'

Dwekat, I. M. M. , Tengku Ismail, T. A. , Ibrahim, M. I. , Ghrayeb, F.	2020	Exploring factors contributing to mistreatment of women during childbirth in West Bank, Palestine	To explore the views of Palestinian women and healthcare providers regarding factors contributing to the mistreatment of women during childbirth at childbirth facilities in the WestBank, Palestine.	no	n/a	Isarel	maternity	not specified (verbal mentioned)	not identified	Qualitative	5 health care providers (including nurse, midwife, public health officer, administrator roles)	6 postpartum women who had given birth vaginally within the past six weeks
Forssen, A. S.	2012	Lifelong significance of disempowering experiences in prenatal and maternity care: interviews with elderly Swedish women	To explore how the effects of harsh and humiliating treatment, experienced by a number of Swedish women in antenatal care and childbirth in the mid-20th Century, endured for the rest of their lives	no	n/a	Sweden	maternity	verbal, physical	Haraway's (1991) theory of situated knowledges	qualitative	n/a	20 elderly women

Fuzy, Elizabeth , Clow, Sheila Elizabeth , FouchÃ©, Nicola	2020	Please treat me like a person': respectful care during adolescent childbirth	To explore the lived childbirth experiences of mothers of middle adolescent age who were living in the Western Cape province of South Africa.	no	n/a	South Africa	maternity	verbal	not identified	phenomenological approach	n/a	13 mothers between 14–16 years of age
Grilo Diniz, C. S. , Rattner, D. , Lucas d'Oliveira, A. F. P. , de Aguiar, J. M. , Niy, D. Y.	2018	Disrespect and abuse in childbirth in Brazil: social activism, public policies and providers' training	To describe and analyse the role of social movements in promoting change in maternity care, and in provider training.	yes	mixed methods	various	maternity	physical and verbal	? Feminist	integrative	8 studies identified: two case studies and relevant government initiatives were identified	8 studies identified: two case studies and relevant government initiatives were identified
Hajizadeh, K. , Vaezi, M. , Meedya, S. , Mohammad Alizadeh Char , abi, S. , Mirghafourv , , M.	2020	Prevalence and predictors of perceived disrespectful maternity care in postpartum Iranian women: a cross-sectional study	To determine prevalence and predictors of perceived disrespectful maternity care among Iranian women.	no	n/a	Iran	Maternity	physical, verbal	not identified	quantitative	n/a	334 postpartum women, 6-18 hrs after birth
Hall, K. S. , Manu, A. , Morhe, E. , Dalton, V. K. , Challa, S. , Loll, D. , Dozier, J. L. , Zochowski, M. K. , Boakye, A. , Harris, L. H.	2018	Bad girl and unmet family planning need among Sub-Saharan African adolescents: the role of sexual and reproductive health stigma	To explore stigma surrounding adolescent sexual and reproductive health (SRH) and its impact on young Ghanaian women's family planning (FP) outcomes.	no	n/a	Ghana	Reproductive health/maternity	verbal	?constructivist	grounded theory	n/a	63 women and aged 15-34 years recruited from health facilities and schools

Hameed, W. , Avan, B. I.	2018	Women's experiences of mistreatment during childbirth: A comparative view of home- and facility-based births in Pakistan	To estimate the prevalence of mistreatment and types of mistreatment among women giving birth in facility- and home-based settings in Pakistan and the the association between demographics (sociodemographic ,reproductive history and empowerment status)and mistreatment, both in general and according to birth setting(whether home-or facility-based).	no	n/a	Pakistan	maternity	physical, verbal	not identified	quantitative	n/a	1,334 women who had given birth at home or in a healthcare facility over the past 12 months
Hulton, L. A. , Matthews, Z. , Stones, R. W.	2007	Applying a framework for assessing the quality of maternal health services in urban India	To present findings from the application of a framework for assessing the quality of care of institutionalmaternity services in an urban slum in India.	no	n/a	India	maternity	physical, verbal	not identified	Not identified	14 semi structured interviews	650 women who had recently given birth (community questionnaire; 70 case note reviews and exit interviews at discharge from the hospital

Ishola, F. , Owolabi, O. , Filippi, V.	2017	Disrespect and abuse of women during childbirth in Nigeria: A systematic review	To synthesize current evidence on disrespect and abuse of women during child birth in Nigeria in order to understand its nature and extent, contributing factors and consequences, and propose solutions.	yes	Mixed methods	Nigeria	maternity	physical, physical restraint	not identified	mixed methods	14 studies identified	14 studies identified

Jewkes, R. , Abrahams, N. , Mvo, Z.	1998	Why do nurses abuse patients? Reflections from South African obstetric services	To explore the question: why do nurses abuse patients, through presentation and discussion of findings of research on health seeking practices in one part of the SouthAfrican maternity services.	no	n/a	South Africa	maternity	physical, verbal	not identified	ethnogr aphy	midwives, enrolled nurses, family planning advisors,and general workers (13 interviews, 3 group discussions)	pregnant women (26) and 2 unbooked women after birth; postnatal women (1 group discussion)

Jungari, S. , Sharma, B. , Wagh, D.	2019	Beyond Maternal Mortality: A Systematic Review of Evidences on Mistreatment and Disrespect During Childbirth in Health Facilities in India	To examine current evidence on the nature and extent of disrespect and abuse (D&A),mistreatment and practices of respectful maternity care of women during childbirth in India.	yes	mixed methods	India	maternity	physical, verbal	not identified		11 studies identified	11 studies identified
Oluoch-Aridi, J. , Smith-Oka, V. , Milan, E. , Dowd, R.	Dec-18	Exploring mistreatment of women during childbirth in a peri-urban setting in Kenya: experiences and perceptions of women and healthcare providers	to explore the experiences and perceptions of both female patients and healthcare workers regarding mistreatment during childbirth	no	n/a	Kenya	Maternity	physical, verbal, neglect	not identified	qualitative	Interviews: 6 doctors (3 male) , 2 clinical officers, (both male) 8 nurses/midwives (2 male) and 4 hospital administrators(all female) from six different health facilities (mix of public and private)	46 interviews, 15 focus group discussions. Average age 30 years.

Ratcliffe, H. L. , S , o, D. , Mwanyika-S , o, M. , Chalamilla, G. , Langer, A. , McDonald, K. P.	2016	Applying a participatory approach to the promotion of a culture of respect during childbirth	To describe the enabling factors behind a successful multi-faceted intervention aimed at reducing mistreatment of childbearing women in a large referral hospital	no	n/a	Tanzania	Maternity	physical and verbal	not identified	Commentary on an associated intervention detailing the enabling characteristics behind it's apparent success	Wide variety of stakeholders from health ministry officials to regional health directors to facility managers to ward matrons and frontline staff	A number of women participated in the Facility 'Open Days' [no details given]
S,o, D. , Kendall, T. , Lyatuu, G. , Ratcliffe, H. , McDonald, K. , Mwanyika-S , o, M. , Emil, F. , Chalamilla, G. , Langer, A.	2014	Disrespect and abuse during childbirth in Tanzania: are women living with HIV more vulnerable?	To compare the reported and observed experiences of disrespect and abuse during labor and delivery of women living with HIV with HIV-negative women	N	n/a	Tanzania	Maternity	disrespect and abuse	not identified	Mixed methods	Structured questionnaires (n = 50) in-depth interviews (n = 18)	Interviews with postpartum women (n = 2000), direct observation during childbirth (n = 208),

Santiago, R. V. , Monreal, L. A. , Rojas Carmona, A. , Dominguez, M. S.	2018	"If we're here, it's only because we have no money..." discrimination and violence in Mexican maternity wards	To analyze the experiences of structural and gender discrimination against women during childbirth care at two public hospitals in Mexico.	N	n/a	Mexico	Maternity	physical and verbal	not identified	Mixed method s	Two focus groups with nursing staff (n=12), one with medical staff (n=9)	512 women surveyed: 20 of these were interviewed

Sharma, G. , Penn-Kekana, L. , Halder, K. , Filippi, V.	2019	An investigation into mistreatment of women during labour and childbirth in maternity care facilities in Uttar Pradesh, India: a mixed methods study	To investigate the nature and context of mistreatment during labour and childbirth at public and private sector maternity facilities in Uttar Pradesh, India.	no	n/a	India	maternity	physical, verbal, over and under treatment/lack of evidence based treatment	not identified	mixed methods	n/a	275 mothers and their newborns at 26 hospitals in three districts of Uttar Pradesh from 26 May to 8 July 2015
Sheferaw, E. D. , Bazant, E. , Gibson, H. , Fenta, H. B. , Ayalew, F. , Belay, T. B. , Worku, M. M. , Kebebu, A. E. , Woldie, S. A. , Kim, Y. M. , van den Akker, T. , Stekelenburg, J.	2017	Respectful maternity care in Ethiopian public health facilities	To describe the prevalence of respectful maternity care (RMC) and mistreatment of women in hospitals and health centers, and identify factors associated with occurrence of RMC and mistreatment of women during institutional labor andchildbirth services.	no	n/a	Ethopia	maternity	physical, verbal	not identified	mixed methods	n/a	240 women in 28 health centers
Shimoda, Kana , Leshabari, Sebalda , Horiuchi, Shigeko	2020	Self-reported disrespect and abuse by nurses and midwives during childbirth in Tanzania: a cross-sectional study	To measure the prevalence of self-reported disrespect and abuse (D&A) by healthcare providers of women during childbirth in health facilities in Tanzania, and to clarify the factors related to D&A.	no	n/a	Tanzania	maternity	physical, verbal	not identified	survey	439 nurses, nursing assistants and midwives who had ever conducted deliveries	n/a

Shrivastava, S. , Sivakami, M.	2020	Evidence of 'obstetric violence' in India: an integrative review	To collate and analyse the extant literature on‘obstetric violence’in India and analyse findings using the comprehensive typology of Bohrenet al.(2015), highlighting any findings that do not align with this typology and to develop a framework to address obstetric violence in India from a rights-based perspective within the existing structural and social determinants of health.	yes	mixed methods	India	maternity	physical, verbal	not identified	integrative mixed methods review	16 studies included	16 studies included

Smith, J. , Banay, R. , Zimmerman, E. , Caetano, V. , Musheke, M. , Kamanga, A.	2020	Barriers to provision of respectful maternity care in Zambia: results from a qualitative study through the lens of behavioral science	Focus on the behavioral drivers of disrespect and abuse in Zambia to develop solutions with health workers and women that improve the experience of care during delivery.	No	n/a	Zambia	Maternity	Verbal and physical	not identified	Qualitati ve study	27 staff members including maternal and child health and labor ward staff and volunteers	15 women, 4 birth companions

Solnes Miltenburg, A. , van Pelt, S. , Meguid, T. , Sundby, J.	2018	Disrespect and abuse in maternity care: individual consequences of structural violence	To describe how and why women's exposure to disrespectand abuse in health facilities should be seen as symptomatic of structural violence.	no	n/a	Tanzania	Maternity	Verbal and physical	not identified	Mixed methods	n/a	14 women (age 22 to 37); observation of 25 antenatal visits, 3 births, 92 interviews
Souza, K. J. , Rattner, D. , Gubert, M. B.	2017	Institutional violence and quality of service in obstetrics are associated with postpartum depression	To investigate the association between institutional violence in obstetrics and postpartum depression (PP depression) and the potential effect of race, age, and educational level in this outcome.	No	n/a	Brazil	Maternity	Verbal and physical	not identified	Cross sectional	N/A	432 women, whose children were aged up to three months - sociodemographics detailed in Table 1

Vacaflor, C. H.	2016	Obstetric violence: a new framework for identifying challenges to maternal healthcare in Argentina	To critically explore the concept of obstetric violence as a legal framework for identifying healthcare practices that constitute abuse and mistreatment of women.	No	n/a	Argentina	Maternity	Verbal and physical	Theoretical article to consider legal framework/ethical issues underpinning obstetric violence	Concept /opinion article	N/A	N/A
Vedam, S. , Stoll, K. , Taiwo, T. K. , Rubashkin, N. , Cheyney, M. , Strauss, N. , McLemore, M. , Cadena, M. , Nethery, E. , Rushton, E. , Schummers, L. , Declercq, E. , Council, G. VtM-US Steering	2019	The Giving Voice to Mothers study: inequity and mistreatment during pregnancy and childbirth in the United States	To use indicators developed by service users to describe mistreatment in childbirth in the US.	No	n/a	USA	Maternity	All	not identified	participatory research approach to survey design	n/a	Women who experienced at least one pregnancy in the United States between 2010 and 2016, including those currently pregnant. 2138/2700 completed all sections of the survey

Warren, C. E. , Ndwiga, C. , Sripad, P. , Medich, M. , Njeru, A. , Maranga, A. , Odhiambo, G. , Abuya, T.	2017	Sowing the seeds of transformative practice to actualize women's rights to respectful maternity care: reflections from Kenya using the consolidated framework for implementation research (Heshima project)	To capture and explain the complexity and interconnectedness of the elements of Heshima. Heshima was one of the first projects globally that measured the prevalence of disrespect and abuse during childbirth and designed and developed interventions based on the results from the baseline study.	No	n/a	Kenya	Maternity	All	not identified	Participatory Implementation Research in 13 facilities	A wide range of different stakeholders taking part in a range of data collection processes over the time of the study - not clear if all data collection processes were mutually exclusive, so total n not possible to report, but a minimum of 56	641 women discharged from postnatal wards at baseline compared with 728 at endline to assess the impact of the interventions. 20 interviews with community members and 51 with service users

Warren, C. E. , Njue, R. , Ndwiga, C. , Abuya, T.	2017	Manifestation and drivers of mistreatment of women in Kenya: implications for measurement and developing interventions	To describe mistreatment of women during childbirth in Kenya.	No	n/a	Kenya	Maternity	Verbal and physical	Not identified	Qualitati ve, thematic	63 local policy makers health managers and providers	19 women and men living locally: 50 women who had given birth in one of 13 included facilities:

Warren, N. , Beebe, M. , Chase, R. P. , Doumbia, S. , Winch, P. J.	2015	Negenegen: Sweet talk, disrespect, and abuse among rural auxiliary midwives in Mali	To explore disrespect and abuse toward women in labor from the perspective of auxiliary midwives.	No	n/a	Mali	Maternity	Mainly verbal/physical	not identified	cross-sectional, descriptive mixed-methods study	67 mostly rural auxiliary midwives	n/a

Data collection methods	Apriori theory used to analyse/frame the data?	Key findings/ Themes (Author text)	Explicit theories used to interpret findings (includes reference/clearly defined)	Theories/ideologies implicit by authors (but not referenced/explicity named)	Other interpretations used to explain the findings
exit interviews with mothers, observations of delivery and labour	Human rights theory	Authors argue that their findings are likely to be the result of a combination of the intervention and contextual factors (i.e. free maternity care, unchanged staffing	Not identified	social engagement, hierarchy, systems theory; socio-ecological theory; behaviour change theories	
mixed methods survey	Not identified	Drivers of disrespect and abuse included perceptions of women being difficult, stress and burnout, facility culture and lack of	Not identified	Provider/Implicit bias' (Labelling theory); social norms; cultural norms (social and organisational);/facility) gender based/horizontal violence, burnout/Trauma theory: street level	Heightened arousal due to fear of being blamed for loss of baby (particularly in low resource setting with high mortality and where high stafff turnover and burnout) with disrespect and abuse justified as
n/a	Not identified	thematic analysis of studies revealed the following themes: power and control (controlling knowledge, women's bodies and birthing	not identified	Patriarchy; 'power/powerlessness'; (sexual) shaming; general cultural norms; organisational culture (routines/beurocracy), inequality (labelling)	power and control as drivers of mistreatment, prioritsation of insitutional rules over womens rights or choice, social distances created by patriarchal attitudes, social inequality and sexual shaming
semi-structured interviews	Intersectionality	Three themes emerged during the data analysis: 1) Experiencing disrespect and abuse, 2) Perceptions ofregret and shame attributed to being a single mother, and 3) The triad of vulnerability: stigma, social challenges, andhealth system challenges.	Intersectionality; stigma	shame (being a single mother), social and cultural norms (e.g. religious beliefs), social stigma, marginalization (multiple identities), organisational norms, moral prejudices, medicalisation of childbirth, power, discrimination, patriarchy, social construction (of motherhood) low quality maternity care (poor working conditions, heavy work load, and shortage in financial	The bad image of single mothers included being morally wrong but also being incapable of making decisions and assuming responsibilities as a mother. socio-economic marginalization because of being singlemothers, but also because they had basic education and came from poor families: also impacts of institutional faliure (poor quality resources and corrupt practices)

observation, background interviews, semi-structured interviews, focus groups	poor working conditions, violence in the surrounding area, lack of status of women, health care providers ignorance and disregard of women's rights (positioning of providers as oscillating between villains or victims).	Staff blamed the workload, lack of a shift system, insufficient supplies and inadequate support from management, but closer inspection revealed a complexity of interrelated factors such as low resources made worse through theft reduced and where staff were unfairly blamed by management and where motivated staff tried hard to work well but, admitted that they lost patience and shouted at women in childbirth. There were extreme examples of both abusive and vulnerable staff.	not identified	insitutional culture/norms (not kindness and respect: peer pressure), scarity of resources, healthcare hierarchy (abuse of power), bullying, structural factors; socio-ecological; victimisation (of health care staff):	workload pressures (too many patients, inadequate staffing levels and staff exhaustion, lack of essential supplies), staff with chronic illnesses due to stresses and pressures of workload, fear of making mistakes,and being held accountable, unappreciated, under-valued and unsupported by management, a lack of teamwork and kindness (and bullying) between colleagues, staff blaming & acusations of stealing, victimisation leading to vilianous behaviour; peer pressure to conform to toxic institutional norms<br>of some staff not only for unintended mistakes but<br>also for deliberate neglect, cruelty or extortion - need for accountability
quantitative questionnaire	not identified	most service providers from these facilities had witnessed disrespectful practices during childbirth, and recognized that such practices have negative consequences for service utilization; high levels of	Not identified	organisational norms (disrespectful culture); horizontal violence; systems theory; socio-ecological theory; behaviour change theory	poor attitudes to respectful maternity practice; high workload, poor support from facility management, discomfort in the work environment; being disrespected and abused by service users and colleagues

quantitative questionnaire	Gender theory: ie, male midwives provide more respectful maternity care,which is possibly mediated by self-esteem and stress (male gender linked to greater competence and professionalism in midwifery)	No significant association between gender and mistreatment appraisal was observed and self-esteem and stress were not found to be mediators.	Gender theory	not identified	the hypothesis that male midwives were less likely to mistreat service users than female midwives, due to higher self-esteem and lower life stress, was not supported by the findings, since no differences were identified
in-depth interviews and focus groups	attitudes (behavioural theory); social norms theory	Most women were not accepting of mistreatment, unless perceived to save the life of mother or child. Women perceived a woman's disobedience and uncooperativeness contributed to the poor treatment. Women reacted to mistreatment by accepting it, refusal to	Not identified	labelling (of women as uncoperative or difficult), social norms (about violence and how women are treated), cultural norms, gender based violence, patriachy, systems theory (under-resourced healthcare systems)	acceptablity (using mistreatment (physical and shouting) on a women who is "difficult " helps the woman to focus and deliver silently or forces her to open her legs - which she may have closed for dignity) - providers did not feel that physical abuse during childbirth was acceptable, but thought that shouting was acceptable, perception that childbirth should be silent, insufficient drugs, equipment and physical infrastructure contribute

mapping search	Gender inequalities and unequal power dynamics (used a gender analysis framework for the synthesis), systems theory (under-resourced healthcare systems)	women lack information and financial assets, voice, and agency to exercise their rights. Women who defy traditional feminine stereotypes of chastity and serenity often experience mistreatment as a result. Mistreatment of women inside and outside of the health facility is normalized and accepted, including by women themselves. For health care providers, gender discrimination is manifested through	Theory of mistreatment based on gender inequalities developed using the USAID Gender Analysis Framework (based around 4 domains: access to assets, beliefs and perceptions, practices and participation, insitutions, laws and policies)	Gender inequality, gender stereotyping, patriarchy, gender discrimination, gender roles/social norms, stigma, culture norms/religious ideologies, gender-based violence, intersectionality , gender-sensitive rights-based policies	Normalization of mistreatment during childbirth, norms and stigma related to women's behavior (transgressions from gender norms/stereotypes), mistreatment and pain in childbirth punishment for dirty or sinful behaviour, Disrespect for women's abilities and limited access to sources as female health workers leading to frustration and burnout (and then mistreatment of women), Violence against women inside and outside of facilities (male to female but also female nurses deployed violence against clientsto create social distance and maintain"fantasies of iden-tity and power in their continuous struggle to asserttheir professional and middle
interview based questionnaire with open and closed responses	not identified	Associations between abuse and provider type, facility type, and presence of complications during delivery.; odds of being abused was four times higher in those women who experienced complications during delivery. prevalence of	Not identified	Systems issues (including poorly resourced and pressurised health care organisations), gender and power differences (doctors mistreating more), social acceptiblity (abuse of women acceptable if it ensures baby's safety)	Higher prevalence of mistreatment in complicated cases - most likely to be the result of doctors being involved in those cases as mistreatment was reported to be higher among doctors; but, also. a high patient load at the facilities, particularly a higher-level facility where most of the complicated cases were referred could have possibly contributed in amplifying the abusive behavior of the providers.

in-depth interviews and focus groups	not identified	Women and providers reported physical abuse including slapping, physical restraint to a delivery bed, and detainment in the hospital and verbal abuse. Women sometimes overcame tremendous barriers to reach a hospital, only to give birth on the floor, unattended by a provider. Three main factors contributing to mistreatment: poor provider attitudes, women's behavior, and	Not identified	Stigma, discrimination, system failure (poorly resourced healthcare systems, staff stress and burnout), social norms (acceptiblity of physical abuse, especially when the baby is born healthy) , sexual shaming, Insitutional norms of abuse and disrespect	Poor provider attitudes (dealing with difficult or disobedience women), women's behavior, and health systems constraints (under-staffing, over-crowding) - HCP snapping or being 'wicked' as a result of workload pressures; stigmatising of adolescents, primiparas and women of lower socioeconomic status; women who had not prepared to give birth in the facility may be more vulnerable to mistreatment because of being judged for being pregnant too young, or they are unaware of what to expect during childbirth and appear ill-prepared; lack of belief that mistreatment is happening from HCP noting lack of specifics in reporting, indicating
systematic search	Over-medicalisation of childbirth, Hierarchical and bureaucratic systems, dehumanisation of women, undersourced healthcare systems, pre-service training reinforcing class and power	Two overarching analytical themes 'Power and Control' and 'Maintaining Midwives' Status; A conceptual framework was developed to show how macro-, meso- andmicro-level drivers of disrespectful care interact. The synthesis revealed a prevailing model of maternitycare that is institution-centred, rather than woman-centred. Women's experiences illuminate midwives'efforts to maintain power and	A conceptual framework, drawing together macro-, meso- and micro-level contexts and drivers (in line with socio-ecological theory) developed from the review	Socio-ecological theory, gender/structural inequality, colonialism, intersectionality, vertical oppression; power dynamics (hierachies, rules, compliance and resistance), over-medicalisation of childbirth, social/regligious ideologies, social norms (expression of pain in labour), instititional rules (to control); (sexual) shaming	Midwives assertion of control over the birthing process, status of woman as bystander, maintance of midwives professional, technical and social status, power relationships played out in the hospital were a reflection of those in wider society, where technical skill, professional education and the ability to speak English, for example, were held in high regard, rules designed to control and avoid resitance (often implicit), wider local cultural understandings of labour pain - violation of social norms - punishment by God, birth companions not allowed and where allowed not always given the option - denying women social support can be seen as another example of midwives' efforts to retain 'Power and Control'

systematic search	Poorly resourced healthcare system, social inequalities, power dynamics, over-medicalisati on of childbirth, and the socio-ecological framework developed in Bradley at al 2016	six main themes were identified. ‘Power and control’ and ‘Maintaining midwives’ status’ reflected midwives’ focus on the micro-level interactions of the mother-midwife dyad. Meso-level drivers of disrespectful care were: the constraints of the ‘Work environment and resources’; concerns about ‘Midwives’ position in the health systems hierarchy’; and the impact of ‘Midwives’ conceptualisations of respectful maternity care’. An emerging theme outlined the ‘Impact on	Oppressed groups theory (Fiere, 1972) vs theories of professionalism	Othering, power dynamics, discrimination (racial and socioeconomic), social/institutional norms, blame culture, social inequality, poorly resourced healthcare system (deficits in the 'materiality of care'), shaming (adolescents, HIV postive), insitutional hierarchies, over-medicalisation of childbirth, bullying, social and moral superiority	maintain their professional, technical and social status of midwifes and awareness of not being respected in the healthcare system, asset midwifes control - punishment when not doing what they are told to do, women seen as not knowing what to do as justification for shouting and hitting to promote positive health outcomes, uncertainty about the skills needed to safely assist a woman and the persistence of lithotomy position, In the South African context, delivering on all fours was linked to socioeconomic and racial discrimination, widespread use of shouting or yelilng was normalised, moral judgement of younger pregnant women, midwives discriminated against certain categories of women to decide who accessed services or how
interview-based survey	Not identified	The proportion of RMC during labor and childbirth in the study area was low. Type of institution, discussionduring ANC, time of delivery, duration of stay, involvement in decision-making, the number of health workers, waiting time andconsent were	not identified	System level factors noted such as health system resourcing, and lack of supervision at night. Some service user factors also noted	There were a range of factors associated with lower levels of RMC including type of healthcare setting (better RMC at health centres than in hospitals (possibly less RMC with greater caseload), and births at night more likely to be associated lower prevelance of RMC

in-depth interviews	'Structural and individual drivers' (seems to be both socio-ecological and behavioural)	Health care providers and patients report frequent physical and verbal abuse as well as non-consented care during labor and delivery. Providers report that most abuse is unintended and results from weaknesses in the health system or from	Gender theory, stigma, organisational under-resourcing, (lack of) empowerment (women)	Health system weakness, staff burnout	
documentary analysis (complaint reports)	Theories of professional power (-over): legal power of human rights legislation.	Differences were observed regarding the contents of complaints, specifically in the categories of abuse, discrimination and neglect during childbirth. The narratives in the other complaint categories were similar between states.	not identified	sociological theory of law; empowerment to assert human rights; discrimination, gender/obstetric violence	
fieldwork (interview, converstaions, discussions, observations	Gender based violence, medicalisation, patriarchy, class discrimination.	Substantial evidence of obstetric violence reflected in iatrogenic procedures such as episiotomies, inadequate diagnosis of obstetric risks, undignified physical examinations, medical negligence, institutional unpreparedness and	Biomedical institutionalisation, discrimination (social class), gender based violence	Intersectionality, disciplining (of womens bodies), organisational normalisation (of abuse),	

interviews	not identified	Four themes were identified with regards to the women and healthcare providers' views about factors contributing to the mistreatment of women during childbirth in the WestBank, Palestine: limitation in childbirth facilities, factors within	Not identified	socio-ecological theory; poorly resourced facilities, workload pressures, lack of institutional infrastructure, stress, normalisation of misreatment (by women themselves), Instituational norms (mistreatment seen as the other way to safe lives), medicalisation of childbirth, discrimination (older women and young women, social class,	workload pressures, stress, inadequate staff to cover excessive numbers of birth (particularly on night shift), ineffective accountability mechanisms and monitoring systems, mistreatment justified to ensure coperation from women, administrative pressure, women (midwifes) having other responsiblities (social roles), primiparous women as ignorant (especially in absence of birth
interview	Foucault's (1979) theory of inscription - risk of being judged "abnormal" by others is internalized, making powerful norms work from within; Jacobson's taxonomy of dignity (Jacobson, 2007, 2009b), learned helplessnes s	Our biomedical way of understanding risks and complications during pregnancy and birthing was confronted with many participants' feelings of distress, guilt, and grief linked to their childbearing experience. This is interpreted as "violations of dignity" and as abuse. The consequences are similar to those following traumatic birth experiences described in today's literature.	Not identified	medical power, silencing (of womens knowledge and voices), social norms ('natural' birth),	

interview (with drawing activity)	Not identified	An overarching theme of preservation of personhood was identified. Three themes emerged i) unpreparedness for childbirth, ii) an unsettled state of mind during childbirth and iii)	Not identified	Victimisation - treated differently to older mothers; Othering; social norm of mistreatment	interpersonal relationships - mother and HCP, mistreatment more likely if mother alone, control given to midwifes because of the belief that this was the only way to ensure a safe birth for baby
iterative search	Social movement theory; gender perspective	To promote real change in maternity care, the progression of policies and enabling environment of laws, regulations, and broad dissemination of information, need to go hand in hand with changes in all health providers' training – including a solid base in ethics, gender and human rights.	Gender theory/intersectionality social movements theory	Social movement theory; technocratic and interventionist model of care, racial inequalities, gender violence	To promote real change...policies and enabling environment of laws, regulations, and broad dissemination of information need to go hand in had with changes in health providers training - including a solid base in ethics, gender, and human rights
cross-sectional survey	Not identified	The results showed high levels of perceived disrespectful maternity care in postpartum women. However, presence of spouses to accompany their wives in waiting rooms, the	Not identified	medicalisation of childbirth, organisational faliure (workload pressures, staff burnout)	socio-demographic factors (source of support, marital satisfaction), antenatal factors (place of prenatal care),intrapartum factors (time of birth, type of hospital, birthattendant, hospitalization duration in labour and deliveryroom, number of healthcare providers, augmentation with
interview	Stigma	Findings foused on community norms, enacted stigma, internalised stigma, non-disclosure, and stigma resilience (through social support)	Stigma, fear of contagion, gender (norms) theory	not identified	

survey	Not identified	There were no significant differences in manifestations of mistreatment between facility- and home-based childbirths. Sub-group analyses for home-based births identified the same significant associations with mistreatment,with ethnicity included. In facility-based births, there was a significant relationship between women's employment and empowerment status and mistreatment. Women with prior education on birth preparedness were less	Not identified	empowerment, ethnic discrimination	mistreatment lower in unemployed women, empowered (level of women's involvement in household decision making and prior education on birthpreparedness) women and higher in women who was assisted by traditional birth attendants rather than physicians. For home birth mistreatment varied by ethnicity
case study (multiple data collection methods)	WHO framework for QoC (experience and technical provision/organisation of care)	Evidence that quality is far from optimal in both public and private facilities. Problems identified included a lack of essential drugs, women being left unsupported,evidence of physical and verbal abuse, and births occurring in hospitals without a health	Not identified	(Lack of) equity of care quality/discrimination	

systematic search	Bowser and Hill landscape analytical framework	The type of abuse most frequently reported was non-dignified care in form of negative, poor and unfriendly provider attitude and the least frequent were physical abuse and detention in facilities. These behaviors were influenced by lowsocioeconomic	Not identified	discrimination, social sanctioning, distancing (provider > patient), organisation infrastructure, social/organisational norms (abuse as normative)	discrimination on the basis of ethnicity, low social class, low education, young age and HIV positive status, punishment for non-payment of bills/coming into hospital late, normalisation of disrespect and abuse, provider distancing as a result of training (lacking sensitivity), lack of support and supervision/accountablity, lack of training in interpersonal care

interviews and discussion groups	Not identified	Although women and staff explained nurses' treatment of them in terms of a few `rotten apples in the barrel', analysis of the data revealed a complex interplay of concerns including organisational issues, professional insecurities, perceived need to assert ``control''over the environment and sanctioning of the use of coercive and punitive measures to do so, and an underpinning ideology of patient inferiority. The findings suggest that the nurses were engaged in a continuous struggle to assert their professional and middle class identity and in the process deployed violence against patients as a means of creating social distance and maintaining fantasies of identity and	Moral evaluation of patients; theories of authoritative knowledge; (assertion of) professional power	social distancing, social class, biomedical model (patient inferiority), insitututional norms, sexual shame, workload pressures, nurse-patient relationships, insecure professional identity of midwives, vertical oppression, race theory	volence and abuse justified by staff as attempts to change practices of women that may harm their baby, ritualisation of abuse (punishment for not following rules),o rganisational issues; professional concerns, including perceptions that staff were themselves abused by patients; perceived needs to assert control over the environment and patients; social sanctioning of coercive strategies including punitive actions; and, an underpinning ideology of patient ignorance and inferiority. feelings of insecurity related to clinical roles and the difficulties of performing these without what they perceived as adequate support

systematic search	Bowser and Hill landscape analytical framework	The frequency of reported D&A was high, ranging from 10% to 77.3%. These behaviors were influenced by lack of education and empowerment of the women, their low socioeconomic status, poor training of providers and supervision, and alack of	Bowser and Hill landscape analytical framework - contributing factors were categorized into individual and community, policy, governance, providers and service delivery factors and underutilization of skilled delivery	social class, discrimination, poorly resourced medical facilities	workload pressures, poorinfrastructure, lack of training for health workers, and shortag eof trained health professionals in the government health sector
Interviews and focus groups	Framework based on Bohren's 7 different types of mistreatmen t - thematic analysis (Braun & Clarke)	6 out of 7 types of mistreatment identified:- Physical abuse, verbal abuse, stigma and discrimination, poor rapport, between healthcare workers and women, failure to meet professional standards and health systems constraints. Did NOT find Sexual Abuse. Culture of blame also identified as a contributory factor to mistreatment	Stigma, discrimination based on age, ethnicity, parity, disease status, socio-economic status ('othering'), blame culture (doctors to nurses, families to doctors and nurses, doctors and nurses to mothers). Social norms (of abuse): gender based violence; abuse as a mechanism for	Health system faliure (shortage of human and physical resources)	**Organizational culture of blame** leads to staff blaming (and abusing women) for 'having too many babies', 'being too young to be pregnant', 'not attending ANC', etc; **Social Norms** - The nurses in the study, at the end of a delivery, would often convince the patients that without the beatings their children would have been harmed and had adverse outcomes. Conversely to the concerns expressed by the women, the healthcare workers prided themselves on having been taught to be “firm” with their patients. **Structural Failings** - At the individual level

Not discussed -this is a commentary piece	Alludes to Bohren's 7 different types of mistreatment - used to inform the primary study outcomes	Main study found significant reductions in D&A outcome measures between baseline and PN following the introduction of a complex intervention incorporating 1. Open Days for expectant women to attend the facility and be shown round by staff members 2. A series of 6 interactive RMC workshops attended by key stakeholders at all levels of health system	Not identified	Abuse is linked to learned behaviour; success factors include consensus theory facilitated by a form of participatory action research; socio-ecological theory (the intervention worked at the micro, macro and meso level). Empowerment, communication, attitude, relationships	See main paper for further details
questionnaires, interviews, observation	Stigma	No differences in D&A between women living with HIV and women who were HIV-ve but relatively high levels of D&A in both groups (12.2% vs 15%). However none of the WLWH were asked for consent prior	Not identified	(Lack of) Stigma	

Cross sectional survey, interviews, focus groups	Theory of discrimination according to Krieger: structural violence	According to Krieger [3], discrimination is a socially structured and sanctioned phenomenon identifiable by the preeminence of a dominant social group. Justified by the ideology of the dominant actors, discrimination translates into individual and institutional interactions that ensure the privileges of the dominant group. Expressions denoting stigmatization and discrimination against women on the part of health-care professionals were identified in the narratives of both women and professionals with regard to the following characteristics: (a) physical appearance, (b) poverty and (c) status as women. Furthermore, the self-perception of belonging to a disadvantaged social class was identified in	Stigma, discrimination	Structural, gender, socio-economic discrimination, social normalisation of violence, exacerbated by women's passive acceptance of mistreatment and lack of understanding of their rights as well as health professionals ignorance of fundamental rights and a lack of regulation. (Intersectionality)	

Clinical observations and open-ended comments recorded by observers	Not identified	Mistreatment of women frequently occurs in both private and public sector facilities. Comments by observers provide further contextual insights into the quantitative data, and additional themes of mistreatment, such as deficiencies in infection	Not identified	Social class, discrimination, poorly resourced medical facilities, inequalities of information, wealth, and power, institutionalised bribery (informal payments)	mistreatment higher women attending district hospitals, women above 35 years, primiparous, those that were referred from another facility, women belonging to the"scheduled caste and tribe", those in the fifth (richest) wealth quintile, and amongst cases admitted during work-hours on weekday in the public sector; births conducted by unqualified personnel leading to anxiety, resource-
Observation	Not identified	Health centers demonstrated higher RMC performance than hospitals. At least one form of mistreatment of women was committed in 36% of the observations (38% in health centers and 32% in hospitals). Quality improvement using SBM-R© and having a companion during labor and delivery were associated with RMC	Not identified	Social distance, power and identity (women > women); gender theory; burnout	Having a birth companion, type of health worker (males more likely to provide RMC) - triple burdens faced by female midwives: (1) reproductive (childbearing), (2) productive (economic), and (3) community management (e.g. un-paid work in support of the community). The effect of social, economic and professional barriers resulted in moral distress and burn out, which may have led to abusive behavior: quality of care improved by companionship and implementation of SBM-R©quality improvement approach
cross-sectional survey	Working conditions predict disrespect and abuse, and D&A undermines trust between women and health care providers	D&A scores increased with an increase in 'working hours per week' and 'taking a break during evening shifts'. D&A scores decreased with an increase in the scores of the'two components of the Index of Working Satisfaction (professional status andinteraction between nurses)', and 'any type of	Demoralisation, decreased motivation, burnout	Workplace dynamics (relational), professional identity	Work load pressures and workplace satisfaction, heavy workload, poor relations with co-workers, (lack of) pride ointheir own job and lack of supervision, more breaks during night shifts linked to higher levels of D&A, higher job motivation linked to professional identity

systematic search	Gender based violence; medicalisation; gender inequality; womens rights-based perspective	'Obstetric violence' in India was found to be associated with socio-demographic factors, with women of lower social standing experiencing greater levels of mistreatment. In response to this normalized public health issue, a multi-pronged, rights-based framework is proposed that addresses the social, political and structural contexts of 'obstetric violence' in India....heavy workload, poor relation with co-workers, pride in their own job, and lack of supervision related to self-reported D&A	Cultural health capital (poor women and with lower social standing experience abuse), framework of obstetic violence suggested including enabling factors (social factors, harmful cultural practices, systemic barriers, historic normalisation) and areas for intervention	Stigma, discrimination, poorly resourced facilities (skills of staff), medical treatment norms, acceptance of abuse (norms), normalisation of gender-based violence	Low socio-economic status most likely to experience mistreatment, not having a skilled birth attendant present - medical neglience, chaotic and unsafe facilities, punishment for non-payment for services, harmful tradition practices and beliefs (i.e. fasting to dry the mothers body), delivery complications, expectation of suppression of pain (woman to be slient)

Observations, interview	Behavioural design theory	1) providers do not consider the decision to provide respectfulcare because they believe they are doing what they are expected to do, 2) providers do not consider the decision to provide respectful care explicitly since abuse and violence are normalized and therefore the default, 3) providers may decide that the costs of providing respectful care outweigh the gains, 4) providers believe they do not need to provide respectful care, and 5) providers may change their mind about the quality of care they will provide when they believe that disrespectful care will assist their clinical objectives. Context related issues associated with the barriers are supervisory systems, visual cues, social constructs, clinical processes, and other	Mental model focused on clinical functions and death avoidance - automaticity directing actions (based on cognitive availability) , cognitive scarcity	Stigma; organisation and cultural norms of violence; power and control	Lack of training/awareness; lack of guidelines; lack of consequences; low income status of patient; lack of feedback mechanisms; lack of provider-client rapport; (mis) perception that clients value it; prevention of complaints of negligence directs focus to preventing infant death (at cost to the mother); clients do not follow provider instructions due to previous negative births; they take herbs/remedies to speed up labour (which complicates the work of the provider)

Observations, interview	Gender inequalities; structural violence; burnout; health system faliures	Elements of supportive care were identified((but not extracted); Details of types of unsupportive behaviours mapped in the tables, e.g. Psychological abuse, abandonment, privacy violation, non-supportive care; qualitative analysis was themed - Normalisation of absence of care; Justification of punishment and rewards;	Authoritative knowledge	Systemic gender inequality; normalisation of abuse; organisational culture of disrespect/abuse; power; structural inequalities (i.e. hierarchical power structures for providers	health systemchallenges; poor working conditions
Questionnaire	States 'conceptual model of quality of care at childbirth used to build the indicators of violence' (based on one of the author's thesis)	institutional violence in obstetric care are positively associated with postpartum depression, Positive interactions between: violence by negligence by health care professionals and race and age; physical violence from health care professionals and age; and, verbal violence from health care professionals and race.	Social and organisational normalisation of absence of care, of violence, and of abuse, gender inequality	Organisational norms, (behavioural change theory), power inequalities (women and staff), vertical discrimination	Black, indigenous, and migrant women are the ones who suffer more discrimination in their access to health care services and who are usually the most vulnerable to this type of institutional violence.

N/A	Violence against women	Obstetric violence is a public health issue; a human rights violation; unethical gender stereotyping	Gender theory	Over medicalisation; converting natural processes of reproduction into pathological ones; gender stereotyping/masculine medical culture (treating them as vulnerable individuals, incapable of controlling their own bodies or understanding their own experiences).	
Survey	Discrimination on the grounds of ethnicity	One in six women (17.3%) reported experiencing one or more types of mistreatment such as: loss of autonomy; being shouted at, scolded,or threatened; and being ignored, refused, or receiving no response to requests for help. Context of care (e.g. mode of birth; transfer; difference of opinion) correlated with increased reports of mistreatment. Experiences of mistreatment differed significantly by place of	Discrimination (specifically on grounds of ethnicity), Intersectionality	Organisational norms; medicalisation	

Focus group discussions, in-depth interviews, and dialogues with participants and beneficiaries, observations of client-provider interactions, client exit interviews, provider interviews, facility inventories, before and after survey of prevalence data.	Yes, through use of the Consolidated Framework for Implementation Research CFIR. Participatory action research also led to tailoring of the intervention package in relation to local power dynamics and human rights issues, including discrimination and unequal power relations, between	Critical element: readiness to change. Need for flexibility and adaptability. Also important: Participatory and consultative processes; RMC workshops with attention to staff wellbeing; Community infolvement; Consensus building. Changes were affected by shiffing national maternity policies. CFIR was partially useful, but PAR was a critical element	Not identified	Systems theory. Power and resistance to power (Foucault?). The change process included attention to whole system issues of power and control (implicitly socio-ecological theory), and to creation of initial conditions for tipping points (all implicit) namely: values clarification, advocacy; community and national dialogue, new resources and curricula, mentorship, quality improvement, mediation training, conselling for providers, transparancy (open days in the facilities), monitoring of D&A, community outreach and inreach, male involvement/education. Caring for the carers was emphasised. The Heshima theory of change model was developed (focused on meso and macro levels of the system)	

Interviews and focus gropus	No	Women describe: their negative experiences of childbirth; frustration with lack of confidentiality and autonomy; abandonment by the providers, and dirty maternity units. Providers admit to challenges but describe reasons for apparent abuse (eg slapping on thighs to encourage women to focus on birthing process) and 'detention' is because relatives have abandoned them. Men try to overcome challenges by paying providers to ensure they look after their wives. Drivers of mistreatment are perpetuated by social and gender norms at family and community levels. At facility level, poor managerial oversight, provider demotivation, and lack of equipment and supplies.	Stigma and discrimination (age, young/old; high parity; socio economics); structural gender inequality (social and gender norms at family and community level); power imbalance (women/staff and staff/staff), 'structural disrespect' (poor facilites, lack of resources, staff maltreated, poor management and supervision)	Victim blaming, shaming	Structural disrespect' - that is that poor facilites and resources are also a marker of systemic disrespect, and these may engender abusive behaviours - if staff don't feel valued and respected they cant do a good job, clinically or psychologically/emotionally

Survey and interviews	Normative conduct theory.<br><br>Background notes importance of health systems factors, power differentials, gender based violence (against women);	Participants reported that it was normative in their setting to use abusive and disrespectful behaviour toward women, particularly yelling, insulting, and displaying a hostile or aggressive attitude. However, auxiliary midwives also stressed the importance of making women feel welcome and comfortable, sweet-talking to them throughout labor, and	Not identified	Structural gender violence; Strengths-based approach	

Authors	Year of publication	Title	Aim of study	Review (yes / no)	Type of review	Country	Study context - maternity care, elderly population,	Type of Mistreatment (physical, verbal, physical restraint)	Theoretical perspective (i.e. constructivist, feminist) to undertake study
Asmaningrum, N. and Kurniawati, D. and Tsai, Y. F.	2020	Threats to patient dignity in clinical care settings: A qualitative comparison of Indonesian nurses and patients	To explore and compare nurses' and patients' viewpoints of disrespectful behaviours that threaten patient dignity during hospitalised care.	No	n/a	Indonesia	Hospital - Medical and surgical wards	phsycial and verbal	not identified
Attar-Schwartz, Shalhevet	2011	Maltreatment by Staff in Residential Care Facilities: The Adolescents' Perspectives	This study examines the prevalence and multilevel correlates of verbal and physical mal-treatment of 1,324 Israeli adolescents by staff in 32 residential care settings	No	n/a	Israel	Residential care	Verbal and physical	ecological-integrative model

Ayhan, Cemile Hurrem Balik and Bilgin, HÃ¼lya and Uluman, Ozgu Tekin and Sukut, Ozge and Yilmaz, Sevil and Buzlu, Sevim	2020	A Systematic Review of the Discrimination Against Sexual and Gender Minority in Health Care Settings	To determine discrimination experiences of sexual and gender minority (SGM) individuals and attitudes toward SGM among health care staff in health care settings	Yes	Doesn't specify - just systematic - with results written by descriptively	N/A	Healthcare	Relates to exploring discrimination (so more verbal)	not identified

Baker, P. R. and Francis, D. P. and Hairi, N. N. and Othman, S. and Choo, W. Y.	2016	Interventions for preventing abuse in the elderly	assess the effectiveness of primary, secondary and tertiary intervention programmes used to reduceor prevent abuse of the elderly in their own home, in organisational or institutional and community settings	yes	quant	various	elderly	all	not identified
Braaten, K. L. and Malmedal, W.	2017	Preventing physical abuse of nursing home residents- as seen from the nursing staff's perspective	capture first- hand information from nursing home staff's own understanding regarding what they think and have experienced about prevention of physical abuse of nursing home residents and what measures they consider useful to implement in their daily	No	n/a	Norway	elderly population	physical	not identified

Braun, Kathryn L. and Suzuki, Kathy M.	1997	Developing and testing training materials on elder abuse and neglect for nurse aides	to report on the development of training resources to prevent mistreatment	no	n/a	Hawaii	elderly population	not specifically mentioned - just abuse, infers physical abuse	not identified
Buzgová, R. and IvanovÃ¡, K.	2009	Elder abuse and mistreatment in residential settings	The aim of this study was to describe employees’ and clients’ livedexperiences of elder abuse.	no	n/a	Czech Republic	elderly population	physical, verbal	not identified
Cambridge, Paul	1999	The First Hit: a case study of the physical abuse of people with learning disabilities and challenging behaviours in a residential service	To describe the circumstances surrounding the physical abuse of persons with learning disabilities and challenging behaviours in a residential service	No	n/a	UK	learnig disability, residential setting	physical	Multiple theories, including Goffman, Foucaut ((total) 'institution theory'); disabilitist theory (Wolfensberger); normalisation theory, some allusion to neoliberal approaches to funding for social care. To address the issues: empowerment

Castro, A. and Savage, V. and Kaufman, H.	2015	Assessing equitable care for Indigenous and Afrodescendant women in Latin America	To identify and understand the barriers to equitable care within health care set-tings that women of ethnic minorities encounter in Latin America and to examine possible strategies for mitigating the issues.	Yes	structured	Latin America	Indigenous and ethnic minority women using health care	All	not identified
Ceron, A. and Ruano, A. L. and Sanchez, S. and Chew, A. S. and Diaz, D. and Hern and ez, A. and Flores, W.	2016	Abuse and discrimination towards indigenous people in public health care facilities: experiences from rural Guatemala	to understand and categorizethe episodes of discrimination as reported by indigenous communities seeking health care in rural Guatemala	No	n/a	Guatemala	Indigenous people using public health facilities	All	not identified
Conner, T. and Prokhorov, A. and Page, C. and Fang, Y. and Xiao, Y. and Post, L. A.	2011	Impairment and abuse of elderly by staff in long-term care in Michigan: evidence from structural equation modeling	To place known factors associated with elder abuse in a causal structure that relates the factors to each other and to whether abuse occurs.	No	n/a	USA	Elderly	physical	Positivist (implied)
Cooper, C. and Dow, B. and Hay, S. and Livingston, D. and Livingston, G.	2013	Care workers' abusive behavior to residents in care homes: a qualitative study of types of abuse, barriers, and facilitators to good care and development of an instrument for reporting of abuse anonymously	development of a questionnaire to report abuse	no	n/a	UK	elderly population	physical, verbal, physical restraint	not identified

Corbi, G. and Grattagliano, I. and Ivshina, E. and Ferrara, N. and Solimeno Cipriano, A. and Campobasso, C. P.	2015	Elderly abuse: risk factors and nursing role	to examine the available literature in the last 5 years to define the state of art on thisphenomenon, with particular regard to the nursing role inelderly abuse, focusing on the possible types of mistreat-ment, the motivations and preventive interventions.	yes	narrative	various	elderly population	abuse generally mentioned	not identified
Eren, N.	2014	Nurses' attitudes toward ethical issues in psychiatric inpatient settings	to evaluate the ethical beliefs of psychiatric nurses and ethical problemsencountered.	no	n/a	Turkey	psychiartric inpatients	verbal abuse and physical restraint	not identified

Frazao, S. L. and Correia, A. M. and Norton, P. and Magalhaes, T.	2015	Physical abuse against elderly persons in institutional settings	to provide a better knowledge about physical abuse againstelderly people in institutional settings, in order to contribute to a timely detection, correct forensicdiagnosis and prevention of these cases.	No	n/a	Portugal	elderley	physical, neglect	not identified

Gil, Ana Paula	2019	Quality procedures and complaints: nursing homes in Portugal	analyse how mistreatment of older people is identified and dealt with by the national social security services. In particular it looks at what the indicatorsare with which to assess poor quality care and mistreatment (how it is perceived and defined), which factors affect mistreatment of older people and intervention outputs (i.e. what are the	no	n/a	Portugal	elderly population	physical and verbal abuse	not identified
Hassouneh-Phillips, D. and McNeff, E. and Powers, L. and Curry, M. A.	2005	Invalidation: a central process underlying maltreatment of women with disabilities	understand factors underlying mistreatment of women with disabilities by healthcare staff	No	n/a	US	women with disablities	Physical	not identified
Hyde, Paula and Burns, Diane and Killett, Anne and Kenkmann, Andrea and Pol and , Fiona and Gray, Richard	2014	Organisational aspects of elder mistreatment in long term care	examined the organisational factors associated with abuse, neglect and/or loss of dignity of older people resident in care homes.	yes	evidence synthesis		elderly population	abuse generally mentioned	not identified

Lim, J.	2020	Factors Affecting Mistreatment of the Elderly in Long-Term Care Facilities	explore the factors affecting elderly mistreatment by care workers in Japaneselong-term care facilities and to examine the relationship between these factors and mistreatment.	no	n/a	Japan	elderly population	just mistreatment mentioned, not specific	not identified
Mercier, E. and Nadeau, A. and Brousseau, A. A. and Emond, M. and Lowthian, J. and Berthelot, S. and Costa, A. P. and Mowbray, F. and Melady, D. and Yadav, K. and Nickel, C. and Cameron, P. A.	2020	Elder Abuse in the Out-of-Hospital and Emergency Department Settings: A Scoping Review	synthesize the available evidence on the epidemiology, patient- and caregiver-associated factors, clinicalcharacteristics, screening tools, prevention, interventions, and perspectives of health care professionals in regard to elder abuse inthe out-of-hospital or emergency department (ED) setting.	Yes	scoping	various	elderly population in emergency care	physical	not identified
Moore, Steve	2019	The relativity of theory: applying theories of social psychology to illuminate the causes of the abuse of older people in care homes	the purpose of this paper is to explain the evident continuing abuse of adults at risk living in care homes by the staff who should be looking after them.	Yes	narrative	n/a	elderly population	abuse generally mentioned	not identified
Natan, M. B. and Lowenstein, A.	2010	Feature. Study of factors that affect abuse of older people in nursing homes	to examination the effects of long-term care facility traits on the maltreatment of older people	No	n/a	Isarel	elderly population	physical	not identified

Natan, M. B. and Lowenstein, A. and Eisikovits, Z.	2010	Psycho-social factors affecting elders' maltreatment in long-term care facilities	To examine and analyse major variables affecting maltreatment of elderly nursing home residents.	No	n/a	Isarel	elderly population	maltreatment generally (including physical and verbal abuse)	not identified
Phillips, L. R. and Guo, G.	2011	Mistreatment in assisted living facilities: complaints, substantiations, and risk factors	explore relationships among selected institutional and resident risk and situation-specific factors and complaints and substantiated allegations of various types of mistreatment in assisted living facilities	No	n/a	US	elderly population	Physical, verbal and physical restraint	not identified
Pillemer, K.	1988	Maltreatment of patients in nursing homes: overview and research agenda	critical review of the social science literature regarding maltreatment of patients in nursing homes.	Yes	narrative		elderly population	mistreatment generally	not identified
Pillemer, K. and Moore, D. W.	1990	HIGHLIGHTS FROM A STUDY OF ABUSE OF PATIENTS IN NURSING HOMES	to shed light on the situations in which staff act towards patients in a way that can cause them additional suffering	no	n/a	US	elderly population	physical, physical restraint (excessive)	not identified

Pillemer, Karl and Bachman-Prehn, Ronet	1991	Helping and hurting	identify factors underlying mistreament of elderly patients	No	n/a	US	elderly	physical, physical restraint (excessive use)	not identified
Saveman, B. and Astrom, S. and Bucht, G. and Norberg, A.	1999	Elder abuse in residential settings in Sweden	To investigate frequencies and types of elder abuse occurring in residential settings in two municipal areas of Sweden	no	n/a	Sweden	elderly population	physical	not identified
Schiamberg, L. B. and Oehmke, J. and Zhang, Z. and Barboza, G. E. and Griffore, R. J. and Von Heydrich, L. and Post, L. A. and Weatherill, R. P. and Mastin, T.	2012	Physical abuse of older adults in nursing homes: a random sample survey of adults with an elderly family member in a nursing home	to estimate prevalence of physical abuse in nursing homes and to identify individual and social/contextual risk factors of physical abuse	No	n/a	USA	Elderly care	Physical and neglect	not identified
Silverman, B. C. and Stern, T. W. and Gross, A. F. and Rosenstein, D. L. and Stern, T. A.	1996	Lewd, crude, and rude behavior: the impact of manners and etiquette in the general hospital	to understand the impact of lewd, crude, andrude behaviors in the general hospital and to providea context in which to educate clinicians about themanagement of troublesome behaviors of patients andstaff members.	Yes	Historical - with no detailed methods	n/a	Healthcare	Verbal	not identified

Stevens, M. and Biggs, S. and Dixon, J. and Tinker, A. and Manthorpe, J.	2013	Interactional perspectives on the mistreatment of older and vulnerable people in long-term care settings	developing theoretical and methodologicalunderstanding of the abuse and neglect (mistreatment) of older people in long-term care settings such as care homes and hospitals.	yes	narrative	n/a	elderly population	mistreatment generally	interactionist

Study design (grounded theory/phenomenology/qualitative exploratory, etc)	Participant characteristics - staff/professionals (age, profession,	Participant characteristics - patients/service-users (age, ethnicity, etc)	Data collection methods	Apriori theory used to analyse/frame the data?	Key findings/ Themes	Explicit theories used to interpret findings (includes reference/clearly defined)	Theories/ideologies implicit by authors (but not references/explicity named)	Other interpretations used to explain the findings
qualitative descriptive design	40	35 patients (see Table 1) - 18 males and 17 females	Semi-structured interviews	not identified	Three categories were important for both nurses and patients: negligence, impoliteness and dismissal. Descriptions of the behaviours were comparable for both groups. The forth category, inattentiveness, was highlighted by nurses, while the fifth category, discrimination, was highlighted by patients.	Not identified	not identied	Differences in social roles and responsibilities.
Quantitative/prevalence based study	n/a	1,324 Jewish and Arab adolescents (ages 11–19) residing in residential care	Self-report questionnaire	not identified	24.7% of adolescents report being victims of at least one act of physical maltreatment - 29.1% report at least one act of verbal maltreatment. 29.6% of boys experienced at least one physical maltreatmentact v's 19.2% of girls (statistically significant); 32.9% of girls experienced at least one verbal maltreatment compared to 26.1% of boys. adolescents who perceive theRCS's staff to be more supportive, or to be less strict, are estimated toreport less of each form of maltreatment by staff. Perception of the centre's policy on violence is negatively associated with maltreatment - adolescents with higher scores on the policy index (indicating level of agreement that the residential care setting on violence is clear, fair, and consistent) are estimated to reportlower levels of both physical and verbal maltreatment by staff. Perception of the level of support and strictness found to be associated with both forms of maltreatment. In regression model, individual level factors (age, ethnicity, hyperactivity, etc) explain most variance in adolescents' reports of staff physical maltreatment physical maltreatment; Results also indicate that the largest share of the	Not identified	not identied	Gender (i.e. boys more aggressive); staff feel physical intimidation will be more effective with boys than girls; caregivers lack of understanding; lack of training; association between maltreatmentand the use of authoritarian discipline methods; patriarchal and authoritatian family values, cultural and socioeconomic issues;

Systematic review	14/30 studies conducted with health care workers	16/30 studies undertaken with sexual and gender minority (SGM) individuals - LGBTQ	n/a	not identified	Between 2% and 41.8% experience discriminatory behavior - often related to refusalof needed medication due to sexual orientation and gender; studies. Sexual minority men had more negative experiences of discrimination than the other SGM subgroups; Prevalence ofdisclosure to health care staff in SGM individuals varied from 17% to 75% (due to fear of stigmatization/negative implications of disclosures, e.g. mental health referrals. Gender, religion,occupation,knowledge level, sexual orientation, and education level of healthcare staff wereassociated with negative attitudes about SGM people. Having an SGM [illegible]	Not identified	Homophobia; stigma;	Socio-cultural beliefs

review	n/a	n/a	n/a	ecological perspective	There is inadequate trustworthy evidence to assess the effects of elder abuse interventions on occurrence or recurrence of abuse, althoughthere is some evidence to suggest it may change the combined measure of anxiety and depression of caregivers. There is a need forhigh-quality trials, including from low- or middle-income countries, with adequate statistical power and appropriate study characteristicsto determine whether specific intervention programmes, and which components of these programmes, are effective in preventing orreducing abuse episodes among the elderly. It is uncertain whether the use of educational interventions improves knowledge and attitudeof caregivers, and whether such programmes also reduce occurrence of abuse, thus future research is warranted. I	result of complex interactions among factorsat the individual, relationship, community and societal levels,which can be conceptualised using an ecological perspective	not identied	individual factors - patient vulnerablity - dissabilities and chronic health problems that result in increased dependence on caregivers areparticularly at risk of elder maltreatment. lowsocial support, loneliness, social isolation and lack of socialnetworks among the elderly further perpetuate maltreatment. Perpetrators' mental illness,high levels of hostility, substance abuse, psychological distressand their dependence on the victim for accommodation andfinancial support appear to be strong risk factors that predisposeelderly to maltreatment. Womenwere generally
qualitative exploratory	14 nursing home staff	n/a	focus groups	not identified	several factors contribute to the prevention of physical abuse of residents in nursing homes. There is a requirement for increased competence among staff about the concept of abuse and known risk factors. Good communication skills and trusting relationships are important factors, as well as a culture that fosters openness where ethical dilemmas can	Not identified	not identied	difficulties in defining abuse and knowing when crossed a boundary

development of training	35 people working in nursing homes, 5 administrators, 4 social	n/a	interviews	not identified	participants gave the training high ratings and demonstrated a significant increase in job satisifcation pre to post training.	Not identified	poor working conditions, lack of knowledge/strategies	working conditions (short staffing) and lack of knowledge about dealing with conflicts and difficulties
qualitative phenomenological method	26 employees from four residential homes, 2 managers outside these insitutions	20 elderly residents from the 4 residential homes	interviews	not identified	Established forms of elder abuse were summarized as rights violation, financial abuse, psychological abuse, physica labuse and neglect. Causes of elder abuse included institution, employee and client char-acteristics.	Not identified	not identied	insititutional charactersitics (poor organisation, reigmen, staff shortages), employee charactersitics (burnout, personal probs, inadequate
case study of an inquiry into community residential home abuse		minority ethnic people, one male one female, severe learning difficulites and challenging behaviours	Not applicable: secondary analysis of a public inquiry	See under theoretical perspective	Components of a culture of abuse: isolation of the specific service from the overall organisation ('corruption of care');ineffective staff supervision (and failure to discipline a powerful lead perpetrator); Intimidation (by the powerful perpetrator and their 'in' colleagues);institutionalisation (of violence); inexperience (and powerlessness of new staff to challenge); anti-professionalism (explicit flouting of guidelines). Barriers to disclosure at multiple levels. Poor support for whistle blowers. Deficiencies in Service Audit.Poor interprofessoinal communication. Poor recognition skills. Lack of clarity in case management. Deficiencies in market management and service specification.	Prior cited theories all implied in the analysis and discussion: Goffman and Foucault are cited in the thematic interpretation	not identied	Author states: 'The abuse of people with learning difficulties… will never be eradicated in a society which institutionalises human and other caital in services and where dependity relationshs themselves become a means of economic production. The professionalisation of care also risks hierarchies of power and status which provide opporunties

structured review	n/a	n/a	n/a	Broadly (notions of institutional discrimination and power differentials between providers and service users are in the	Discrimination in the health care setting as a public health issue; Health outcomes and discrimination in health facilities; Reducing discrimination in health care	Not identified	Disrimination at the structural and societal level, and gender discrimination, as well as discriminatoiat the provider level, are all discussed	Authors hypothesise that only interventions that operate at all these levels are likely to be successful to reduce discrimination and subsequent abuse
Qualitative descriptive	n/a	Both male and female participants - no other demographics given	Focus groups	broadly: notoins of discrimination on the gounds of ethnicity, poverty, language, labelling of	three themes: Theme 1: Discrimination in access to care; Theme 2: Abusive treatment in care; Theme 3; neglect of professional ethics	Not identified	not identied	Discrimination' and power differntials discussed but not linked to any more explict theories
Quantiative observational study	n/a	769 of the persons in long-term care were aged 65 or older.	Postal survey	None (though hypotheses are stated linking different factors to the hypothesised impact on experience of abuse)	A good fit is claimed for the model, but the relationships it illustrates (between physical impairment, congitive impairment, behavioual problems, age and abuse) are not straightforward	Not identified	? A deficit model - that the more physically impaired and behaviourally challenging an elderly person is the more likely they are to stress carers, and	not identified
development of questionaire	36 care workers from 4 London care homes	n/a	focus groups	not identified	situations with potentially abusive consequences were a commonoccurrence, but deliberate abuse was rare. Residents waited too long for personal care, or were denied carethey needed to ensure they had enough to eat, were moved safely, or were not emotionally neglected. Somecare workers acted in potentially abusive ways because they did not know of a better strategy or understandthe resident’s illness; care workers made threats to coerce residents to accept care, or restrained	Not identified	not identied	institutional factors (Bureaucracy, lack of information about residents), care worker conditions (dispowerment, lacking training and knowledge, ignoring residents personal needs to avoid work), resident factors

review	n/a	n/a	n/a	not identified	elderly abuse in nursing homes is still underreported in both ori-ginal articles and reviews. Despite the aging of the popu-lation, elderly abuse and neglect still remain hidden problems, overlooked and also underestimated in theliterature.	Not identified	not identied	incidence of elder abuse in health centers is usually pro-portional to the number of institutionalized patients fromhome to long-term care hospitals and nursing homes, with,therefore,
cross sectional questionnaire	202 psychiatric nurses from five psychiatric		questionnaire	not identified	nurses needed additional education in psychiatric ethics. Insufficientpersonnel, excessive workload, working conditions, lack of supervision, and in-service training wereidentified as leading to unethical behaviors.	Not identified	not identied	Insufficient personnel, excessive workload, working conditions, lack of supervision, and in-

Review of case notes	n/a	Most alleged victims were female (n¼47, 79.7%), 75 years orolder (n¼41, 75.9%), with a mean age of 79.7 (Min¼66,Max¼107), all retired and mostly without a partner (51.5%widowed or divorced and 39.4% single). The majority presentedsome degree of disability: mild/moderate (n¼22, 37.3%) or severe(n¼33, 55.9%). Twenty-six victims (47.2%) were unable tocommunicate and 1 (1.8%) experienced difficulties in doing so, justbeing able to answer simple	Logging of cases	not identified	A llegation ofphysical abuse appeared isolated in 93.2% of the cases (n¼55) andassociated with neglect in 3.4% (n¼3). The main recognisedmechanism of aggression was grasping (n¼10, 83.3%) and, in 2cases, multiple mechanisms were mentioned (pushing andpunching; slapping and aggression with a scissors)	Due to the interaction among factors of the individual, relationship, community and societal levels, the ecological model is useful for considering risks and understanding the types of programmes that need to be implemented at different levels (Schiamberg et al. (2011):: (micro, meso, macro system factors noted by the authors)	not identied	Largely clinical and descriptive rather than interpretive

qualitative exploratory	focus groups were carried out with inspectorsfro m the National Inspection Service.	n/a	mixed method, using adatabase of 3,685 complaints reported to the social security inspection services.	not identified	Some indicators were found in key areas of care and the factors associated withthis were based on Kamavarapu's typology (2017): physical conditions of facilities; closed organisational models; difficult working conditions; and perceived concerns of residents. Monitoring and inspection systemsare still based on minimum standards focusing on structural and process quality, devoting little attention tothe human rights situation of older persons and clinical issues	Not identified	not identied	physical conditions of facilities; closed organisational models; difficult working conditions; and perceived concerns of residents.
qualitative exploratory	not mentioned	not mentioned	secondary analysis of data derived from three qualitative studies of abuse of women with	Medical model of disablity	Invalidation was found to uderlie maltreatment. Invalidation was characterized by health care pro-viders Taking Overcare, Discounting, Objectifying, and Hurtingwomenwith disabilities during health care encounters.	Medical model of disablity	not identied	not identified
evidence synthesis	care staff and managers of residential care homes	residents, relatives	reivew, documentary analysis (invesigation reports of abuse) and panel meetings	socioecological model	five organisational factors associated with elder mistreatment; infrastructure, management and procedures, staffing, resident population characteristics and culture. It also indicates macro-structural factors affecting care quality. Investigation reports recognised common structural factors contributing to institutional abuse, however, as 'problem' organisations are closed down these circumstances recur elsewhere.	socioecological model, stress	institutional infrastructure, workload pressures	five organisational factors associated with elder mistreatment; infrastructure, management and procedures, staffing, resident population characteristics and culture; commonly-identified problems including an increase in the number of high dependency residents, lack of capability of staff, poor staffing levels,

cross sectional questionnaire	1473 care workers from long-term care facilities	n/a	survey	Schiamberg's ecological model - risk factors for mistreatment in nursing homes include patient traits, staff traits, institutional	nursing care level, work period, resilience,and attitude towards mistreatment among residents and staffwere factors significantly associatedwith the degree of mistreatment. Facility size, an institutional environment that does not limit the behavior of residents, and family and community support for the elderly were among the institutionalenvironment factors that had significant relationships with mistreatment. Staff gender, care-related	Schiamberg's ecological model - risk factors for mistreatment in nursing homes include patient traits, staff traits, institutional environments, and societal factors.	stress and resilence	nursing care level, staff work period, resilience, attitude towards mistreatment, facility size, autonomy at work, institutional care environment that does not limit the behavior of residents,
scoping review	n/a	n/a	n/a	not identified	prevalence of elder abuse reported during the ED visit was lower than reported in thecommunity. The most commonly detected type of elder abuse was neglect, and then physical abuse. The following factors were more common in identified cases of elder abuse: female sex, cognitive impairment, functional disability, frailty, social isolation, and lowersocioeconomic status. Psychiatric and substance use disorders were more common among victims and their caregivers.	Not identified	not identied	patient characteristics: abuse more common in females, those with a higher frailty score, a lower level of education, and psychiatric and drug or alcohol abuse disorders, cognitive impairment and dementia, problem
review	n/a	n/a	n/a	a range of social psychology theories/models		discusses a range of social psychology theory and explores findings using a socioecological framework, proposing that Henri Tajfel's theory on the social psychology of	not identied	not identified
cross sectional questionnaire	staff working at 24 of the 300 long-term facilities for older	n/a	questionnaire	not identified	Slightly more than half of the sample reported abuse of older residents in the previous 12 months, manifesting in one or more types of maltreatment. High staff turnover was associated with a greater risk of mental or	Not identified	not identied	size of the facility, high staff turn over, high staff to patient ratio (this is correct!!)

cross sectional questionnaire	510 staff working in 22 nursing homes	n/a	questionnaire	the theoretical model for predicting causes of maltreatment ofelderly residents developed by Pillemer (1988) - involving the work environment at the facility, patient traitsand staff	Slightly more than half of the staff sampled reported abuse of elderly residents over the past year,as manifested in one or more of types of maltreatment. The total number of various types of maltreatment reported was 513. About two-thirds of the cases were incidents of neglect. Seventy per cent of respondentsreported that they had been present at incidents in which another staff member abused an elderly resident inone or more types of maltreatment, and in such situations mental abuse and mental neglect were the mostprevalent forms of maltreatment.	the theoretical model for predicting causes of maltreatment ofelderly residents developed by Pillemer (1988) - involving the work environment at the facility, patient traitsand staff traits, and the Theory of Reasoned Action developed by Ajzen & Fishbein.	burnout	elder maltreatment is a result of demographic characteristics, staff training features and patient traits affected by the orga-nization's work environment (the facility). women have a highrisk of experiencing physical violence, correlation between the number of beds,number of nurses, number of
secondary data analysis	n/a	n/a	examination of archived public data of complaints and allegations of abuse in assisted living centres	not identified	The complaint group was comprised of significantly more assisted living centers, large facilities (51–101+), facilities licensed to pro-vide personal care services, and facilities owned by national corporations.	Not identified	not identied	size of facility, lack of training, Physical abuse is closely aligned to the power and control issues inherent in other types of
review	n/a	n/a	n/a	sociological theory		proposes a theoretical model involving interactions between nursing home environment and patient and staffing characteristics and exogenous factors impacting on elder mistreatment	not identied	exogenous factors - supply of nursing home beds (e.g. bed shortage) and unemployment rates, nursing home enivironment - custoidal care, size of facility, level of care, rates of care, cost of patient care, staff-patient ratio, staff turnover. Staff
qualitative exploratory	57 nursing homes (survey) - 577 nurses	n/a	focus groups and survey	not identified		Not identified	not identied	staff to patient conflict, burnout, younger staff and staff with negative

cross sectional questionnaire	577 nurses and nursing aids	not mentioned	survey	not identified	three sets of variables were predicted to have physical and psychological abuse - situational, participant and insititutional characteristics - logistic regression revelealed that situational characteristics were best predictors of maltreatment, specifically burnout and high	Not identified	situational charactersitics	burnout, high staff-patient conflict
cross sectional questionnaire	499 residential staff	n/a	questionnaire	not identified	In the specific situations reported, the abusers weremostly characterised as hot-tempered, exhausted, and burned out. Theabused people were often mentallyand/or physically handicapped andgenerally over 80 years old. Feelingssuch as powerlessness, anger to-wards the abuser, and compassion for the abused person were reported	Not identified	Feelings of powerlessness and inadequacy	abusers were most commonly men, aggressive and easilylost their temper, exhausted and burned out, and/or dominant and egoistic. abused persons weremost commonly women had more than one handicap/disability,
Survey	No staff were included	Relatives completed the survey about older family members (>65 years old) in care homes. 452 responses - resident details = 73% female; 91% white; 86% high school or less; 87% married or	Survey	Limited - structured around the potential factors that might increasse the likelihood of mistreatment, e.g. gender, age, institutional environment, degree of	Limitations in activities of daily living (ADLs), older adult behavioral difficulties, and previous victimization by nonstaff perpetrators were associated with a greater likelihood of physical abuse.	Not identified	Mainly focuses on factors associated with mistreatment and the relationship with caregivers...including the nature of wider (distal) relationships between caregivers and elderly relatives.	not identified
States historical but reads more of an opinion piece	n/a	n/a	No details on how data collection	not identified	Details a range of reasons for lewd/crude behaviour in physician-patient and physician-other interactions - lot of focus in on ethics, values, etiquette (including dress and language) rather than verbal abuse per se	Not identified	biopsychosocial context	Lack of respect (for patients), patient intimidation, disordered brain functions/affective dysregulation

review	n/a	n/a	n/a	positioning theory analysis - interactions arebased on taking of 'positions', clusters of rights and duties to act in certain waysand impose particular meanings, which enable or prohibit access	The central argument of the article is that patterns of micro-interactions, particularly positioning older people with care needs as less able,less worthy of interaction and not complete people can lead to andform part of loss of dignity and give rise to risks of mistreatment.Such commonpatterns of positioning and interpretations of language, create and reproduceorganizational culture and societal factors such as ageism and therefore providea valuable insight into how more macro scale characteristics,such as routines ofpractice or management styles and social inequalities, can influence and beinfluenced by micro-interactions that create the backdrop to the	Not identified	not identied	'malignant' positioning can contribute to the creationof a climate that allows mistreatment to take place, or fails to prohibit itsdevelopment. Mistreatment of people with dementia is used as an illustration. Positioning theory also allows for an

Authors	Year of publicatio n	Title	Aim of study	Review (yes / no)	Type of review	Country	Study context - maternity care, elderly population, physical restraint/ment	Type of Mistreatment (physical, verbal, physical restraint)	Theoretical perspective (i.e. constructivist, feminist) to undertake study
Acevedo-Nuevo, M. , Gonzalez-Gil, M. T. , Solis-Munoz, M. , Arias-Rivera, S. , Torano-Olivera, M. J. et al	2020	Physical restraint in critical care units from the experience of doctors and nursing assistants: In search of an interdisciplinary interpretation	aims to approach how professionals other than nurses (doctors and nursing assistants) conceptualise the use of PR in CCU and other associated factors, as well as how their experience influences nurses' decision-making.	no	n/a	Spain	critical care	physical restraint	not identified
Allen, J. J.	2000	Seclusion and restraint of children: a literature review	nurses beliefs about selcusion and restraint of children and what alternatives are avaiable	yes	narrative	various	children's nursin	physical restraint	not identified

Chapman	2015	Australian nurses' perceptions of the use of manual restraint in the Emergency Department: a qualitative perspective	To explore emergency nurses perceptions of the use of manual restraint.	No	n/a	Australia	emergency dept	Physical restraint	not identified
Cui, N. , Long, M. , Zhou, S. , Zhang, T. , He, C. , Gan, X.	2019	Knowledge, Attitudes, and Practices of Chinese Critical Care Nurses Regarding Physical Restraint	to determine the knowledge, attitudes, and practices regarding the use of physical restraints by Chinese nurses in intensive care units (ICUs) of ter-tiary hospitals.	no	n/a	China	critical care	physical restraint	not identified
Dolan, J. , Dolan Looby, S. E.	2017	Determinants of Nurses' Use of Physical Restraints in Surgical Intensive Care Unit Patients	To describe nurses' determinants of initiation and discontinuation of restraints in surgical intensive care unit patients.	no	n/a	US	Intensive care	physical restraint	not identified

Esk,ari, F. , Abdullah, K. L. , Zainal, N. Z. , Wong, L. P.	2018	The effect of educational intervention on nurses' knowledge, attitude, intention, practice and incidence rate of physical restraint use	evaluate the effect of educational intervention on nurses' knowledge, attitude, intention, practice and incidence rate of physical restraint in 12 wards of a hospital [illegible]	no	n/a	Malaysia	inpatient wards	physical restraint	not identified
Hamilton, Deborah , Griesdale, Donald , Mion, Lorraine C.	2017	The prevalence and incidence of restraint use in a Canadian adult intensive care unit: A prospective cohort study	To determine the extent of physical restraint use in anurban teaching ICU, to identify patient-specific factors that maycontribute to the application of physical restraints, and explorenurses' rationale for use.	no	n/a	Canada	intensive care	physical restraint	not identified
Lach, Helen W.	2016	Changing the Practice of Physical Restraint Use in Acute Care	evaluate current evidence on use of physical restraint in acute care	yes	narrative	various	acute care	physical restraint	not identified

Lei, R. , Jiang, X. , Liu, Q. , He, H.	2020	Nurse education to reduce physical restraints use in ICU: A scoping review	To identify and map nurse education strategies that reduce the use of physicalrestraints in the ICU.	yes	scoping	various	Intensive care	physical restraint	not identified
Martin, B. , Mathisen, L.	2005	Use of physical restraints in adult critical care: a bicultural study	To describe the relationship between patients' characteristics, environment, and use ofphysical restraints in the United States and [illegible]	no	n/a	US/Norway	critical care	physical restraint	not identified
Mitchell, D. A. , Panchisin, T. , Seckel, M. A.	2018	Reducing Use of Restraints in Intensive Care Units: A Quality Improvement Project	To reduce and sustain the restraint rates to less than the national database mean rates for all 5 intensive care units.	no	n/a	US	intensive care	physical restraint	not identified

Paterson, Brodie	2011	How corrupted cultures lead to abuse of restraint interventions	to explain why corrupt cultures result in abuse of physical restraint	yes	narrative	n/a	learning difficult	physical restraint	not identified
Perez, D. , Peters, K. , Wilkes, L. , Murphy, G.	2019	Physical restraints in intensive care-An integrative review	to explore the current literature on the use of PR inintensive care.	yes	mixed	various	intensive care	physical restraint	not identified
Rainier, N. C.	2014	Reducing physical restraint use in alcohol withdrawal patients: a literature review	to see what, if any, alternatives have been tested to improvepatient care for this complicated patient population.	yes	narrative	n/a	acohol withdrawal patients	physical restraint	not identified

Unoki, T. , Hamamoto, M. , Sakuramoto, H. , Shirasaka, M. , Moriyasu, M. , Zeng, H. , Fujitani, S.	2020	Influence of mutual support and a culture of blame among staff in acute care units on the frequency of physical restraint use in patients undergoing mechanical ventilation	verify the hypothesis that mutual support and a culture of blameamong staff are associated with higher physical restraint use for mechanically ventilated patients.	no	n/a	Japan	Intensive care	physical restraint	not identified

Via-Clavero, G. , Guardia-Olmos, J. , Falco-Pegueroles, A. , Gil-Castillejos, D. , Lobo-Civico, A. , De La Cueva-Ariza, L. , Romero-Garcia, M. , Delgado-Hito, P.	2020	Factors influencing critical care nurses' intentions to use physical restraints adopting the theory of planned behaviour: A cross-sectional multicentre study	determine critical care nurses' attitudes, subjective norms,perceived behavioural control, and intentions to use physical restraints in intubated patients and the relationship between them and sociodemographic, professional, and contextual factors using a survey approach.	no	n/a	Spain	critical care	physical restraint	not identified

Study design (grounded theory/phenomenology/qualitative exploratory,	Participant characteristics - staff/professionals (age, profession, ethnicity, etc)	Participant characteristics - patients/service-users (age, ethnicity, etc)	Data collection methods	Apriori theory used to analyse/frame the data?	Key findings/ Themes	Explicit theories used to interpret findings (includes reference/clearl	Theories/ideologies implicit by authors (but not references/explicity named)	Other interpretations used to explain the findings
qualitative phenomenological interpretive	18 professionals (doctors and nursing assistants)	n/a	focus groups	theory of planned behaviour, social influence	The real reduction in the use of physical restraint in CCU must be based on one crucial point: acceptance of the complexity of the phenomenon. The use of physical restraint observed in the different CCU is influenced by individual, group and organisational factors. These factors will determine how doctors and nursing assistants interpret safety and risk, the centre of care (patient or professional-centred care), the concept of restraint,	not identified	not identified	risk management - safety of patient and staff
review	n/a	n/a	n/a	not identified	little known about the selusion and restraint of children in nursing	not identified	organisational culture, authoritarianism	safety, appropriate response to physical aggression from a patient

qualitative exploratory	n/a	15 emergency nurses	semi-structured interviews	not identified	themes were identified: ‘part of the job’, ‘reasons for manual restraint’, ‘restraint techniques’, ‘consequences’ and ‘lack of documentation’.	not identified	organisational norms (accepted practice),	considered necessary to deal with aggressive and violent behaviour from patients, or here [illegible]
Cross sectional	383 ICU nurses	n/a	survey	not identified	nurses employed in the ICUs of tertiary hospitals in Chongqing, China, have relatively insufficient knowledge, comparatively incorrect attitudes, and unreasonable means of practicing physical restraint. Continuing education should focus strongly on the relationship between physical restraint and unplanned	not identified	not identified	knowledge about physical restraint was inadequate, which might cause inappropriate at-titudes and unsuitable practice. The use of physical re-straint is
qualitative expl	13 nurses	n/a	semi-structured interviews	not identified	Content analysis revealed 3 general categories and 8 themes that indicated the thoughtful reflection processes nurses in a surgical intensive care unit use to determine use of restraints. Top priorities were ensuring patient safety and comfort. Nurses synthesized factors including practice experience, patient-specific behaviors and	not identified	not identified	patient safety, patient behaviour (functional ability)

A quasi-experimental pre-post design	245 nurses	n/a	survey	not identified	There was a statis-tically significant decrease in the incidence rate of physical restraint use in the wards of the hospital exceptgeriatric-rehabilitation wards after intervention.	not identified	not identified	nurses lack of knowledge and perception of restraint as nurses perceivedphysical restraint in terms of a [illegible]
mixed methods	30 permanent ICU [illegible]	122 patients aged 18 years and over within 12-24 hours of admission to the ICU.	mixed methods (patient data and semistructured interviews)	not identified	The presence of the endotracheal tube and the administration of any opioidor midazolam increased the odds of physical restraint applica-tion. Nurses did not consult with physicians before restraining the patients. The primary reason for restraining the patient was topreserve the [illegible]	not identified	not identified	for patient safety to stop interference with tubes - not able to trust the patient to not interfere with tubes when at the bedside of another patient, [illegible]
review	n/a	n/a	n/a	not identified	Nurses play a signifi-cant role in the use of restraints. Fac-tors such as nurse's knowledge and patient characteristics combined with the culture and resources in health care facilities influence the prac-tice of physical restraint use. Nurses can identify patients at high risk for restraint use; assess the potential causes of unsafe behaviors; and tar-get interventions in the areas of phys-iological, psychological,	not identified	not identified	Prevention of falls is a primary reason for re-straint use on medical–surgical units, whereas preventing removal of medical devices and confusion are primary reasons in

review	n/a	n/a	n/a	not identified	There are a variety of nurse education activities that could effectivelyimprove ICU nurses' knowledge, attitudes, and behaviours towards physicalrestraints. However, the strategies of nurse education, such as delivery mode, teach-ing methods, and outcome measurements, require further study.	not identified	not identified	nurses had inadequate overall knowledge of physical restraint use, and even when a patient was judged not to need physical restraints, nurses may still apply it as a safety consideration. However, we found a limitednumber of studies—only
descriptive correlational	n/a	50 patients in each country	observations of patients, chart data	not identified	Critical care units with similar technology and characteristics of patients vary between nations in restraint practices, levels of sedation, and nurse-to-patient ratios. Restraint-free care was, in this sample, safe in [illegible]	not identified	not identified	institutional nor[illegible]
case stuy	n/a	n/a	secondary data analysis and survey	patient safety	All 5 intensive care units were able to successfully decrease restraint rates to less than the national database mean for the majority of the months since the start of the restraint [illegible]	not identified	not identified	patient safety, prevent extubation, lack of alternatives

review	n/a	n/a	n/a	not identified	In a corrupted culture, the needs of the service user become secondary to the needs of staff, who have become demoralised without adequate training and support. Leadership is seen as critical in the reduction of coercive interventions, but effective staff training can bring	not identified	corruption, labelling (distancing from clients)	work routines and practices that depersonalise and dehumanise service users and staff, weak local management
review	n/a	n/a	n/a	not identified	Five major themes emerged: (i) prevention of treatment interference; (ii) nurses' role as primary decision-makers PR application and removal; (iii) adherence to PR protocols; (iv) moral and ethical dilemmas faced bynurses; and (v) experiences of patients and families.	not identified	theory of planned behaviour - intention influenced by workload pressures	prevent self-extubation, nurses as decision makers - nurses' perception of patient harm and workload pressures profoundly influenced their role as primary decision-makers in this context
review	n/a	n/a	n/a	not identified	Despite the wealth of research on both alcohol withdrawaland physical restraint, there is little research that exploresthe relationship between the two. he only evidenced-based intervention at this time is proper pharmacologicalmanagement and restraint education. Education should befocused on	not identified	not identified	Clinical justifications for use include prevention ofdelayed delivery in care resulting from dislodged lines andtubes and ensuring patient safety, estrained

cross sectional s	333 nurses in intensive care units caring for mechanically ventilated patients in acute care units.	n/a	survey	organisational norms - culture of blame	The mean number of beds per nurse was not significantly different between the groups; the mean and percentage of positive responses about mutual support and a culture of blame were significantly lower in the high frequency physical restraint use group. After adjusting variables in a multivariable regression analysis, a less positive response about the culture of blame was the only independent factor to predict high frequency physical	not identified	blame culture, lack of mutal support	

cross sectional s	354 ICU nurses from 2 ICUs (eight general ICUs, two cardiologicalICUs, one surgical ICU, and one burn ICU) from eight acute carehospitals (five public and three private) in three regions in Spain,	n/a	survey	theory of planned behaviour	Critical care nurses' intentions to use physical restraints are moderate and are influenced byintrapersonal, patient, and contextual factors. Nurses who work in units with organisational policies andalternatives to restraints demonstrated lower levels of intention to use them.	theory of planned behaviour	not identified	attitudes - nurses agreed on the hazards of using restraints, their confidence in physical restraints as safety equipment to avoid unplanned removal of life-support devices and to provide professional safety for nurses remained high. Subjective norms - nurses did not perceive that othersexpected

ns - patient safety

Authors	Year of publication	Title	Aim of study	Review (yes / no)	Type of review	Intervention study (YES/NO)	If intervention study - name of intervention (if	Details of intervention	Country	Study context - maternity care, elderly population, physical restraint/mental health	Type of Mistreatment (physical, verbal, physical restraint)	Theoretical perspective (i.e. constructivist, feminist)	Study design (grounded theory/phenomenology/q	Year when data was collected	Sample strategy
Anderson	2003	Nursing Homes as Complex Adaptive Systems: Relationship between Management Practice and Resident Outcomes	To test complexity science hypotheses about the relationship between management practices (communication openness, participation in	No	n/a				US	eldery population	Physical restraint	not identified	cross sectional/ correlation		
Anderson, K. and Bird, M. and MacPherson, S. and Blair, A.	2016	How do staff influence the quality of long-term dementia care and the lives of residents? A systematic	our aim was to answer the following research question: Is it possible to determine which aspects of residential care	yes	mixed				various	elderly population	physical restraint	not identified	Mixed methods review		
Varon, M. and Tabak, N.	1996	Restraining patients as part of hospital policy	examine the use of patient restraints in geriatric institutions.	no	n/a				not specified	eldery population	Physical restraint	not identified	cross sectional survey		
Castle, N. G.	2000	Differences in nursing homes with increasing and decreasing use of physical	examines the organizational characteristics of nursing homes associated with	no	n/a				US	elderly	physical restraint	not identif	cross sectional		

Hamers, J. P. and Meyer, G. and Kopke, S. and Lindenma	2009	Attitudes of Dutch, German and Swiss nursing staff towards physical restraint use in nursing home	To investigate the attitudes of nursing staff towards restraint measures and restraint use in nursing home	no	n/a				The Netherlands, Germany and Switzerland	elderly	Physical re	not identif	cross sectional survey		
Gallinagh, R. and Nevin, R. and Mc Ilroy, D. and Mitchell, F. and Campbell, L. and	2002	The use of physical restraints as a safety measure in the care of older people in four rehabilitation wards: findings from an	to investigate the prevalence and type of physical restraint used with older persons on four rehabilitation wards inNorthern Ireland.	no	n/a				Northern Ireland	elderly	Physical restraint	not identif	observational		
Gunawardena, R. and Smithard, D. G.	2019	The Attitudes Towards the Use of Restraint and Restrictive Intervention Amongst	review of literature about The Attitudes Towards the Use of Restraint andRestrictive	yes	narrative				n/a	elderly population	Physical restraint	not identif	review		
Huang, H. C. and Huang, Y. T. and Lin, K. C. and Kuo, Y. F.	2014	Risk factors associated with physical restraints in residential aged care facilities: a community-based	To identify the rate and risk factors of physical restraint in residential agedcare facilities in Taiwan.	no	n/a				Taiwan	elderly population	Physical restraint	not identif	cross sectional survey		

Andrews, G. J. and Peter, E.	2006	Moral geographies of restraint in nursing homes	to examine the occurance of PR in nursing homes using moral geographies	yes	narrative				n/a	elderly population	Physical restraint	not identif	review		
Backhaus, R. and Verbeek, H. and van	2014	Nurse staffing impact on quality of care in nursing homes: a systematic	summarizes thefindings from recent longitudinal studies about quality of care in	yes	quantitati ve				various	elderly population	Physical restraint	not identif	review		
Blakeslee, J. A. and Goldman, B. D. and	1991	Making the transition to restraint-free care	review of literature on restrain in nursing homes proposing a restraint-free care	yes	narrative				n/a	elderly population	Physical restraint	not identif	review		
Brower, H. T.	1991	The alternatives to	review of the literature to introduce alternatives to PR	yes	narrative				n/a	elderly population	Physical restraint	not identif	review		
Brugnolli, A. and Canzan, F. and Mortari, L. and Saiani, L. and	2020	The Effectiveness of Educational Training or Multicomponent Programs to Prevent the Use of Physical	assesses the effectiveness of interventions to reduce physical restraint (PR)use in older people living in nursing homes or	yes	meta-analysis				various	elderly population	physical restraint	not identif	review		

Caprio, T. V. and Katz, P. R. and Karuza, J.	2008	Commentary: The physician's role in nursing home quality of care: focus on restraints	how the physician's role fits into this quality of care equation for nursing homesand its implications for	yes	narrative				n/a	elderly population	physical restraint	not identif	review		
Cassie, K. M. and Cassie, W.	2013	Racial disparities in the use of physical restraints in U.s.	to determine ifblack residents were more susceptible to the	no	n/a				US	elderly population	Physical restraint	not identif	cross sectional survey		
Castle, Nicholas G. and Banaszak -Holl, Jane	2003	The Effect of Administrative Resources on Care in Nursing Homes	evaluates whether the hours spent on the job by nursing home administration havean effect on the quality of care	no	N/a				US	elderly population	Physical restraint	not identif	secondary data analysis		
Castle, N. G. and Fogel, B.	1998	Characteristics of nursing homes that are restraint free	examine differences between restraint free and non-restraint free nursing homes	no	n/a				US	elderly population	Physical restraint	not identif	secondary data analysis		
Castle, N. G. and Fogel, B. and Mor, V.	1997	Risk factors for physical restraint use in nursing homes: pre- and post-implementation of the Nursing Home Reform Act	(1) to identify resident and facility risk factors for the use ofphysical restraints since the implementation of the Nursing	no	n/a				US	elderly population	Physical restraint	not identif	secondary data analysis		

Darcy, L.	2007	Reducing and/or minimising physical restraint in a	describe a a journey of implementing evidence-based	no	n/a				australia	elderly	physical restraint	not identif	case study	
Goethals, S. and Dierckx de Casterle, B. and Gastman	2013	Nurses' ethical reasoning in cases of physical restraint in acute elderly care: a qualitative study	to explore the ethical reasoningprocess of nurses in cases of physical restraint in acuteelderly care.	no	n/a				Belgium	elderly acute care	Physical restraint	not identif	grounded theory	
Gordon, S. E. and Dufour, A. B. and Monti, S. M. and Mattison,	2016	Impact of a Videoconference Educational Intervention on Physical Restraint and Antipsychotic	Evaluate the use of ECHO-AGE (videoconference sessions between frontline nursing home staff and clinical experts at	no	n/a				US (Massach usetts and Maine)	elderly residential care	physical restraint	not identif	secondary data analysis	
Hamers, J. P. and Bleijleven s, M. H. and Gulpers, M. J. and Verbeek, H.	2009	Behind Closed Doors: Involuntary Treatment in Care of Persons with Cognitive Impairment at Home in the Netherlands	To investigate the attitudes of nursing staff towards restraint measures and restraint use in nursing home residents,and to investigate if these attitudes are influenced by country of residence and individual characteristics of	no	n/a				The Netherlan ds, Germany, Switzerla nd	health care for eldery in a home setting	physical restraint	not identif	cross sectional questionnaire	
Hantikain en, V.	1998	Physical restraint: a descriptive study in Swiss nursing homes	to explore the the reasons for using physical restraints, their prevalence andnurses'	no	n/a				Switzerlan	elderly population	physical restraint	not identif	cross sectional questionnaire	

Hantikainen, V. and Kappeli, S.	2000	Using restraint with nursing home residents: a qualitative study of nursing staff perceptions and decision-making	to explore nursing staff members' percep-tions of restraint and how these perceptions governdecision-	no	n/a				Switzerlan	elderly population	physical restraint	not identif	phenomenology		
Hardin, S. B. and Magee, R. and Stratmann, D. and	1994	Extended care and nursing home staff attitudes toward restraints. Moderately	staff attitudes towards restraint	no	n/a				US	elderly population	physical restraint	not identif	cross sectional questionnaire		
Harding, G. and King, L.	2005	Adhering to the principles of restraint free environments in	summarise the reasons behind continued use of physical restraints	yes	narrative				various	elderly population	physical restraint	not identif	review		
Heeren, P. and Van de Water, G. and De Paepe, L. and	2014	Staffing levels and the use of physical restraints in nursing homes: a multicenter study	examine the relation between ward staffing levels (e.g. staff intensity and staff mix) and the use of physical	no	n/a				Belguim	elderly population	physical restraint	not identif	cross sectional survey		
Heinze, C. and Dassen, T. and Grittner, U.	2012	Use of physical restraints in nursing homes and hospitals and related factors: a cross-sectional study	to investigate factors related to the use of restraints and to explore whether the rate of nurses was an influencing factor regarding the use of restraints in	no	n/a				germany	elderly population	physical restraint	not identif	secondary data analysis		
Hennessy, C. H. and McNeely, E. A. and	1997	Perceptions of physical restraint use and barriers to restraint	examine administrators and nursing staff views of restraints andperceptions of	no	n/a				US	elderly population	physical restraint	not identif	qualitative exploratory		

Mion, L. C. and S and hu, S. K. and Khan, R. H. and Ludwick, R. and	2010	Effect of situational and clinical variables on the likelihood of physicians ordering physical restraints	To model clinical and situational variablesthat may affect likelihood of physicians to order physicalrestraints.	no	n/a				US	elderly population	physical restraint	not identif	cross sectional questionnaire		
Mohler, R. and Richter, T. and Kopke, S. and Meyer, G.	2012	Interventions for preventing and reducing the use of physical restraints in long-term geriatric care - a Cochrane review	To evaluate the effectiveness of interventions to prevent and reduce the use of physical restraints in olderpeople requiring long-term nursing care.	yes	quantitative				various	elderly	Physical re	not identif	review		
Mohler, R. and Richter, T. and	2011	Interventions for preventing and reducing the use of physical	same as Mohler 2012 (one is a cocrane review report)												
Nay, R. and Koch, S.	2006	Overcoming restraint use: examining barriers in Australian aged care facilities	to discuss the outcomes of a project that sought to identify alternatives to restraint use in	no	n/a				Australia	elderly population	physical restraint	not identif	qualitative exploratory		

Sullivan-Marx, E. M. and Strumpf, N. E. and Evans, L. K. and Baumgarten, M. and	1999	Predictors of continued physical restraint use in nursing home residents following restraint reduction efforts	identify resident characteristics and environmental factors associated with initiation of physical restraint.	no	n/a				US	elderly population	physical restraint	not identif	secondary data analysis		
Hantikainen, V.	2001	Nursing staff perceptions of the behaviour of older nursing home residents and decision making on restraint use: a	examined staff perceptions of the behaviour of older nursing homeresidents and how these perceptions govern their	no	n/a				Switzerla	elderly population	physical restraint	not identif	phenomenology		
Buzgova, R. and Ivanova, K.	2011	Violation of ethical principles in institutional care for older people	This study focuses on issues of elder abuse in residential settings	no	n/a				Czech Republic	Elderly population	Verbal and physical	Ethical principles	qualitative exploratory		
Ben Natan, M. and Akrish,	2010	Physically restraining elder residents of long-term care	to identify and analyse major variables affecting intended	no	n/a				Isarel	elderly population	physical restraint	not identified	cross sectional survey		
Bourbonniere, M. and Strumpf, N. E. and Evans, L.	2003	Organizational characteristics and restraint use for hospitalized nursing home residents	To examine the effect of organizationalcharacteristics on physical restraint use for	no	n/a				US	elderly population	physical restraint	not identified	secondary data analysis		

Lai, C. K. Y. and Kong, S. K. F. and Chow, S.	2003	A restraint reduction program in a local old age home	to investigate whether a staff educational program and the establishment of a	no	n/a				Hong Kong	elderly population	physical restraint	not identified	secondary data analysis					

Sample size	Inclusion criteria	Exclusion criteria	Participant characteristics - staff/professionals (age,	Participant characteristics - patients/service-users	Ethical approval/ ethical issues well considered?	Data collection methods	Data analysis methods	Apriori theory used to analyse/frame the data?	Evidence of effect of the intervention	Key findings/ Themes	Explicit theories used to interpret findings (includes	Theories /ideologies implicit by authors (but not	Other interpretations used to explain the findings
			n/a	nursing and registered nurses		survey data from staff and seconfary data analysis from clinical reports		Complexity theory		management practices that facilitate self-organization contribute to	Complexity theory, theory of self-organisation (practice that changes	organisational culture	greater communication openness and smaller organisational size related to lower
			n/a	n/a		n/a		not identified		When staff treat and interact empathetically andhumanely in	Not identified	staff fearing that residents will fall or be in pain	v. little in this one because the focus of the review is predictor
			8 nursing service administrators	n/a		survey		not identified		procedures and policies regarding restraintv	not identified	not identified	reasons for applying restraints appear
			n/a	n/a		secondary data analysis (survey data)		not identified		that some nursing homes have	not identif	not identif	larger facilities, those with Alzheimer

			convenience sample of nursing staff employed	n/a		survey		not identified		Nursing staff from three European countries	not identif	not identif	PR considered clinically appropriate. Dutch nursing
			n/a	102 patients		longitudinal observation		not identified		Those who were restrained were dependent on nursing care tomeet	not identif	not identif	physical restraint in care plan, patient dependency levels, patient wanderin
			n/a	n/a		n/a				The reasons why restraints are used,	patient saf	Cultural, Maintaining a device in situ, Workload	
			256 primary caregivers (78 nurses and 178 careaides) across	847 residents across 178 residential aged care facilitie		epidemiological survey		theoretical framework suggested based around facility level and indivudal		Of 847 residents, 62% (527) were restrained during the	not identif	patient saf	family signed an agreement for restraint to be used

			n/a	n/a		n/a		moral geographies	Nurses are located centrally in the circuitryof institutional power, constituting one	patient safety, emplaced autonomy,	insitution as a public place, medicalisation,spatial and social limitations		
			n/a	n/a		n/a		not identified	No consistent evidence was	not identif	not identif	staff training and experience	
			n/a	n/a		n/a		not identified	physical restraints in long term care settings	not identif	not identified	overprotective concern for safety	
			n/a	n/a		n/a		not identified	once restraints have been applied,	not identif	patient saf	not identified	
			n/a	n/a		n/a		not identified	This review includes 16 studies in a qualitative	not identif	patient saf	not identified	

			n/a	n/a		n/a		not identified		To achieve effective physician integration to the nursing	not identif	medical model, patient safety, elderly as frail	lack of committed workforce
			n/a	13,507 residents from 1,174		survey		racial disparities		Findingsr evealed that black	racism	not identif	not identified
			n/a	n/a		secondary data analysis - uses data from the 1999 On-		Open-systems theory(Scott, 1981). nursing homes are “work-		the results indicate that the quality indicators are	not identif	organisational culture - inefficent management	number of FTE nursing hours and patient characteri
			n/a	n/a		secondary data analysis - data from the On-line Survey and Certification on of Auto-		not identified		Restraint free nursing homes were found more likely to have residents with	not identif	not identif	Ownership, chain membership, aver- age occupancy rates, facility size, staffing
			n/a	n/a		secondary data analysis - the Health Care Financing Administr		not identified		Full-time-equivalent (FTE) nurse aides perresident, FTE RNs per resident,	not identif	not identif	restrained residents are morelikely to have higher

			n/a	n/a		case study (audit)		not identified		Effective change processe s can be	not identif	not identif	maintaini ng the safety of the
			21 acute geriatric nursesfro m 12 hospitals	n/a		semi-structure d interview s		not identified		Ethical decision-making incases of physical restraint	nursing as moral practice, patient safety, protectin g	protectio n of physical integrity, followed by psycholo	Nurses' decision-making in cases of physical restraint
			Nursing home staff and a hospital-based team of	n/a		secondar y data analysis (data collected by nursing		not identified		Residents in ECHO-AGE facilities were 75% less likely to	not identif	not identif	patient charactersitics - disruptive behaviour
			convenie nce sample of nursing staff in The Netherlan ds (n= 166), Germany (n= 258), and Switzerla nd (n=	n/a		question niare		not identified		In general, nursing staff held rather neutral opinions regarding the use of physical restraints , but assessed	not identif	culture	Dutch nursing staff consisten tly assessed restraint mea-sures as less restrictive than both German and
			173 nurses, trained nurse's aids and auxiliary	n/a		question niare		not identified		The most common reasons indicated for the use of	not identif	patient pro	the preventio n of disturban ce to other

			20 trainedan d untrained nursing staff from two Swiss	n/a		unstruct ured interview s		not identified		Three main themes wereextra cted from the data: (1) understa	not identif	safety and protectio n of resident, promotin g the well-	not identified
			all nursing staff on two nursing home	n/a		question niare		not identified		It is difficult to determin e whether	not identif	patient pro	falling, violent behaviou r, interferri ng with
			n/a	n/a		n/a		not identified		continue d phyiscal restraint	not identif	attitudes towards elderly and use	lack of educatio n, insufficie
			n/a	570 older persons (median age 86 years; 77.2%		survey		not identified		Neither staff intensity nor staff mix was a determin	not identif	not identif	patient character sitics - bathing depenenc y, transfer
			76 nursing homes (n= 5521) and 15hospit als (n= 2827)	n/a		secondar y data analysis of cross sectional survey data		not identified		The prevalenc e of restraints (bed rails and/or belts) was 9.3% for	not identif	not identif	more prevalent in women, older patients, patients with a high care depende
			administr ators and nursing staff in a	n/a		focus groups		not identified		Although responde ntslacked a shared definition	not identif	resident safety, prevent ligitation, paternalis	to prevent falls and other harazard

			One hundred eighty-nine physicians: in-terns in all	n/a		survey with vignettes		authors developed a framework involving ecology (situation), cues		The mean likelihood that physicians wouldorder	not identif	patient behaviour as overriding factor, patient safety - beliefs	not identified
			n/a	n/a		n/a		none mentioned		Six cluster-randomised controlled trials met the inclusion criteria. All studies investigated educational	not identif	not identif	inconclusive eidence about whether an education intervention is effective at reducing PR
			272 stakeholders	n/a		discussion forums and interviews		not identified		articipants identified legal concerns, existing	not identif	protection of patient safety, concerns about	not identified

			n/a	n/a		secondary analysis of an ex-isting data set of nursing home residents		A conceptual model for the use of restraints (Kayser-Jones, 1992) involving interaction		Lower cognitive status (OR 51.5 [for every 7-point decrease in Mini-Mental	not identif	not identif	Initiation of restraint occurred more often whenresidents were cognitivel
			20 trained and untrained nursing staff from two Swiss	n/a		unstructured interviews		not identified		Two main themes were extracted from the data: (i)	not identif	safety of older person and other residents in relation	not identified
			454 employees	488 clients from 12 residential homes for older people.		structured interviews, questionnaires		not identified		54% employees had committed at least one form of elder abuse - 65% had witnesse	Ethical principles were used - respect (e.g. violation of dignity), non-	not identified	stress of the job (burnout) understaffing, inadequate time for individual
			120 nurses from all units of	n/a		survey		theory of reasoned action		The research results indicate	theory of reasoned actions (nurses	not identified	not identified
			Eleven medical and surgical units in one 600-	One hundred seventy-four nursing homeresi		Secondary analysis of data obtained		not identified		Key findings suggest that organization	not identified	patient safety	organisational structures - understaffingor

			n/a	90 elderly residents		secondary data analysis & survey		not identified		the use of phySical restraints was not	not identified	not identified	Dependency level rather than ambulato

Authors	Year of publicatio n	Title	Aim of study	Review (yes / no)	Type of review	Country	Study context - maternity care, elderly population, physical restraint/mental health	Type of Mistreatment (physical, verbal, physical restraint)
Al-Maraira,	2019	Use of Restraint and Seclusion in Psychiatric Settings: A Literature Review	Review restraint studies to identify patients' perspectives and factors which influence use	YES	Umbrella	Multinatio nal; most reported in USA and Europe	pyschiatric patients	physical restraint

Ashcraft, L. and Anthony, W.	2008	Eliminating seclusion and restraint in recovery-oriented crisis services	describes aninitiative that began in 1999 attwo crisis centers that was de-signed to completely eliminatethe practice of seclusion and re-straint.	no	n/a	US (Arizona)	mental health crisis centre	physical restraint
Barbui, C. a	2020	Efficacy of interventions to reduce coercive treatment in mental health services: umbrella review of randomised evidence	Evaluate evidence on the efficacy of interventions to reduce coercive treatment	YES	Literature	n=19 Europe, n=4 USA	people with mental health conditions	physical restraint

Barr, L. and	Aug	Promoting positive and safe care in forensic mental health inpatient settings: Evaluating critical factors that assist nurses to reduce the use of restrictive practices	Determine how nurses' experiences can inform changes to reduce use of restrictive practices	NO	n/a	Australia	nurses in forensic mental health inpatient settings	physical restraint

Bower, F. L	2003	A synthesis of what we know about the use of physical restraints and seclusion with patients in psychiatric and acute care settings: 2003 update	Describe what is known about seclusion and restraint of psych patients and acute inpatient care	YES	narrative	US	psychiatric and acute care settings	physical restraint
Bowers, L.	2014	Safewards: a new model of conflict and containment on psychiatric wards	Describing the Safewards model for addressing risk and coercion in inpatient wards	NO	narrative	England	psychiatric and mental health nursing	physical restraint

Bowers, L. &	2014	Safewards: the empirical basis of the model and a critical appraisal	Review and evaluate evidence for the Safewards model	YES	narrative	n/a	psychiatric and mental health nursing	physical restraint
Bowers, L. &	Feb	Manual restraint and shows of force: the City-128 study	to assess the relationship of manual restraint and show of force to conflict behaviours, the use ofcontainment methods, service environment, physical environment, patient routines, staff characteris-tics, and staff group variables.	no	n/a	England	acute mental health care	physical restraint

Brophy, L.	2016	Consumers and Carer perspectives on poor practice and the use of seclusion and restraint in mental health settings: results from Australian focus groups	Present patient, carers, family member and support person perspectives related to restraint	NO	n/a	Australia	mental health setting	physical restraint
Chandler, (	2012	Reducing use of restraints and seclusion to create a culture of safety	Describe the structure that empowered staff to reduce the use of restraints	NO	n/a	US	psychiatric inpatient	physical restraint

Curran, S. S	2007	Staff resistance to restraint reduction: identifying & overcoming barriers	to explore literature on restance to reduction in reducing physical restraint	yes	narrative	n/a	psychiatir inpatient	physical restraint
De Benedic	2011	Staff perceptions and organizational factors as predictors of seclusion and restraint on psychiatric wards	Determine if staff perceptions related to violence on the ward predicted use of restraint	NO	n/a	Canada	psychiatric inpatient	physical restraint

Delaney, K.	2005	Patient characteristics and setting variables related to use of restraint on four inpatient psychiatric units for youths	Examine characteristics of children & adeolescents who were restrained	NO	n/a	USA	children and adolescents briefly hospitaied in a psych hospital	physical restraint
Gagnon, M	2013	Alternatives to seclusion and restraint in psychiatry and in long-term care facilities for the elderly: perspectives of service users and family members	Assessing alternatives to restraint for the elderly	NO	n/a	Canada	psychiatry service users and service user's families	physical restraint

Gerace, A. a	2019	Perceptions of nurses working with psychiatric consumers regarding the elimination of seclusion and restraint in psychiatric inpatient settings and emergency departments: An Australian survey	Nurses' perceptions on reducing & eliminating the use of restraint	NO	n/a	Australia	nurses working with psychiatric consumers	physical restraint
Hadi, F. and	2015	Predictors of physical restraint in a psychiatric emergency setting	Identify patients who are frequently restrained	NO	n/a	Iran	patients admitted to a psych hospital during the study period	physical restraint

Hammervo	2019	Post-incident review after restraint in mental health care -a potential for knowledge development, recovery promotion and restraint [illegible]	Explore the current knowledge of PIR to assess the extent to which it can minimize restraint use & harm	YES	Scoping	Sweden, UK, Canada, USA	mental health care	physical restraint
Hasan, A. A	2019	Psychiatric nurses' knowledge, attitudes, and practice towards the use of physical restraints	Examine knowledge, attitudes and practice of psych nurses	NO	n/a	Saudia Arabia	psychiatric nursing	physical restraint
Hawsawi, T	2020	Nurses' and consumers' shared experiences of seclusion and restraint: A qualitative literature review	to explore how nurses and consumers experienced seclusion andrestraint events in mental health care.	yes	qualitative	various	mental health care	physical restraint

Hopton, J.	1995	Control and restraint in contemporary psychiatric nursing: some ethical considerations	an evaluation ofthe ethical justifications for and the ethical and pohtical objections to the use ofphysical restraint techniques as a response to aggressive and self-injuriousbehaviour m contemporary mental health nursmg practice	yes	narrative	various	psychiatric nursing	physical restraint
Luiselli, J. K	2009	Physical restraint of people with intellectual disability: a review of implementation	Describe procedures to reduce and eliminate the use of physical restraint	YES	Literature	USA	people with intellectual disability	physical restraint
Riahi, S. an	2016	An integrative review exploring decision-making factors influencing mental health nurses in the use of restraint	To explore what influences mental health nurses' decision-making in the use of restraint.	yes	mixed	various	mental health nursing	physical restraint
Stewart, D.	2010	A review of interventions to reduce mechanical restraint and seclusion among adult psychiatric inpatients	To examine the nature and effectiveness of interventions to reduce use of mechanical restraint in adult psych patients	YES	Literature	various	adult psychiatric inpatients	physical restraint

Taxis, J. C.	2002	Ethics and praxis: alternative strategies to physical restraint and seclusion in a psychiatric setting	highlights a 42-month project in which a comprehensive program revision was implemented in a psychiatric hospital that included numerous alternative strategies to the use of patient restraint and seclusion.	no	n/a	US (Texas)	psychiatric hospital	physical restraint

**Theoretical perspective (i.e. constructivist, feminist) to undertake study**	**Study design (grounded theory/phenomenology/ qualitative exploratory, etc)**	**Participant characteristics - staff/professionals (age, profession, ethnicity, etc)**	**Participant characteristics - patients/service-users (age, ethnicity, etc)**	**Data collection methods**	**Key findings/ Themes**	**Explicit theories used to interpret findings (includes reference/clearly defined)**
not identified	review	n/a	n/a	n/a	The type of restraint used is reflective of the culture which the psych system exists; patients and staff view restraint differently	not identified

not identified	descriptive case study (secondary data analysis)	n/a	n/a	observation (case study	Existing records indicatedthat over a 58-month follow-upperiod (January 2000 to October2004), the larger crisis centertook ten months until a monthregistered zero seclusions and 31months until a month recordedzero restraints. The smaller crisiscenter achieved these same goalsin two months and 15 months, re-spectively. The success of this ini-tiative suggests that policy mak-ers and organizational leaders fa-miliarize themselves with theseand other similar seclusion andrestraint reduction strategies thatnow exist.	not identified
not identified	review	n/a	n/a	n/a	Different levels of evidence indicate the benefit of staff training, shared decision-making interventions and integrated care interventions to reduce coercive treatment in mental health services. These different levels of evidence should be considered in the development of policy, clinical and implementation initiatives to reduce coercive practices in mental healthcare, and should lead to further studies in both high- and low- income countries to improve the strength and credibility of the evidence base.	not identified

not identified	qualitative exploratory	nurse qualifications, gender, age, employment status, years of experiences	n/a	semi-structured interviews	Exposure to aggression can increase the use of restriction, most nurses gained expertise in the specialty via experience vs formalized education, leadership & training for less experienced staff is beneficial	not identified

not identified	review	n/a	n/a	n/a	The little that is known about restraint/seclusion use with these populations is inconsistent. Attitudes and perceptions of patients, family, and staff differ. However, all patients had very negative feelings about both, whether they were restrained/secluded or observed by others who were not restrained. The reasons for restraint/seclusion use vary with no accurate use rate for either. What precipitates the use of restraint/seclusion also varies, but professionals claim they are necessary to prevent/treat violent or unruly behavior. Some believe seclusion/restraint is effective, but there is no empirical evidence to support this belief.	not identified
not identified	evidence sythesis relating to model of containment	n/a	n/a	n/a	The Safewards Model is supported by the evidence, but that evidence is not particularly strong. There is a dearth of rigorous outcome studies and trials in this area, and an excess of descriptive studies. The model allows the generation of a number of different interventions in order to reduce rates of conflict and containment, and properly conducted trials are now needed to test its validity.	not identified

not identified	review	n/a	n/a	n/a	identified six originating domains as sources of conflict and containment: the patient community, patient characteristics, the regulatory framework, the staff team, the physical environment, and outside hospital.	not identified
not identified	cross sectional questionnaire	n/a	136 acute mental health wardswith their patients and staff in 67 hospitals in 26 NationalHealth Service trusts (organizational units with commonclinical policies and investment levels) in England, in2004–2005.	questionnaire	Manual restraint was used less frequently onEnglish acute psychiatric wards (0.20 incidents per day) than show of force (0.28 incidents per day).Both were strongly associated with the proportion of patients subject to legal detention, aggressivebehaviours, and the enforcement of treatment and detention. Medical, nursing, and security guardstaff provision were associated in different ways with variations in the use of these coercive interven-tions. An effective ward structure of rules and routines was associated with less dependence on thesecontrol methods. Training for manual restraint should incorporate the scenarios of attempted abscond-ing and enforcement of treatment, as well as violent behaviour. Attempts to	not identified

not identified	qualitative exploratory	n/a	The supporter focus groups consisted of 36 participants (29 women and seven men) who had experienced a family member or person close to them being secluded or restrained. These included parents, siblings, marital partners and two people who had advocacy roles. The consumer focus group consisted of 30 adults (13 [illegible]	focus groups	Patients view restraint as unnecessarily overused, restraint use creates & exacerbates problems for consumers, supporters, staff, system	Stigma (8)
appreciative inquiry	qualitative case study	n/a	Investigator's university and the study hospital aproved the study	Inductive content analysis	Nurse expertise increased with access to opportunities, information, support & resources	not identified

not identified	review	n/a	n/a	n/a	Professional organizations, regulating agencies, and hospital administrators have taken a strong stance on restraint reduction policies. When implementing a restraint reduction initiative, it is important to identify the barriers to restraint reduction, such as concern for personal safety, lack of knowledge about and practice using alternate de-escalation skills, and fear of disrupting the therapeutic milieu by using a variety of de-escalation methods	not identified
not identified	cross sectional questionnaire	309 staff members (nurses, rehabilitation instructors, and nurse's aides) providing care to patients with serious mental disorders were recruited from eight university psychiatric hospitals and general-hospital	n/a	questionnaire	Use of restraint is higher in intersive care & emergency departments, and when staff perceives anger & aggression; SES did ot predict use of restraint; staff education, title, and gender affect prevalence	not identified

not identified	secondary data analysis (hospital cards	n/a	n/a	examination of hospital charts	Youths who were at greatest risk of being restrained during brief inpatient treatment shared particular characteristics related to greater use of inpatient services, guardianship arrangements, special education placement, and history of suicide attempts. Inpatient staff members should remain particularly alert to the processing and regulation problems of these groups of patients.	not identified
not identified	qualitative exploratory	n/a	26 participants in short-term psychiatric care and 14 in long-term care facilities for the elderly and their families	Focus group discussions	participants emphasized the importance of communicating with service users, as well as assessing their needs and their particular situation, for reducing the use of restraint and seclusion. A welcome and accompaniment of people admitted for short-term psychiatric care emerged also as key approaches to reduce the use of restraint and seclusion. Long-term care facilities could also reduce the need for restraint and seclusion by creating a stimulating home environment and individualized occupational therapy programs.	not identified

not identified	cross sectional survey	Nurses (n = 512) across Australia	n/a	Anonymous survey	Restraint viewed as a necessary last resort measure to maintain safety and did not agree that it should be eliminated from practice. They acknowledge that use is deleterious; use is a function of lack of resources	not identified
not identified	case control study	n/a	files of 607 patients who were admitted during a one year period using convenience sampling; of [illegible]	Review of patient files	Patients admitted with meth induced psychotic disorder (MIP) & BPD I in manic episode had higher odds of being restrained; restraint is associated with longer stay; first admission more likely	not identified

not identified	review	n/a	n/a	n/a	No significant outcome related to PIR alone; can be used to enhance professional and ethical practice regarding restraint	not identified
not identified	cross sectional questionnair e	convenient purposive sample consisting of 110 nurses working in Al-Amal Mental Health [illegible]	n/a	Questionnair e	Moderate knowledge and attitudes about restraint; less than half recognized alternatives and most did not understand the reasons for restraint	not identified
not identified	review	n/a	n/a	n/a	six themes emerged under three main categories; shared experiences: disruption in care,disruption in the therapeutic relationship and shared negative impacts; nurses’ experiences:Absence of less coercive alternatives; and consumers’ experiences: overpowered, humiliated andpunished. indings suggested that consumers should receive recovery-oriented, trauma-informed andconsumer-centred care; while nurses should be better supported through personal, professionaland organizational developmental strategies.	not identified

not identified	review	n/a	n/a	n/a	the number of situations where control and restraint techniques are used might be reduced by the development of new therapeutic approaches. Such approaches should allow for more negotiation regarding care between clients and nurses, and acknowledge the potential benefits of clients resisting supposedly therapeutic interventions which they find unhelpful	not identified
not identified	review	n/a	n/a	n/a	Antecedent interventions to eliminate behaviors causing restraint and fixed time release have the most (still meagre) research validation	motivating operations (Friman & Hawkins 2006)
not identified	review	n/a	n/a	n/a	Eight emerging themes were identified: ‘safety for all’, ‘restraint as a necessary intervention’, ‘restraint as a last resort’, ‘role conflict’, ‘maintaining control’, ‘staff composition’, ‘knowledge and perception of patient behaviours’, and ‘psychological impact’.These themes highlight how mental health nurses’ decision-making is [illegible]	not identified
not identified	review	n/a	n/a	n/a	The interventions were diverse, but tended to include one or more of the following: new restraint and/or seclusion policies, staffing changes, staff training, review procedures and crisis management initiatives. The research was unable to address which of these elements was most effective. There was also evidence that some improved outcomes were achieved by substituting restraint or seclusion for each other or for alternatives formsof containment (medication in particular).	not identified

not identified	descriptive case study	n/a	n/a	observation (case study	Theresults of thi s project include a 94% reduction in th e rate ofrestraint and seclusion, development of extensive staf f andpatient education programs, and comprehensiveprogrammatic alterations consistent wit h a paradigm shift emphasizing collaboration, empowerment, and ethicalclinical practice.	not identified

**Theories/ideologies implicit by authors (but not references/explicity named)**	**Other interpretations used to explain the findings**
Patient autonomy, Stigma, Cultural variation, Therapeutic communication, Staff attitude & knowledge	not identified

patient safety, crisismode of management, reacting in-stead of responding to each situation,which only added to the crisis levels.Staff members often overlooked theconsumer's inherent strengths and re-sources because they were focusingprimarily on the problems.	lower-volume facility was able toachieve and maintain the goal of zerorestraint and seclusion long beforethe higher-volume facility because itwas not as crowded as the other facil-ity and because the layout and fur-nishings were more comfortable
Patient autonomy	The pre-existing mental health system will dictate whether results can be applied (i.e countries where human rights abuses occur, non-formal settings such as spiritual healing). staff training, shared decision-making interventions and integrated care interventions

occupational safety (Haineset al.2017)- Lack of post-graduate education (Koskinin et al, 2014)-- Limited cultural knowledge about indigenous peoples (Durey et al, 2014)-- Unit culture (Muir-Cochrane, 2018)	not identified

not identified	beliefs and attitudes of professionals that restriant is appropriate to protect patient
not identified	(1)staff team (2)physical environment (3)outside hospital (4)patient community (5)patient characteristics (6)regulatory framework

not identified	the patient community, patient characteristics, the regulatory framework, the staff team, the
patient safety, staff culture - less patient-centred, lack of training in alternatives	to enforce detention and treatment as it isto control aggression, medication refusal,coercedintramuscularmedication,andattemptedabsconding, as well as with aggressive patient behaviour
