# Supplementary material for: Theories for interventions to reduce physical and verbal abuse: A mixed methods review of the health and social care literature to inform future maternity care
Source: PLOS Glob Public Health. 2023 Apr 24;3(4):e0001594. doi: 10.1371/journal.pgph.0001594 (PMC10124898; doi:10.1371/journal.pgph.0001594)
Supplement: S4 Table — (PDF) [file pgph.0001594.s004.pdf]

ID	Authors	Year of pub	Title	Journal	Vol	Issue	Pages	Name & details of intervention	Name & details of comparator	Intervention based on theory (Yes/No/ Unsure)	Country	Study context - maternity care, elderly population, physical	Type of Mistreatment (physical, verbal, physical restraint)	Study design (RCT, non-randomised trial, Before & After etc)	Year when data was collected
**156**	Abraham, J. , Kupfer, R. , Behncke, A. , Berger-Hoger, B. , Icks, A. , Haastert, B. , Meyer, G. , Kopke, S. , Mohler, R.	2019	Implementation of a multicomponent intervention to prevent physical restraints in nursing homes (IMPRINT): A pragmatic cluster randomized controlled trial	International Journal of Nursing Studies	96		27-34	two versions of a guideline and theory-based multicomponent intervention (1. updatd version of guidelines; 2. concise version of guidelines 3. control usual care)	Optimized usual care	Yes	Germany	nursing homes	Physical	cluster RCT	2015
**157**	Ahmadi, M. , Bagheri-Saweh, M. I. , Nouri, B.Mohamadamini, O. , Valiee, S.	2019	Effect of Interventional Educational Programs on Intensive Care Nurses' Perception.	Critical Care Nursing Quarterly	42	1	106-116	2 day educational program for nurses about different types of restraint and issues related to its use	Before intervention	no	Iran	ICU	Physical	Before & After	N/A
**158**	,ersen, C. , Kolmos, A. , ,ersen, K. , Sippel, V. , Stenager, E.	2017	Applying sensory modulation to mental health inpatient care to	Nordic Journal of Psychiatry	71	7	525-528	Sensory Modulation (de-esculation method to prevent restraint &	usual care	Yes	Denmark	2 inpatient psychiatric mental health care (intervention); 1 unite as control	Combined	non-randomised control trial	2014-2015
**159**	Barton, S. A. , Johnson, M. R. , Price, L. V.	Jan	Achieving restraint-free on an inpatient behavioral health unit	Journal of Psychosocial Nursing & Mental Health Services	47	1	34-40	Patient centered restraint-free program: staff training, development of a preventative comfort room for patients, changes in staff attitude	Before intervention	Yes	NR	behavioural health inpatient unit	Combined	Before & After	2001-2008

**160**	Beaulieu, C. , Wertheimer , J. C. , Pickett, L. , Spierre, L. , Schnorbus, T. , Healy, W. , Palmer, C. , Jones, A.	2008	Behavior management on an acute brain injury unit: evaluating the effectiveness of an interdisciplinary training program	Journal of Head Trauma Rehabilitation	23	5	304-11	Nonviolent Crisis Intervention (NCI) program from the Crisis Prevention Institute; 4 day training in philosophy, prevention and safe management of disruptive behavior for instructors who go back to train staff in their facility	Before intervention	no	USA	acute barin injury unit	restraint use medication delivery	Before & After	2006-2007
**161**	Bell, A. , Gallacher, N.	2016	Succeeding in Sustained Reduction in the use of Restraint using the Improvement Model	BMJ Quality Improvement Reports	5	1		PDSA: Changes to reporting tool, staff debrief, education program, promote use of seated restraint	Before intervention	Yes	Fife, scotland	acute admission wards	Combined	Before & After	2012, 2014
**162**	Black, V. , Bobier, C. , Thomas, B. , Prest, F. , Ansley, C. , Loomes, B. , Eggleston, G. , Mountford, H.	May	Reducing seclusion and restraint in a child and adolescent inpatient area: implementation of a collaborative problem-solving approach	Australasian Psychiatry			1E+15	Collaborative problem solving (CPS) approach: familiarize staff, online staff training, prompt cards and posters, maintenance (training of new staff)	Before intervention	no	NZ	inpatient unit	Combined	Before & After	2016-2018

**163**	Blair, E. W. , Woolley, S. , Szarek, B. L. , Mucha, T. F. , Dutka, O. , Schwartz, H. I. , Wisniowski, J. , Goethe, J. W.	3	Reduction of Seclusion and Restraint in an Inpatient Psychiatric Setting: A Pilot Study	Psychiatric Quarterly	88	1	01-Jul	Intervention program: Broset Ciolence Checklist (BVC), staff education, frequent physician assessment of seclusion/resistant (S/R), formal review of S/R events, environmental enhancements	Before intervention	no	USA	psychiatric service	Combined	Before & After	2010-2012
**164**	Borckardt, J. J. , Madan, A. , Grubaugh, A. L. , Danielson, C. K. , Pelic, C. G. , Hardesty, S. J. , Hanson, R. , Herbert, J. , Cooney, H. , Benson, A. , Frueh, B. C.	May	Systematic investigation of initiatives to reduce seclusion and restraint in a state psychiatric hospital	Psychiatric Services	62	5	477-83	Engagement model with 4 components: trauma informed care training, changes in rules and language, patient involvement in treatment planning, and changes to the physical characteristics of the therapeutic environment. (3 month period)	Each unit served as its own control from intervention to intervention (usual care)	Yes	USA	psychiatric hospital	Combined	RCT	2005-2008
**165**	Bowers, L. , Brennan, G. , Flood, C. , Lipang, M. , Oladapo, P.	2006 (Apr)	Preliminary outcomes of a trial to reduce conflict and containment on acute psychiatric wards: City Nurses	Journal of Psychiatric & Mental Health Nursing	13	2	165-72	Two ‘City Nurses’ were employed to work	Before intervention	no	UK	acute psychiatric wards	Combined	Before & After	

**166**	Crosl, , K. A. , Cigales, M. , Dunlap, G. , Neff, B. , Clark, H. B. , Giddings, T. , Blanco, A.	2008	Using staff training to decrease the use of restrictive procedures at two facilities for foster care children	Research on Social Work Practice	18	5	401-409	with two acute wards for 1 year, assisting with the implementation of changes according to	Before intervention	no	USA	foster care facilities	Physical	Before & After	N/A
**167**	Dike, C. C. , Lamb-Pagone, J. , Howe, D. , Beavers, P. , Bugella, B. A. , Hillbr , , M.	2020	Implementing a program to reduce restraint and seclusion utilization in a public-sector hospital: Clinical innovations, preliminary findings, and lessons learned	Psychological Services	17		17	Eleven clinincal innovations based on 6 principles: (1) Leadership (2) Workforce development (3) Use of Restraint/Seclusion Prevention Tools (4) Data Informed Practice (5) Consumer Roles (6) Debriefing	Before intervention	no	USA	Psychiatric	Physical	Before & After	2009-2012
**168**	Donat, D. C.	2003	An analysis of successful efforts to reduce the use of seclusion and restraint at a public psychiatric hospital	Psychiatric Services	54	8	1119-23	Multiple efforts to reduce restraint: change criteria in administrative review of incidents, changes in case review committee composition, development of behavioral consultation team, enhance assessment standards, improve staff-patient ratio	Before intervention	no	USA	Psychiatric	Physical	Before & After	1997-2002

**169**	Duxbury, J. , Baker, J. , Downe, S. , Jones, F. , Greenwood, P. et al.	2019	Minimising the use of physical restraint in acute mental health services: the outcome of a restraint reduction programme ('REsTRAIN YOURSELF')	International journal of nursing studies	95		40â€48	REsTRAIN YOURSELF intervention: (UK) modified version of 'Six Core Strategies' (US) multimodal approach to reduce restraint - prevention & trauma informed principles	usual care	Yes	UK	Psychiatric	Physical	Non-random CT	2015-2016
**170**	Godfrey, J. L. , McGill, A. C. , Jones, N. T. , Oxley, S. L. , Carr, R. M.	2014	Anatomy of a transformation: a systematic effort to reduce mechanical restraints at a state psychiatric hospital	Psychiatric Services	65	10	1277-80	2 strategies: (1) staff training in deescalation techniques (2) policy change for the use of mechanical restraint	Before intervention	no	USA	Psychiatric	Physical	Before & After	2009-2012
**171**	Goulet, M. H. , Larue, C. , Lemieux, A. J.	2018	A pilot study of "post-seclusion and/or restraint review" intervention with patients and staff in a mental health setting	Perspectives in Psychiatric Care	54	2	212-220	Staff training: 2 presentations (15-30 min) followed by 1 month period to implement the process via answering questions daily	Before intervention	Yes	Canada	Psychiatric	Physical	Before & After	2014
**172**	Holstead, Jenell , Lamond, Diane , Dalton, Jim , Horne, Anita , Crick, Robert	2010	Restraint Reduction in Children's Residential Facilities: Implementation at Damar Services	Residential Treatment for Children & Youth	27	1	Jan-13	Multicomponent program consisting of employee training and resource management team	Before program	Unsure	USA	Care home	Physical	Before & After	2004-2008

**173**	Huizing, A. R. , Hamers, J. P. , Gulpers, M. J. , Berger, M. P.	2006	Short-term effects of an educational intervention on physical restraint use: a cluster randomized trial	BMC Geriatrics	6		17	An educational programme for nurses designed to promote restraint-free care and individualized care	usual care	no	Netherlands	Care home	Physical	RCT (cluster)	2003-2004
**174**	Huizing, A. R. , Hamers, J. P. , Gulpers, M. J. , Berger, M. P.	2009	A cluster-randomized trial of an educational intervention to reduce the use of physical restraints with psychogeriatric nursing home residents	Journal of the American Geriatrics Society	57	7	1139-48	An educational programme for selected staff to reduce restraint use; Five 2 hour educational sessions over 2 months plus a nurse specialist consultation	usual care	no	Netherlands	Care home	Physical	RCT	n/a
**175**	Huizing, A. R. , Hamers, J. P. , Gulpers, M. J. , Berger, M. P.	2009	Preventing the use of physical restraints on residents newly admitted to psycho-geriatric nursing home wards: a cluster-randomized trial	International Journal of Nursing Studies	46	4	459-69	An educational programme for selected staff to reduce restraint use inclusive of a nurse specialist consultation	usual care	no	Netherlands	Care home	Physical	RCT	n/a
**176**	Johnson, K. , Curry, V. , Steubing, A. , Diana, S. , McCray, A. , McFarren, A. , Domb, A.	2016	A non-pharmacologic approach to decrease restraint use	Intensive & Critical Care Nursing	34		12-Sep	power point review of non-pharmacological interven-tions and alternative devices with hands on demonstrationwith the devices were provided in the TICU on bothday and evening shift	Pre-intervention	no	USA	*Trauma intensive care unit*	Physical	Before & After	

**177**	Koczy, P. , Becker, C. , Rapp, K. , Klie, T. , Beische, D. , Buchele, G. , Kleiner, A. et al	2011	Effectiveness of a multifactorial intervention to reduce physical restraints in nursing home residents	Journal of the American Geriatrics Society	59	2	333-9	6 hour training course about restraint use to provide change agents with tools to minimize restraint use in situations of behavioral symptoms and injuries from falls	usual care	no	Germany	Care home	Physical	RCT	2004-2006
**178**	Kopke, S. , Muhlhauser, I. , Gerlach, A. , Haut, A. , Haastert, B. , Mohler, R. , Meyer, G.	2012	Effect of a guideline-based multicomponent intervention on use of physical restraints in nursing homes: a randomized controlled trial	JAMA	307	20	2177-84	A multidisciplinary approach designed to address attitudes, subjective norms and perceived behavioral control; evidence-based guideline, information programs, endorsement of nursing home leaders, and support materials	Standard information about restraint use and methods to avoid physical restraints delivered in three 12 to 24 page previously developed brochures	Yes	Germany	Care home	Physical	RCT	2009-2010
**179**	Lin, Y. L. , Liao, C. C. , Yu, W. P. , Chu, T. L. , Ho, L. H.	2018	A Multidisciplinary Program Reduces Over 24 Hours of Physical Restraint in Neurological Intensive Care Unit	Journal of Nursing Research	26	4	288-296	multidisciplinary PR reduction program	pre-intervention phasee	Yes	Taiwan	ICU	Physical	Before & After	

**180**	McCue, R. E. , Urcuyo, L. , Lilu, Y. , Tobias, T. , Chambers, M. J.	2004	Reducing restraint use in a public psychiatric inpatient service	Journal of Behavioral Health Services & Research	31	2	217-24	6 interventions primarily involving changing staff behavior: better identification of restraint, stress/anger management group for patients, staff training on crisis intervention, development of a crisis response team, daily review of all restraints and an incentive system for the staff	pre-intervention		USA	Psychiatric	Physical	Before & After	1999-2001
**181**	Pellfolk, T. J. , Gustafson, Y. , Bucht, G. , Karlsson, S.	Jan	Effects of a restraint minimization program on staff knowledge, attitudes, and practice: a cluster randomized trial	Journal of the American Geriatrics Society	58	1	62-9	A 6-month education progam for nursing staff; one volunteer attended 2 days of seminars and the remainder watched six 30 minute video lectures with focus groups	usual care	no	Sweden	Care home	Physical	RCT	n/a

**182**	Putkonen, A. , Kuivalainen, S. , Louheranta, O. , Repo-Tiihonen, E. , Ryynanen, O. P. , Kautiainen, H. , Tiihonen, J.	2013	Cluster-randomized controlled trial of reducing seclusion and restraint in secured care of men with schizophrenia	Psychiatric Services	64	9	850-5	staff, patients, and doctors were trained for six months in applying six core strategies to prevent seclusion-restraint; six months of supervised intervention followed	Not reported	no	Finland	Psychiatric	Physical	RCT	2009
**183**	Schreiner, G. M. , Crafton, C. G. , Sevin, J. A.	2004	Decreasing the use of mechanical restraints and locked seclusion	Administration & Policy in Mental Health	31	6	449-63	Multicomponent - Staff education and training; treatment interventions and system changes	Assessment phase before intervention	Unsure	USA	Psychiatric	Physical	Before & After	
**184**	Seckman, A. , Paun, O. , Heipp, B. , Van Stee, M. , Keels-Lowe, V. , Beel, F. , Spoon, C. , Fogg, L. , Delaney, K. R.	2017	Evaluation of the use of a sensory room on an adolescent inpatient unit and its impact on restraint and seclusion prevention	Journal of Child & Adolescent Psychiatric Nursing	30	2	90-97	Staff training encompassed the theory and principles of sensory, modulation guidelines to conducting a sensory room session, and handson familiarization with equipment. Training also included the steps to conducting a sensory room session and how to identify a patient who might be in need of the intervention.	pre-intervention	Yes	USA	Psychiatric	Physical	Before & After	2015

**185**	Singh, N. N. , Lancioni, G. E. , Karazsia, B.	2016	Effectiveness of Caregiver Training in Mindfulness-	Frontiers in Psychology	7		1549	Mindfulness-Based Positive Behavior Support (MBPBS) for caregivers	Training as usual		NR (first author is from USA)	Care home	Physical	RCT	
**186**	Singh, N. N. , Lancioni, G. E. , Karazsia, B. T. , Myers, R. E.	2016	Caregiver Training in Mindfulness-Based Positive Behavior Supports (MBPBS): Effects on Caregivers and Adults with Intellectual and Developmental Disabilities	Frontiers in Psychology	7		98	7-day intensive Mindfulness-Based Positive Behavior Support (MBPBS) training	pre-training	Yes	NR (first author is from USA)	Care home	Physical	Before & After	NR
**187**	Singh, N. N. , Lancioni, G. E. , Winton, A. S. W. , Singh, A. N. , Adkins, A. D. , Singh, J.	2009	Mindful staff can reduce the use of physical restraints when providing care to individuals with intellectual disabilities	Journal of Applied Research in Intellectual Disabilities	22	2	194-202	12 week mindfulness training programme	pre-training	Unsure	NR (first author is from USA)	Care home	Physical	Before & After	NR
**188**	Sivak, K.	2012	Implementation of comfort rooms to reduce seclusion, restraint use, and acting-out behaviors	Journal of Psychosocial Nursing & Mental Health Services	50	2	24-34	Use of comfort rooms	Before implementation	Yes	USA	Psychiatric	Physical	Before & After	2010-2011
**189**	Smith, N. H. , Timms, J. , Parker, V. G. , Reimels, E. M. , Hamlin, A.	2003	The impact of education on the use of physical restraints in the acute care setting	Journal of Continuing Education in Nursing	34	1	26-33; quiz 46-7	Comprehensive educational programme	pre-training	Yes	USA	Acute	Physical	Before & After	2000

**190**	Testad, I. , Aasl , , A. M. , Aarsl , , D.	2005	The effect of staff training on the use of restraint in dementia: a single-blind randomised controlled trial	International Journal of Geriatric Psychiatry	20	6	587-90	intervention consisted of a full day seminar, followed by a one-hour session of guidance per month over six months	usual care	Yes	Norway	Care home	Physical	RCT	
**191**	Testad, I. , Mekki, T. E. , Forl , , O. , Oye, C. , Tveit, E. M. , Jacobsen, F. , Kirkevold, O.	2016	Modeling and evaluating evidence-based continuing education program in nursing home dementia care (MEDCED)– training of care home staff to reduce use of restraint in care home residents with dementia. A cluster randomized controlled trial	International Journal of Geriatric Psychiatry	31	1	24-32	7 month training intervention “Trust Before Restraint"	usual care	Yes	Norway	Care home	Physical	RCT	2011-2013
**192**	Van Loan, Christopher L. , Gage, Nicholas A. , Cullen, Joseph P.	2015	Reducing Use of Physical Restraint: A Pilot Study Investigating a Relationship-Based Crisis Prevention Curriculum	Residential Treatment for Children & Youth	32	2	113-133	Relationship based crisis prevention curriculum	pre-training	Yes	USA	*Residential treatment program (camp)*	Physical	Before & After	NR

**193**	Wisdom, J. P. , Wenger, D. , Robertson, D. , Van Bramer, J. , Sederer, L. I.	2015 (Aug)	The New York State Office of Mental Health Positive Alternatives to Restraint and Seclusion (PARS) Project	Psychiatric Services	66	8	851-6	Training in, implementation of, and engagement with the Six Core Strategies to Reduce the Use of Seclusion and Restraint	pre-training	Yes	USA	Psychiatric	Physical	Before & After	2007-2011
**194**	Yeh, S. H. , Hsiao, C. Y. , Ho, T. H. , Chiang, M. C. , Lin, L. W. , Hsu, C. Y. , Lin, S. Y.	Sep	The effects of continuing education in restraint reduction on novice nurses in intensive care units	Journal of Nursing Research	12	3	246-56	Four hour restraint reduction lecture	pre-training	Yes	Taiwan	ICU	Physical	Before & After	NR

Sample strategy (random, consecutive etc.)	Sample size	Participant characteristics - staff/professionals (age, profession, ethnicity, etc)	Participant characteristics - patients/service-users (age, ethnicity, etc)	Data analysis methods	Included in Meta-Analysis	Evidence of effect of the intervention - Reduction in restraint	Intervention Point estimate	Intervention Standard deviation	Intervention 95% CI - lower limit	Intervention 95% CI - upper limit	Intervention Nos of participants	Comp Point estimate	Comp Standard deviation	Comp 95% CI - lower limit	Comp 95% CI - upper limit	Comp Nos of participants
Random	120 clusters		Age, Gender	Randomised at cluster level; intervention at practitioner level; analysed an individual participant level	Yes	physical restraint prevalence on cluster level (2 interventions) - (Proportion of residents with at least one physical restraint after 12 months)	1: -2.8<br>2: -3.9 (se comment in column AC for data for analysis - these are change scores not actual data)		1: -5.5<br>2: -6.8	1: -0.01<br>2: -1.0	1: 2984;<br>2: 2550	-1.2		-0.04	0.11	3307
	30 nurses	Nurses working in ICU with atleast a bachelor's degree and 1 year of work experience	Non specific reason for hospitalization		No	Mean score of nurses's (1) perception, (2)knowledge, (3)attitude, (4) practice of physical	(1)51.13; (2)11.20; (3)22.13; (4)26.33	(1)11.81; (2)1.58; (3)5.51; (4)2.17				(1)72.43; (2)7.06; (3)28.23; (4)21.20	(1)11.54; (2)1.85;(3)4.91; (4)4.86			
Cluster	2 Units with 17 beds; 25 staff members	Ooccupational therapists, nurses, social workers and medical staff	Men and women aged 18-64 with various psychiatric illnesses.	Allocated at cluster level; intervention at practitioner	No	Rate of use of physical restraint (nos of events per bed day)	0.0061	NR			5371	0.0106	NR			4627
	26-bed behavioral health inpatientunit within a private, nonprofit 248-bed community hospital	N/A	N/A		No	Number of patients restrained per fiscal year	0 [graph provided]					4				

	84 staff; 222 patient cases	Age; gender; profession; experience	Brain injury diagnosis; gender;		No	Percentage of restraint use	32 [estimated]In graph				148	36 (estimated from graph)				74
	30 bed Acute Admission ward	N/A	N/A		No	Median acute admissions rate of restraint	1.97 [graph provided]					4.18				
	16 bed unit; 16 full restraint, 29 partial restraint, 28 locked doors, 6 seclusion (baseline)	N/A	children and adolescents		No	Restrictive events	[full restraint] 80; [partial restraint] 226					[full restraint] 202; [partial restraint] 338				

	120 bed psychiatric service; 8029 patients compared to baseline n=3884	N/A	Age; gender; ethnicity		No	Rate of seclusion events	213				8029	358				3884
Random	446 patients; 340 staff			allocated at unit level and analysed at unit level	No	Mean seclusion and restraint rate (nos of seclusion or restraint incidents per patient day for each unit and each period)	0.005	0.002			446	0.27	0.018			446
					Yes	restrained mean per shift	0.032	0.197			1315	0.039	0.211			284

	44 employees	18 direct care staff, 1 supervisor; 13 female, 6 male (children's shelter); 9 direct care staff, 7 nurses, 3 therapists, 2 licensed nurse practitioners, 2 teachers, 1 occupational therapist, 1 psychologist; 19 female, 6 male (locked residential treatment facility)	foster children aged 18-18 years; boys and girls section(children's shelter); children aged 13-17, 60% foster children (locked residential treatment facility)		No	Frequency of mechanically restrictive procedures	refer to graph					refer to graph				
	615 bed psychiatric hospital	N/A	N/A		No	Mean annual restraint hours (per 100 patient days)	570					5300				
	unsure; multiple variables	Direct care clinicians, clinical department heads, hospital director, psychopharmacologist	Adult patients with severe and persistent psychiatric conditions (75% with schizophrenia/schizoaffective disorder); 2 thirds of patients have concurrent diagnosis		No	Seclusion and restraint hours per month	314					1344				

Paired Samples	2 acute care wards from 14 adult mental health wards		Mixed gender and single sex wards.	allocated and analysed at the cl;uster level	Yes	Restraint rate at adoption phase	6.62	6.7040816	5.53	7.72	7 wards, 144 beds	7.22	7.4540044	6.01	8.42	7 wards, 147 beds
	2910 acute adult unit patients; 334 community transition unit		psychiatric patients; 18-64 years old		Yes	mechanical restraint use over 2 sites (daily incidence rate) - 2 sites a: AAU; b: CTU	a: 0.01, b:0	a: 2.19, b:-			a: 1083; b:121	a: 0.57, b:0.09	a: 1.04, b: 0.45			a: 577 ; b:95
	12 staff; 195 patients	9 nurses, 3 orderlies; mean age= 41; average 16 years of experience in psychiatry			No	Prevalence of restraint (% of patients)	7.5					12.4				
	88 clients (2004); 125 clients (2008)				No	Restraints per child per year	3.66				125	56				88

Random	5 nursing home wards; 167 residents selected, 145 at endline	Nurses	Psycho-geriatric residents of both genders with dementia; average age 82.3 (control), 82.4 (experimental); 29% male, 71% female (control);		Yes	Mean restraint intensity post intervention	1.57	1.751			72	1.89	1.723			54
Cluster	15 nursing home wards; 432 residents selected, 241 at endline	Nursing staff; RN's, care workers, care helpers, care assistants	Psycho-geriatric residents of both genders; average age 83.4 (control), 82.0 (experimental); 20% male, 80% female (control); 22.2% male, 77.8% female (experimental)		Yes	Restraint intensity	In graph									
Cluster	14 nursing home wards; 138 residents selected, 105 at endline	Charge nurses, registered nurses, care workers, care helpers, care assistants, student care workers; average age 37, 80% women	Psycho-geriatric residents; average age 82.5 (control), 80.1 (experimental); 30% male 60% female (control); 35.5% male 64.7% female (experimental)	cluster randomised, intervention for staff, asessed at patient level	No	Restraint intensity post test 3	1.02	1.39			53	0.78	1.25			37
	77 nurses	Nurses gender 16% (9) males; 84% (47) female. Age 20-30u 20% (10), 31-40y 41% (21), 41-50y 31% (16), 51-60y 3% (2), 61-70 3% (2)			Yes	Mean Restraints per 1000 patient days	237.8	56.4				314.1	35.4			

Cluster	45 nursing homes; 430 restrained residents initially identified, 333 at endline	Registered nurses [40% with management roles], social workers	Severe cognitive impairment (median score 10/11 out of 16 on Dementia Screening Scale); 82.4% female, 17.6 % male (control), 71.2 % female, 28.8% (experimental); limited physical	cluster randomisation at nursing home level, intervention at staff level (change agents)	No	Reduction of duration of restraint use 100% OR	2.16		1.05	4.46						
Cluster	36 nursing homes (18 control, 18 intervention); 2166 residents (control), 2283 residents (intervention)	Fully trained nurses, nursing staff with 1 year training or on-the-job training	Average age 85 (control), 83 (experimental); 77% female* (control), 73% female (experimental) **no male value stated*	cluster randomised at nursing home level,	Yes	Prevalence of Physical Restraint Use at 6m follow up	22.6	91.512509	18.5	26.8	1868	29.1	89.881315	25	33.3	1802
	1291 patients		4 phases, Males: 521 (59.6%) 23 (57.5%), 101 (63.1%), and 135 (62.2%). Mean age for 4 phases; 54.5 (sd 20.5), 55.8 (sd 19.2), 53.5 (sd 21.1), and 56.4 (sd 21.4)		Yes	Physical restraint hours at maintenance phase	58.3	103.8			135	178.9	446.5			521

	6517 patients		Pre-intervention; Median age 35.5, male 59.7% (3892), female 40.3% (2625). Post intervention median age 36.9, male 58.4%(2875), female 41.6% (1761)		Yes	Number of restraints per 1000 patients days	3.7	1.93			4236	7.99	2.69			6517
Cluster	162 staff and 162 residents (control), 184 staff 191 residents (interventi on), at baseline; 133 staff and 165 residents (control),	Registered nurses, licensed practical nurses, and nursing aides; average age 41.8 (control), 43.5 (experimental); 90.7% female* (control), 89.7% female (experimental); 15.3 years in healthcare (control) 16.4 years in health care (experimental) *no male value stated*	Group home residents with dementia; Average age 83.4 baseline, 84 endline (control), 80.5 baseline, 81 endline (experimental); 78.4% female* (control), 69.1% female (experimental) *no male value stated*	cluster randomised at nursing unit level (20 to interv, 20 controls); intervetion at the staff level (interv 184 staff; control 162 staff); ouctomes patient	No	Risk of being restrained at follow-up OR	0.35		0.15	0.83						

Stratified cluster randomisation	2 intervention (50 beds) and 2 control wards (38 beds)		The mean & SD age of the patients was 40.2 &10.6 in the intervention cluster and 38.4 & 10.6 years in the control cluster.		No	Difference in proportion of patient-days with seclusion, restraint, or room observation	0.88	0.0721538	0.86	0.9	50	0.97	0.1258044	0.93	1.01	38
					No	Monthly average of full restraint events	21.43					37.67				
	52 staff	none reported			No	Restraint rate per 1000 patients days	20.3					27.6				

Random	77 caregivers	Age;gender of caregivers	Age; gender of patients		Yes	Use of physical restraint post treatment	0.53	0.9			24	10.77	3.21			24
NR	Staff: 33, Patients:18	Mean age 39, Male 17, female 16	Mean age 26, male 12, Female 6	Intervention to staff with outcomes at patient and staff level	Yes	Weekly frequency of staff use of physical restraints (over 40 weeks)	2	2.53			18	14	3.31			18
NR	Staff: 23, Patients:20	Mean age 47, Male 13, female 10	Mean age 26, Male 15, female 5		No	Mean number of physical restraints per week by AM and PM shift (Baseline v Practice)	AM:0.2, PM:0.35				20	AM: 2.67, PM: 2.60				20
NR	70 clients		28 women; 42 men. Clients age ranged 18-79		No	Average rate of restraint per 1000 days of client care	0					0.39				
NR	397 patients		NR		No	Average no. of days restrained	6.4					8.2				

random	55 in intervention group, 96 in control group		Age; gender of patients	Randomised at nursing home level	No	Use of restraints at follow-up score	1.5				55	3.7				87
Cluster	83 in intervention group, 114 in control group		Age; gender of patients		No	Multivariate logistic regressionof being subject to any restraint—OR	2.48									
NR	60 staff	Males, females 17			No	Average monthly frequency of restraint	5.5					17.3				

NR	NR		Age; gender of children and youth served by PARS trained mental health facilities		No	Number of restraint and seclusions per 1000 client days at 3 facilities	1:25, 2:7, 3:13					1:67, 2:63, 3:9				
NR	37 nurses	All female nurses, mean age 25.65			No	Clinical practice of restraint use (attitudes towards not practice)	28.05	3.59			37	28.41	3.28			37
